# Supplementary material for: The impact of photovoice on mental health and stigma: A systematic review and meta-analysis
Source: PLOS Glob Public Health. 2025 Jul 22;5(7):e0004272. doi: 10.1371/journal.pgph.0004272 (PMC12282929; doi:10.1371/journal.pgph.0004272)
Supplement: S1 Table — (DOCX) [file pgph.0004272.s001.docx]

**Full Search History on all databases.**

| **Database**(Limiters:2013 – 2023; peer-reviewed) | **No of hits** |
| --- | --- |
| **Cochrane trials** |  |
| Photovoice OR Photo novella OR Photography AND Mental health OR Mental illness | 50 |
| Title and Abstract review | **11** |
| **CINAHL Plus** |  |
| Effectiveness OR Efficacy OR Effective OR Success OR Outcome OR Evaluation OR Analysis OR Impact AND Photovoice OR Photo novella OR Photography AND Mental health OR Mental illness OR Mental Disorder OR Psychiatric illness | 71 |
| Title and Abstract review | **2** |
| **ProQuest** |  |
| Effectiveness OR Efficacy OR Effective OR Success OR Outcome OR Evaluation OR Analysis OR Impact AND Photovoice OR Photo novella OR Photography AND Mental health OR Mental illness OR Mental Disorder OR Psychiatric illness | 1045 |
| Title and Abstract review | **6** |
| **Medline** |  |
| Photovoice” OR “Photography” AND “Mental health” OR “Mental illness | 225 |
| Title and Abstract review | **2** |
| **PsychInfo** |  |
| Photovoice” OR “Photography” AND “Mental health” OR “Mental illness | 169 |
| Title and Abstract review | **2** |
| **Google Scholar** |  |
| Photovoice” OR “Photography” AND “Mental health” OR “Mental illness | 3500 |
| Title and Abstract review (first 300) | **2** |
| **Total** | 25 |
| Records after duplicates removed | **13** |
