# Supplementary material for: The impact of photovoice on mental health and stigma: A systematic review and meta-analysis
Source: PLOS Glob Public Health. 2025 Jul 22;5(7):e0004272. doi: 10.1371/journal.pgph.0004272 (PMC12282929; doi:10.1371/journal.pgph.0004272)
Supplement: S2 Table — (DOCX) [file pgph.0004272.s002.docx]

**Data Extraction Form**

| **DATA EXTRACTION FORM** | |
| --- | --- |
| **Study characteristics** | |
| Author/s |  |
| Publication date [Year] |  |
| Study title / citation |  |
| Record number / unique identifier |  |
| Study design |  |
| Aims/objectives |  |
| Inclusion criteria |  |
| Exclusion criteria |  |
| Sample size |  |
| Survey instrument |  |
| **Participant characteristics** | |
| Study Population |  |
| Age |  |
| Gender |  |
| Baseline Match |  |
| **Intervention** | |
| **Comparator** | |
| **Outcomes** | |
| **Power Calculation** | |
| Mean / Standard Deviation |  |
| Effect Size |  |
| **Results** | |
| **Notes** | |

Adapted from [1]

**References**

1. Tufanaru C, Munn Z, Aromataris E, Campbell J, Hopp L. Chapter 3: Systematic reviews of effectiveness. In: Aromataris E, Munn Z [Editors]*. JBI Manual for Evidence Synthesis.*JBI, 2020 and Centre for Reviews and Dissemination [2009] *CRD’s guidance for undertaking reviews in healthcare*. 3. ed. York: York Publ. Services [Systematic reviews].
