# Supplementary material for: The impact of photovoice on mental health and stigma: A systematic review and meta-analysis
Source: PLOS Glob Public Health. 2025 Jul 22;5(7):e0004272. doi: 10.1371/journal.pgph.0004272 (PMC12282929; doi:10.1371/journal.pgph.0004272)
Supplement: S2 Appendix — (DOCX) [file pgph.0004272.s006.docx]

**Reference list of included studies**

**References**

1. Zhang ZJ, Lo HH, Ng SM, Mak WWS, Wong SY, Hung KSY, et al. The Effects of a Mindfulness-Based Family Psychoeducation Intervention for the Caregivers of Young Adults with First-Episode Psychosis: A Randomized Controlled Trial. International Journal of Environmental Research and Public Health. 2023; 20[2]. doi:10.3390/ijerph20021018.

2. Flanagan EH, Buck T, Gamble A, Hunter C, Sewell I, Davidson L. “Recovery Speaks”: A Photovoice Intervention to Reduce Stigma Among Primary Care Providers. PS. 2016; 67[5]:566-569. doi: 10.1176/appi.ps.201500049.

3. Russinova Z, Gidugu V, Bloch P, Restrepo-Toro M, Rogers ES. Empowering individuals with psychiatric disabilities to work: Results of a randomized trial. Psychiatr Rehabil J. 2018 Sep; 41[3]:196-207. doi:10.1037/prj0000303.

4. Russinova Z, Gidugu V, Rogers ES, Legere L, Bloch P. Fostering the community participation of individuals with psychiatric disabilities: Effectiveness of a new peer-led photovoice-based intervention. Psychiatric rehabilitation journal. 2023 Sep 1; 46[3]:196-210. doi:10.1037/prj0000540.

5. Russinova Z, Rogers ES, Gagne C, Bloch P, Drake KM, Mueser KT. A Randomized Controlled Trial of a Peer-Run Antistigma Photovoice Intervention. PS. 2014; 65[2]:242-246. doi: 10.1176/appi.ps.201200572.

6. Kohrt BA, Jordans MJD, Turner EL, Rai S, Gurung D, Dhakal M, et al. Collaboration with People with Lived Experience of Mental Illness to Reduce Stigma and Improve Primary Care Services: A Pilot Cluster Randomized Clinical Trial. JAMA Netw Open [Internet]. 2021 Nov 3 [cited 2023 Jun 2];4[11]: e2131475. doi: 10.1001/jamanetworkopen.2021.31475.

7. Tippin GK, Maranzan KA. Efficacy of a Photovoice-based video as an online mental illness anti-stigma intervention and the role of empathy in audience response: A randomized controlled trial. J Appl Soc Psychol. 2019; 49[6]:381-394. doi:10.1111/jasp.12590.
