# Supplementary material for: The impact of photovoice on mental health and stigma: A systematic review and meta-analysis
Source: PLOS Glob Public Health. 2025 Jul 22;5(7):e0004272. doi: 10.1371/journal.pgph.0004272 (PMC12282929; doi:10.1371/journal.pgph.0004272)
Supplement: S3 Appendix — (DOCX) [file pgph.0004272.s007.docx]

**Funnel plots for all outcomes.**

Depression Recovery

Self-Efficacy Social Withdrawal

Stigma Resistance Anger

Perception of Danger Social Distance
