# Supplementary material for: The impact of photovoice on mental health and stigma: A systematic review and meta-analysis
Source: PLOS Glob Public Health. 2025 Jul 22;5(7):e0004272. doi: 10.1371/journal.pgph.0004272 (PMC12282929; doi:10.1371/journal.pgph.0004272)
Supplement: Attachment — Submitted filename: PGPH-D-24-00689_R1_reviewer.pdf [file pgph.0004272.s010.pdf]

PLOS Global Public Health

The impact of photovoice on mental health and stigma: A systematic review and meta-analysis.

--Manuscript Draft--

Manuscript Number:	PGPH-D-24-00689R1
Article Type:	Research Article
Full Title:	The impact of photovoice on mental health and stigma: A systematic review and meta-analysis.
Short Title:	Photovoice and mental health.
Corresponding Author:	Ugochukwu Emmanuel Aghalu, MPH<br>Glasgow Caledonian University School of Health and Life Sciences<br>Glasgow, UNITED KINGDOM OF GREAT BRITAIN AND NORTHERN IRELAND
Order of Authors:	Adeniyi Adeboye, MD, Ph.D
	Ugochukwu Emmanuel Aghalu, OD, MPH
	Whitney Onuorah, MD, MPH
	Chizor Samuel-Nwokeji, OD, MPH
	Chimaobi Nwanguma, Msc. MPH
	Adekunle Akerele, Msc, PhD
	Judy Wasige, Ph.D
Keywords:	Photovoice; mental health; meta-analysis; people living with mental illness; health care providers; depression; recovery; self-efficacy; social withdrawal; stigma; stigma-resistance; anger; social distance; perception of danger; PLWMI.
Abstract:	Background and Objective: Photovoice is a transformative tool for improving health outcomes among people living with mental ill-health (PLWMI), fostering awareness, community engagement, empowerment, and social change. Importantly, only one meta-analysis has evaluated the effectiveness of photovoice for mental health outcomes. However, the study focused on various health conditions and only addressed two mental health outcomes. This systematic review and meta-analysis, by exclusively evaluating the effect of photovoice on eight mental health and stigma-related outcomes in PLWMI and healthcare providers (HCPs), provides a comprehensive overview of the impact of photovoice on mental ill health and new evidence for practice.<br>Methods: Electronic database searches were conducted on Cochrane Trials, CINAHL Plus, ProQuest, Medline, and PsychInfo. Additionally, Google Scholar was searched to aid the retrieval of both published and unpublished Randomised Control Trials testing the effects of photovoice on PLWMI, HCPs or both from 2013 to 2023. Random-effects meta-analyses were conducted for all outcomes evaluated in the study. A within-sample estimation of the effect size (Cohen's d) was calculated using the available raw data (mean and standard deviation) from each arm of each eligible study. The study protocol was pre-registered on PROSPERO, with registration number CRD42023476867.<br>Findings: Seven studies (Sample size (n)=754) were included in the meta-analysis. The findings revealed that the application of photovoice resulted in a significant decrease in various mental health outcomes among PLWMI, including social withdrawal (3 studies, n=318; Cohen's d (d), 95% CI= -0.2, -0.41 to 0.00), depression (2 studies, n=267; d, 95% CI= -0.23, -0.45 to -0.02). Conversely, there was a significant increase in self-efficacy (2 studies, n=267; d, 95% CI= 0.22, 0.00 to 0.44) and recovery (3 studies, n=285; d, 95% CI= 0.19, -0.02 to 0.41). Additionally, the stigma-related outcomes of PLWMI such as stigma resistance also decreased (3 studies, n=318; d, 95% CI= -0.13, -0.36 to -0.10). Furthermore, there was a significantly large reduction in the mental health-related outcome (anger) demonstrated by HCPs towards PLWMIs (2 studies, n=330; d, 95% CI= -1.05, -2.79 to 0.69). Additionally, there were also significantly large reductions in social distance (3 studies,

	n=418; d, 95% CI= -1.41, -3.02 to 1.03) and perception of danger (2 studies, n=115; d, 95% CI= -1.80, -5.04 to 1.44) which were classified as stigma-related outcomes in HCPs.<br>Interpretation: The meta-analysis demonstrated that photovoice significantly improved several mental health outcomes in PLWMI. Specifically, photovoice was effective in reducing social withdrawal and depression among PLWMI. Moreover, the intervention led to significant increases in self-efficacy and recovery of PLWMI. In terms of stigma-related outcomes, photovoice resulted in a reduction in stigma resistance among PLWMI. In healthcare providers (HCPs), the findings indicated a significant reduction in negative mental health-related outcomes, such as anger towards PLWMI. Additionally, there were substantial decreases in social distance and the perception of danger, which are stigma-related outcomes in HCPs. Consequently, incorporating photovoice into mental health interventions could enhance patient mental health outcomes and reduce HCP-associated stigma, promoting better outcomes in PLWMI.
**Additional Information:**	
**Question**	**Response**
**Financial Disclosure**<br><br>Enter a financial disclosure statement that describes the sources of funding for the work included in this submission and the role the funder(s) played. This includes grants and any commercial funding of the work or authors.<br><br>This statement will be typeset if the manuscript is accepted for publication.<br><br>*Please review the submission guidelines and the instructions link below for detailed requirements and guidance.*	This work was supported by See Me Scotland (URL: https://www.seemescotland.org/). There was no specific grant number associated with the grant. JW was the specific recipient of the award. CN, WO, and CS received compensation. “The funders had no role in study design, data collection and analysis, decision to publish, or preparation of the manuscript.”
**Competing Interests**<br><br>On behalf of all authors, disclose any competing interests that could be perceived to bias this work.<br><br>This statement will be typeset if the manuscript is accepted for publication.<br><br>*Please review the instructions link below and PLOS Global Public Health’s competing interests policy to determine what information must be disclosed at submission.*	The authors have read the journal’s policy and hereby declare no financial or non-financial conflicts of interest.

**Data Availability**<br><br>Before publication, Authors are required to make fully available and without restriction all data underlying their findings. Please see our PLOS Data Policy page for detailed information on this policy.<br><br>A **Data Availability Statement**, detailing where the data can be accessed, is required at first submission. Insert your Data Availability Statement in the box below.<br><br>Please see the data reporting section of our submission guidelines for instructions on what you need to include in your Data Availability Statement.<br><br>This statement will be typeset if the manuscript is accepted for publication.<br><br>*PLOS allows rare exemptions to address legal and ethical concerns. If you have legal or ethical restrictions, please use the box below to detail these in full sentences for the Journal team to consider.*	All necessary data have been included as part of the manuscript submission.

Powered by Editorial Manager® and ProduXion Manager® from Aries Systems Corporation

Manuscript Click here to access/download;Manuscript;current final manuscript PDF.pdf

The Impact of Photovoice on Mental Health and Stigma: A systematic review and meta-analysis.

Photovoice and mental health.

Adeniyi Adeboye[1¶], Whitney Onuorah[1&], Chizor Samuel-Nwokeji[1&], Ugochukwu Aghalu [1*], Chimaobi Nwanguma[1], Adekunle Akerele[2], Judy Wasige[1¶]

1 Department of Nursing and Community Health, School of Health and Life Sciences, Glasgow Caledonian University, Glasgow, Scotland, United Kingdom

2 Department of Epidemiology, Medical Statistics and Environmental Health, College of Medicine, University of Ibadan, Ibadan, Nigeria.

* Corresponding author

E-mail: UAGHAL300@caledonian.ac.uk (UA)

¶These authors contributed equally to this work.

&These authors also contributed equally to this work.

Abstract

Background and Objective: Photovoice is a transformative tool for improving health outcomes among people living with mental ill-health (PLWMI), fostering awareness, community engagement, empowerment, and social change. Importantly, only one meta-analysis has evaluated the effectiveness of photovoice for mental health outcomes. However, the study focused on various health conditions and only addressed two mental health outcomes. This systematic review and meta-analysis, by exclusively evaluating the effect of photovoice on eight mental health and stigma-related outcomes in PLWMI and healthcare providers (HCPs), provides a comprehensive overview of the impact of photovoice on mental ill health and new evidence for practice.

Methods: Electronic database searches were conducted on Cochrane Trials, CINAHL Plus, ProQuest, Medline, and PsychInfo. Additionally, Google Scholar was searched to aid the retrieval of both published and unpublished Randomised Control Trials testing the effects of photovoice on PLWMI, HCPs or both from 2013 to 2023. Random-effects meta-analyses were conducted for all outcomes evaluated in the study. A within-sample estimation of the effect size (Cohen's d) was calculated using the available raw data (mean and standard deviation) from each arm of each eligible study. The study protocol was pre-registered on PROSPERO, with registration number CRD42023476867.

Findings: Seven studies (Sample size (n)=754) were included in the meta-analysis. The findings revealed that the application of photovoice resulted in a significant decrease in various mental health outcomes among PLWMI, including social withdrawal (3 studies, n=318; Cohen's d (*d)*, 95% CI= -0.2, -0.41 to 0.00), depression (2 studies, n=267; *d*, 95% CI= -0.23, -0.45 to -0.02). Conversely, there was a significant increase in self-efficacy (2 studies, n=267; *d,* 95% CI= 0.22, 0.00 to 0.44) and recovery (3 studies, n=285; *d,* 95% CI= 0.19, -0.02 to 0.41). Additionally, the stigma-related outcomes of PLWMI such as stigma resistance also decreased (3 studies, n=318; *d*, 95% CI= -0.13, -0.36 to -0.10). Furthermore, there was a significantly large reduction in the mental health-related outcome (anger) demonstrated by HCPs towards PLWMIs (2 studies, n=330; *d*, 95% CI= -1.05, -2.79 to 0.69). Additionally, there were also significantly large reductions in social distance (3 studies, n=418; *d*, 95% CI= -1.41, -3.02 to 1.03) and perception of danger (2 studies, n=115; *d,* 95% CI= -1.80, -5.04 to 1.44) which were classified as stigma-related outcomes in HCPs.

Interpretation: The meta-analysis demonstrated that photovoice significantly improved several mental health outcomes in PLWMI. Specifically, photovoice was effective in reducing social withdrawal and depression among PLWMI. Moreover, the intervention led to significant increases in self-efficacy and recovery of PLWMI. In terms of stigma-related outcomes, photovoice resulted in a reduction in stigma resistance among PLWMI. In healthcare providers (HCPs), the findings indicated a significant reduction in negative mental health-related outcomes, such as anger towards PLWMI. Additionally, there were substantial decreases in social distance and the perception of danger, which are stigma-related outcomes in HCPs. Consequently, incorporating photovoice into mental health interventions could enhance patient mental health outcomes and reduce HCP-associated stigma, promoting better outcomes in PLWMI.

Introduction

Photographs have been recognised as prominent sources of visual data [1], and narrative photography; a principle used in photovoice, which unveils the latent meaning of an image from the perspective of the reporter using storytelling, has garnered widespread adoption in research in recent times [2]. This is grounded in Goffman's symbolic interactionism, which posits that meanings are derived from people's interaction with symbols and other individuals [3]. Photovoice lends a crucial perspective in healthcare as it enables individuals to creatively elaborate the nuances of their health issues and lived experiences [4].

Notwithstanding an increased awareness of mental health conditions, there is still a deficit of knowledge on how people with mental health issues deal with the challenging stressors associated with their conditions amidst their daily lives. This impedes the provision of relevant and adequate support [5]. According to extant studies, about 50-85% of the world population's mental health issues are currently untreated resulting in a high global burden of the condition [6]. Intentional avoidance of treatment due to stigma from healthcare providers (HCPs) experienced by people living with mental health illness (PLWMI) has been reported as one of the enabling factors of the increasing number of untreated cases[7]. Consequently, explicit stigma demonstrated by HCPs towards PLWMI has been linked with the underutilization of mental health care services among this population [8,9]. Likewise, implicit stigma in HCPs has been related to reduced detection of mental illness cases due to inadequate assessment of potential cases due to prejudice and discrimination [9,10]. This explicit or implicit stigma faced by PLWMI results in hopelessness, low self-esteem, and reduced empowerment, quality of life, as well as social support, which gravely interferes with recovery [11,12].

Over the years, increased efforts have been made to develop tools that would sufficiently support individuals with mental health conditions [13], while combatting stigma, internalization of negative stereotypes, and enhancement of self-efficacy [14]. Photovoice has been shown to have an

inherent fluidity that enables its application as a methodology used in exploring various aspects of the lived experiences of people with mental ill health. The establishment of its methodology on Participatory Action Research (PAR) principles enables participants to play the role of collaborators and coresearchers, which asserts their wealth of experience in the conceptualization and development of suitable support to address their needs [1,15].

Photovoice avails PLWMI an innovative opportunity to tell their story, which aids in improving awareness of their lived experiences amongst the policymakers, service providers and other stakeholders, in a bid to drive social change [1,11,16]. Photovoice can be utilised in two major ways, functioning both as a powerful intervention for improving mental health and as a robust qualitative methodology for eliciting outcomes suitable for quantitative evaluation [17]. As an intervention, one of the fundamental strengths of photovoice is its ability to transfer power to participants. By allowing individuals to document their lived experiences through photography, photovoice democratizes the research process, shifting control from the researcher to the participant [18]. This empowerment is particularly therapeutic for individuals with mental health issues, as it validates their experiences and provides a platform to voice their narratives [12]. This transfer of power is crucial in mental health interventions, where feeling heard and understood can significantly impact one's mental well-being [12].

Analysing the methodological underpinnings of photovoice, the data gathered through photovoice can uncover significant variables and outcomes that might be overlooked in traditional mental health research [19]. Administering photovoice involves a series of steps designed to engage participants in capturing and reflecting on their lived experiences through photography. This process typically begins with participant recruitment and an orientation session where the aims of the study, ethical considerations, and technical aspects of photography are discussed [20]. Participants are then tasked with taking photographs that represent their perspectives on a given issue, followed by a series of group discussions where they share their images and narratives. These discussions are crucial for

co-constructing meaning and ensuring that the participants' voices are authentically represented [21]. The facilitators play a significant role in guiding these discussions, encouraging deep reflection, and maintaining a non-judgmental space. One of the challenges in this process is ensuring that all participants are equally engaged and that power dynamics do not skew the interpretation of the visual data [22]. Moreover, the analysis of photovoice data requires a nuanced approach that considers both the visual and textual elements, often demanding interdisciplinary expertise [23]. The insights derived from participants' photographs and subsequent discussions can inform the development of more targeted and effective quantitative outcomes [19]. For example, integrating photovoice methodology with surveys or standardized assessment tools can help quantify changes in mental health outcomes, such as reductions in anxiety or improvements in self-esteem. Additionally, photovoice methodology has been used to identify community-specific issues, such as the need for better HIV/AIDS education or the challenges faced by immigrant adolescents, which can then be quantified and addressed through targeted interventions [24].

Subsequently, photovoice's dual role as both an intervention for mental health and a methodology for quantitative outcome elicitation underscores its versatility and effectiveness. Its capacity to empower participants, raise consciousness, and foster community engagement, combined with its ability to generate rich qualitative data that can inform quantitative measures, makes photovoice a uniquely valuable tool in advancing mental health research and practice. Through this integrated approach, photovoice not only addresses immediate mental health needs but also contributes to long-term social change and policy development.

Consequently, this study evaluates the impact of photovoice on mental ill health. It reviews appraised articles that report the effect of photovoice on the mental health outcomes of PLWMI such as depression, recovery, self-efficacy, and social withdrawal, as well as reducing negative behaviours exhibited by HCPs towards PLWMI that exacerbate mental ill health, such as anger. Furthermore, the study also assesses the impact of photovoice on the enhancement of positive stigma-related outcomes

in PLWMI, including stigma resistance, in addition to the reduction of negative behaviours of HCPs that perpetuate feelings of stigma in PLWMI, such as the perception of danger and social distance.

In the context of this paper, mental health outcomes refer to outcomes that assess the internal psychic phenomena of patients, which cannot be externally examined or confirmed [25]. These measures typically assess either the frequency and severity of symptoms related to mental health illnesses (psychopathological rating scales), or evaluate the impact of mental health illnesses on an individual (measures of social functioning, or quality of life assessment) [25]. Furthermore, stigma-related outcomes refer to the various consequences or impacts that stigma has on individuals living with mental illness (PLWMI), and their ability to overcome such consequences [26]. These outcomes can affect multiple aspects of a person's life, including their mental health, social interactions, access to healthcare, and overall quality of life [26].

The current study expatiates on the works of Halvorsrud *et al.* [4], who conducted a systematic review and meta-analysis investigating the impact of photovoice on a range of health conditions such as diabetes, hepatitis, physical health, social function, and mental health. However, this is the first study to elaborately focus on investigating the facets of mental health and stigma as they relate to PLWMI and their HCPs, with analysis of eight key outcomes: recovery, self-efficacy, stigma resistance, social withdrawal, depression, anger, social distancing, and perception of danger. This provides a comprehensive overview of the effectiveness of photovoice in mental health and informs new evidence for practice.

The study looks to answer the question "What is the impact of photovoice on mental health and stigma outcomes of PLWMI and HCPs"? To this effect, four hypotheses were formulated. Firstly, the authors hypothesised that photovoice has a significant effect on improving the mental health outcomes of PLWMI. Secondly, it was hypothesised that photovoice significantly reduces the negative mental health-related behaviours of HCP towards PLWMI. Thirdly, it was hypothesised that

photovoice has a significant effect on improving the positive stigma-related outcomes of PLWMI. Fourthly, it was also hypothesised that photovoice significantly reduces the negative stigma-related behaviours of HCP towards PLWMI. Therefore, this study analysed the effectiveness of photovoice on the mental health and stigma outcomes of PLWMI and the behaviours of HCPs towards PLWMI.

Methods

This review employed a systematic review and meta-analysis to investigate the effectiveness of photovoice in improving mental health outcomes among PLWMI and the attitudes of their HCP. It was pre-registered on PROSPERO with identification number - CRD42023476867. A systematic review provides a comprehensive summary of evidence regarding a specific research focus through a systematic approach to identify, appraise, and synthesize all relevant evidence [27,28]. Additionally, meta-analysis involves statistical analysis that combines and synthesizes the results of numerous studies to integrate their findings and produce a quantitative estimate [29,30]. This proffers increased accuracy in effect estimation, conflict resolution between studies of the same foci, and generalizability of research findings [31,32].

Eligibility Criteria

Types of studies

Primary quantitative experimental studies, exclusively randomized control trials or mixed methods studies incorporating a qualitative photovoice-based study as an intervention in a randomized controlled design, were included. [33,34]. The population of interest were PLWMI and their healthcare providers. No restrictions were implemented on type of mental illness, age, or setting, and studies addressing mental health outcomes such as depression, recovery, self-efficacy, social withdrawal, stigma, and stigma-resistance and published in the English language from January 2013 to November 2023 were included.

Only studies with explicitly stated photovoice methodology, or participatory photography methodology that entailed training participants on photography and facilitating focused group discussions on photography and mental health were included. PLWMI would take photographic images that depicted their lived experiences of mental ill-health and interpret the photographs to the researcher to determine the meanings attributed. Participants were co-researchers and solely responsible for conceptualizing and allocating meaning to the pictures, as well as selecting photos and uncovering the stories behind them to the researchers [15,16,20].

Included studies with healthcare practitioners measured the effectiveness of photovoice in ameliorating negative attitudes towards PLWMI. Excerpts from photovoice participatory studies would be introduced to some healthcare practitioners as an intervention and compared with others where there was either no intervention or practice as usual on anti-stigma interventions.

Search strategy and study selection

A scoping search was carried out on Cochrane Trials, CINAHL Plus, and Google Scholar to determine the availability of studies addressing the research question, inform the search development process, and keywords used in relevant articles [35]. Between October to November 2023, five academic databases– Cochrane Trials, CINAHL Plus, ProQuest, Medline, and PsychInfo, were systematically perused for relevant papers. Google Scholar search engine was also incorporated to identify grey literature, and minimize publication bias [36,37], which promulgates comprehensiveness of the search process and evidence base, as well as minimizes omission of otherwise relevant studies which may not be present on generic databases [38,39]. Covidence automation software, alongside manual data management, was incorporated in this review to ensure completeness in the retrieval of all available data, transparency, and bias reduction in the review process. Two reviewers, CN and JW independently screened the titles and abstracts of identified studies against the eligibility criteria, and an agreement was reached on the articles for full-text screening. Additionally, the reference lists of

relevant articles were manually scanned for relevant papers. Concomitantly, CSJ and WO utilized Covidence for citation screening, data extraction and quality appraisal, which facilitated the reliability and rigour of the review process [40]. The search process and study selection for inclusion were stringently guided by the Preferred Reporting Items for Systematic Reviews and Meta-Analyses (PRISMA), for which a flow diagram was generated to aid transparency and reproducibility [41,42].

Data extraction

Data extraction was carried out independently by two researchers, CSJ and WO on Covidence. A form was developed from manuals of the Centre for Reviews and Dissemination, Joanna Briggs Institute, and consultation with experts in the subject area [43,44]. This form was piloted on two included studies to ascertain suitability and adjusted as required [45]. Data was extracted in textual, numerical, and tabular formats, and a third reviewer, AA, was invited to resolve any conflicts. This was done to ensure quality control, completeness, and reliability of extracted data [46]. The data extraction focused on study characteristics (publication year, author, design, aim, sample size, survey instrument); participant characteristics (age, gender); intervention (photovoice and comparator); study outcome and effect size (mean, standard deviation, power calculation). Furthermore, AA and AA transferred the data into Microsoft excel which was already prepared with appropriate and relevant columns detailing shortlisted studies that met the eligibilities criteria. Variables extracted from these studies included mean/SD of study outcomes as well as effect sizes where they are available. Extracted data were shared with the members of the research team for second level of data verification before analysis was conducted. Standard error of each effect size was estimated using recommended formula as none was available in all the eight eligible studies [47].

Quality assessment

Quality appraisal was done using a validated tool for quantitative studies, the Effective Public Health Practice Project [EPHPP] [48] on Covidence. This assessed quality within six domains - selection bias; study design; confounders; blinding; data collection methods; withdrawals and dropouts. These result in an overall global rating, wherein studies with no weak ratings amongst the six domains are considered 'strong,' studies with one weak rating are considered 'moderate,' and studies with two or more weak ratings are considered 'weak.' Two reviewers, CSJ and WO, independently assessed quality, and discordances were resolved through discussion, or by a third reviewer, AA.

Data Analysis

A meta-analysis was conducted to pool studies with similar outcomes. This was done in two phases; one phase focused on studies on positive and negative mental health outcomes of PLWMI, and the other on studies on positive and negative attitudes of healthcare providers towards PLWMI.

Quality control was done and maintained by members of the review team to ensure validity. Raw data (i.e. mean and standard deviation of the two arms of each study) were used to calculate effect size from all the included studies using the recommended formula. This is a preferred approach to calculating effect size due to inconsistency in reporting the precalculated effect size across the studies extracted. This approach gives a concise estimate and direction of the effect size [49]. Based on the RCT study design of each eligible study with a focus on variable outcomes of interest, a within sample estimation of photovoice effectiveness was calculated using mean/standard deviation of each arm of each eligible study. Results reported included confidence intervals of effect size of each outcome, and overall effect size of the study outcome (Cohen's d). Due to expected differentials of the studies, random effect model was used for calculation of overall effect size of photovoice on each outcome

[50]. Z distribution of 95% confidence intervals (CIs) was employed to estimate overall effect size. This approach is said to offer a parsimonious and narrower CI to the study outcomes when one considers the available sample size of at least two studies per outcome [50]. To reduce the effect of disparity and to normalize the data, a log transformation of the precalculated effect size was conducted.

The I squared statistic (I^2) and Hegger test from forest plots were used to assess the heterogeneity of the precalculated effect size from various studies. Funnel plots were used to assess the publication bias of studies included in the analysis. Within-group heterogeneity was assessed using I^2-statistic with ≥50% threshold indicating substantial heterogeneity. The Forest plots were used to present the magnitude and direction of the overall effect size relative to the line of no effect. Estimation was done at a 5% significant level.

SPSS version 22.0 was used to conduct all analyses.

Data Synthesis

Further data management, quality control and required analysis were carried out based on the recommended guidance [51]. Overall, four studies were considered to address the mental health-related outcomes in PLWMI. The outcomes considered included "Depression, Recovery, Self-Efficacy, and Social Withdrawal". Additionally, three studies were considered to address stigma-related outcomes in PLWMI, with "stigma resistance" being the only outcome evaluated. Furthermore, in addressing the mental health-related outcomes in HCPs, three studies that met eligibility criteria were available for the analysis and one outcome, "anger" was evaluated for this analysis. Finally, addressing the stigma-related outcomes in HCPs necessitated the evaluation of three studies that met the criteria for two outcomes, "perception of danger" and social distance".

Results

Study Selection

The database search identified a total of 331 potentially relevant studies which were imputed into Covidence for the removal of duplicates. At this stage, a total of 31 duplicates were removed, leaving a total of 300 studies for screening based on the titles and abstracts on Covidence. The titles and abstracts screening led to the removal of a further 283 articles, leaving a total of 17 articles for full-text reading. At this stage, two reviewers read the full text of the 17 articles to determine their adherence to the eligibility criteria in the current review. This led to an exclusion of 10 articles, leaving a total of 7 articles for quality assessment in this review. Reasons for exclusion included the identification of 2 studies as study protocols, 2 studies did not utilise photovoice-based interventions, and 6 studies were not randomised controlled trials. Fig 1 illustrates the study selection process for the current review. See Table 1 for the characteristics of the seven included studies.

Fig 1: PRISMA flow chart.

Fig 1.tif

Table 1: Characteristics of included studies.

Study / Setting / Country	Population	Design	Intervention	Comparison	Outcomes	Outcome measures	Gender / Age	Sample size	Results
1. Zhang et al 2023 Hong Kong, China	PLWMI	Mixed Methods (quantita-tive analysis designed as an RCT).	Mindfulness-Based Family Psychoeducation Intervention with a Photovoice component	Family Psychoedu-cation (FPE)	1) recovery	1) MHRM		18 Young adults in recovery (YAIR)	Recovery level of YAIR increased after their caregivers participated in MBFPE
2. Flanagan et al 2016 USA	HCPs	RCT	1hr photovoice performance by PLWMI	No intervention	1) anger 2) recovery 3) social distance	1) Attribution Q. 2) RKI 3) SDS	93% female / mean age ± SD 49.36± 10.5	27 HCPs	significantly decreased negative stereotypes, attribution of dangerousness, fear, , desire to segregate people from the community,

Study / Setting / Country	Population	Design	Intervention	Comparison	Outcomes	Outcome measures	Gender / Age	Sample size	Results
									and desire for avoidance.
3. Russinova et al 2018 USA	PLWMI	RCT	Vocational Empowerment Photovoice (VEP)	No intervention	1) social withdrawal<br>2) stigma resistance	ISMI	Interve-ntion group – 66.7% female; Control – 55.6% female	51 individuals with psychiatric disabilities	- significantly higher rate of engagement in employment services<br>- increased work hope, self-efficacy and sense of vocational identity
4. Russinova et al 2023 USA	PLWMI	RCT	Bridging Community Gaps Photovoice (BCGP)" program	Services as usual	1) depression<br>2) recovery<br>3) self-efficacy<br>4) social withdrawal<br>5) stigma resistance	1) BASIS-24<br>2) PGRS<br>3) ICP-MH<br>4&5) ISMI	Incterv ention group – 60.6% female; Control – 61.5% female	185 participants	- increased community engagement<br>- reduced self-stigma<br>- significant impact on self-efficacy

Study / Setting / Country	Population	Design	Intervention	Comparison	Outcomes	Outcome measures	Gender / Age	Sample size	Results
5. Russinova et al 2014 USA	PLWMI	RCT	peer-run photography-based intervention	No intervention	1) Depression 2) Recovery 3) Self-efficacy 4) Social withdrawal 5) Stigma resistance .	1) Depression scale 2) PGRS 3) GPSES 4&5) ISMI	68% >40yrs; 68% female	82 PLWMI	significantly reduced self-stigma, greater , and perceived recovery and growth. no differences between groups about depression, self-efficacy
6. Kohrt et al 2021 Nepal	HCPs	Pilot Cluster RCT	RESHAPE training	Training as usual (mhGAP-IG)	1) Perception of danger. 2) Social distance	1) SDS 2) IAT	85% male / mean age ± SD 36.2 ± 8.8 years -	88 PCPs	reduced stigma compared with standarad training
7. Tippin and Maranzan 2019	HCPs	RCT	Online anti-stigma photovoice-based video	Control video	1) anger 2) social distance	1) Atrribution Q 2) SDS	Female: 73.9%; Male:26.1%	n = 303	efficacious in reducing mental illness stigma; reduced fear and anger

Study / Setting / Country	Population	Design	Intervention	Comparison	Outcomes	Outcome measures	Gender / Age	Sample size	Results
Canada							Mean age - 21.44; SD (5.0)		toward PLWMI, decreased perceptions of dangerousnes s, and desired social distance.

KEY

HCPs – Healthcare Practitioners

MBFPE - Mindfulness-based Family Psychoeducation

PLWMI - People living with mental illness

YAIR - Young adults in recovery

BASIS-24 – Behaviour and Symptom Identification Scale

GPSES – Generalized Perceived Self-efficacy Scale

IAT – Implicit Attitude Test

ICP – MH – Inventory of Community Participation – Mental Health

ISMI – Internalized Stigma of Mental Illness

MHRM – Mental Health Recovery Measure

PGRS – Personal Growth and Recovery Scale

- RKI – Recovery Knowledge Inventory
- SDS – Social Distance Scale

Quality Assessment results

As earlier indicated, the seven included studies were assessed for quality by utilising the EPHPP tool. The EPHPP tool on Covidence facilitated the extraction of relevant data for quality assessment. Regarding selection bias, two studies were rated strong, while five studies were rated moderate. Regarding the study design, all included studies were rated strong which is a characteristic shortcoming of the EPHPP tool as RCTs are automatically assigned a strong rating for this domain.

Regarding the influence of confounders on the outcomes, two studies received a strong rating which indicated that there were no differences in baseline measures between intervention and control groups, while five studies were rated weak due to differences between groups at baseline.

Only one study received a strong rating for blinding which indicated that the research question was concealed from participants and that outcome assessors were not aware of the intervention status of participants, while the remaining studies received a moderate rating for this category.

Additionally, all studies utilised valid and reliable tools for data collection, with full details provided on how the tools were validated before data collection leading to a strong rating for all included studies in this domain.

Regarding withdrawals and dropouts, two studies were rated strong because they reported the withdrawals fully, including reasons for loss of participants and above 80% of study participants completed the studies. Additionally, 3 studies received a moderate rating due to recording a 75% study completion rate among study participants. Furthermore, 2 studies received a weak rating in this category. One of the studies recorded less than 60% study completion rate, in contrast to the final study that did not report on withdrawals. Overall, only one study was assessed as strong indicating a high quality, five studies were assessed as moderate indicating moderate quality, while one study was assessed as weak indicating a weak quality.

Table 2 details the methodological quality assessment conducted based on the EPHPP tool.

Table 2: EPHPP tool Quality assessment results

		Khort et al 2023	Flanagan *et al* 2016	Zhang *et al* 2023	Russinova *et al* 2014	Russinova *et al* 2018	Russinova *et al* 2023	Tippin and Maranzan 2019
**A. SELECTION BIAS**								
Q1	Representative of the target population?	VL	SL	SL	SL	SL	VL	SL
Q2	Percentage of selected that agreed to participate?	100%	CT	CT	CT	CT	80-100%	CT
Section Rating		Strong	Mod	Mod	Mod	Mod	Strong	Mod
**B. STUDY DESIGN**								
Q1	Described as RCT?	Yes	Yes	Yes	Yes	Yes	Yes	Yes
Q2	Randomization method described?	Yes	No	Yes	No	No	No	No
Q3	Randomization method appropriate?	Yes	N/A	Yes	No	No	No	No
Section Rating		Strong	Strong	Strong	Strong	Strong	Strong	Strong
**C. CONFOUNDERS**								
Q1	Important differences between groups before intervention?	No	CT	CT	No	Yes	CT	CT
Q2	If Yes, percentage of confounders controlled?	N/A	N/A	N/A	N/A	CT	CT	CT
Section Rating		Strong	Weak	Weak	Strong	Weak	Weak	Weak
**D. BLINDING**								
Q1	Outcome assessors aware of intervention status of participants?	Yes	Yes	CT	Yes	Yes	CT	No
Q2	Study participants aware of the research question?	No	No	No	CT	CT	CT	No
Section Rating		Mod	Mod	Mod	Mod	Mod	Mod	Strong
**E. DATA COLLECTION METHODS**								
Q1	Data collection tool valid?	Yes	Yes	Yes	Yes	Yes	Yes	Yes
Q2	Data collection tool reliable?	Yes	Yes	Yes	Yes	Yes	Yes	Yes
Section Rating		Strong	Strong	Strong	Strong	Strong	Strong	Strong
**F. WITHDRAWALS**								
Q1	Withdrawals and dropouts reported?	Yes	No	Yes	Yes	Yes	Yes	Yes
Q2	Indicated the percentage of participants that completed the study?	93%	N/A	N/A	75%	75%	90%	<60%
Section Rating		Strong	Weak	Mod	Mod	Mod	Strong	Weak
**Global Rating For Each Study**		**STRONG**	**WEAK**	**MOD**	**MOD**	**MOD**	**MOD**	**WEAK**

KEY: VL – Very Likely; SL – Somewhat Likely; CT – Can't Tell; MOD – Moderate; N/A – Not Available

Adapted from: Effective Public Healthcare Panacea Project [2009] *Quality Assessment Tool for Quantitative Studies*, *Effective Public Healthcare Panacea Project*. Available at: https://www.ephpp.ca/quality-assessment-tool-for-quantitative-studies

Effect of photovoice on the mental health-related outcomes in PLWMI

Depression

Two papers provided data on the effect of photovoice on depression as an outcome of mental illness. The overall effect size was -0.23 (95% CI, - 0.45 to -0.02) demonstrating a 23% reduction in depression following the use of photovoice. The forest plot of the effect size of photovoice on depression is presented in Fig 2A.

Recovery

Three papers provided data on the effect of photovoice on recovery as an outcome of mental illness. The overall effect size was 0.19 (95% CI, -0.02 to 0.41) demonstrating a 19% chance of recovery from mental illness when photovoice is used. The forest plot of the effect size of photovoice on recovery is presented in Fig 2B.

Self-Efficacy

Two papers provided data on the effect of photovoice on self-efficacy as an outcome of mental illness. The overall effect was 0.22 (95% CI, 0.00 to 0.44) demonstrating a 22% increase in self-efficacy after photovoice was used. The forest plot of the effect size of photovoice on self-efficacy is presented in Fig 2C.

Social Withdrawal

Three papers provided data on the effect of photovoice on self-withdrawal as an outcome of mental illness. The overall effect size was -0.2 (95% CI, -0.41 to 0.00) demonstrating a 20% reduction in social withdrawal after photovoice was used. The forest plot of the effect size of photovoice on anger is presented in Fig 2D.

Fig 2: Forest Plot of Effect Size of photovoice on mental health-related outcomes of PLWMI.

fig 2 A to D.tif (A) Depression, (B) Recovery, (C) Self-Efficacy (D) Social withdrawal.

Effect of photovoice on the stigma-related outcomes in PLWMI

Stigma Resistance

Three papers provided data on the effect of photovoice on stigma resistance as an outcome of mental illness. The overall effect size was -0.13 (95% CI, -0.36 to 0.10) demonstrating a 13% reduction in stigma resistance after photovoice was used. The forest plot of the effect size of photovoice on stigma resistance is presented in Fig 3.

Fig 3: Forest Plot of Effect Size of photovoice on stigma-related outcomes of PLWMI.

fig 3.tif

Effect of photovoice on the mental health-related outcomes in HCPs

Anger

Two papers provided data on the effect of photovoice on anger as an outcome of mental illness. The overall effect size of photovoice on anger was -1.05 (95% CI, - 2.80 to 0.69). This demonstrates that photovoice reduced anger in study participants by 105% following the use of photovoice. The forest plot of the effect size of photovoice on anger is presented in Fig 4.

Fig 4: Forest Plot of Effect Size of photovoice on mental health-related outcomes in HCPs.

Fig 4.tif

Effect of photovoice on the stigma-related outcomes in HCPs

Social distance

Three papers were utilised to extract data on the photovoice-generated effect size for social distancing in HCPs. Overall, the effect size of photovoice on social distance in HCPs was -1.41 (95% CI, -3.02 to 1.03). This demonstrates that photovoice reduced social distance in study participants by 141% following use of photovoice. The forest plot of the effect size of photovoice on social distance is presented in Fig 5A.

Perception of Danger

Two papers were utilised to extract data on the photovoice-generated effect size for social distancing in HCPs. Photovoice had a -1.80 (95% CI, -5.04 to 1.44) effect size on the perception of danger in HCPs. This demonstrates that photovoice reduced the perception of danger in study participants by 180% following the use of photovoice. The forest plot of the effect size of photovoice on perception of danger is presented in Fig 5B. Table 3 details the effect sizes calculated from the available raw data for all outcomes.

Fig 5: Forest Plot of Effect Size of photovoice on stigma-related outcomes in HCPs.

Fig 5 A to B.tif (A) Social Distance (B) Perception of Danger.

Table 3: **Effect Size of photovoice on mental health and stigma-related outcomes in both PLWMI and HCPs.**

Outcome	Study ID	Cohen's d	Std. Error	95% C I		Heterogeneity
				Lower	Upper	
**Mental Health related outcomes in PLWMI**						
Depression	Russionova et al 2014	-0.33	0.16	-0.63	0.03	Tau-squared =0.00<br>H-squared=1.00<br>I-squared= 0.00
	Russinova et al 2023	-0.13	0.16	-0.45	0.18	
	Overall	-0.23	0.11	-0.45	-0.02	
Recovery	Zhang et al 2023	0.88	0.52	-0.15	1.90	Tau-squared=0.00<br>H-squared = 1.00<br>I-squared= 0.00
	Russinova et al 2014	0.16	0.15	-0.14	0.46	
	Russinova et al 2023	0.17	0.16	-0.15	0.48	
	Overall	0.19	0.11	-0.02	0.41	
Self-Efficacy	Russionova et al 2014	0.27	0.15	-0.04	0.57	Tau-squared=0.00<br>H-squared = 1.00<br>I-squared= 0.00
	Russinova et al 2023	0.18	0.16	-0.14	0.49	
	Overall	0.22	0.11	0.00	0.44	
Social Withdrawal	Russionova et al 2014	-0.31	0.29	-0.88	0.27	Tau-squared=0.00<br>H-squared = 1.00<br>I-squared= 0.00
	Russionova et al 2018	-0.17	0.15	-0.47	0.13	
	Russinova et al 2023	-0.21	0.16	-0.52	0.11	
	Overall	-0.20	0.10	-0.41	0.00	
**Mental Health related Outcomes in HCPs**						
Anger	Flanagan et al 2016	-2.00	0.46	-2.91	-1.09	Tau-squared=1.48

Outcome	Study ID	Cohen's d	Std. Error	95% C I		Heterogeneity
				Lower	Upper	
	Tippin and Maranzan 2019	-0.22	0.11	-0.44	0.01	H-squared = 14.00<br>I-squared= 0.93
	Overall	-1.05	0.89	-2.80	0.69	
**Stigma-related outcomes in PLWMI**						
Stigma Resistance	Russionova et al 2014	-0.16	0.29	-0.73	0.41	Tau-squared=0.01<br>H-squared = 1.20<br>I-squared= 0.17
	Russionova et al 2018	0.02	0.15	-0.28	0.32	
	Russinova et al 2023	-0.28	0.16	-0.60	0.02	
	Overall	-0.13	0.12	-0.36	0.10	
**Stigma-related outcomes in HCPs**						
Social Distance	Flanagan et al 2016	-3.20	0.58	-4.34	-2.06	Tau-squared=1.90<br>H-squared = 33.86<br>I-squared= 0.97
	Tippin and Maranzan 2019	-0.86	0.22	-1.32	-0.41	
	Kohrt et al 2021	-0.42	0.11	-0.65	-0.20	
	Overall	-1.41	0.82	-3.02	0.20	
Perception of danger	Kohrt et al 2021	-3.5	0.60	-4.68	-2.32	Tau-squared=5.27<br>H-squared = 26.64<br>I-squared= 0.96
	Flanagan et al 2016	-0.19	0.22	-0.63	0.25	
	Overall	-1.80	1.65	-5.04	1.44	

Discussion

The results of this meta-analysis suggest that photovoice is an effective tool for reducing negative mental health and stigma outcomes in PLWMI and improving positive outcomes. Conceptually, this is interlinked with synthesised outcome measures, including recovery, self-efficacy,

and stigma resistance. However, debates among researchers on the notion of recovery continue [52,53], with two prominent perspectives: personal and functional recovery [54].

Traditional mental health interventions such as clinical staging, a deficit-based strategy that aims to mitigate outcomes of mental illness for achieving functional recovery have been effective in reducing clinical symptoms [55,56]. However, such interventions have been criticised for being reductionistic, and not focusing on the perspective of the PLWMI regarding the meaning of recovery [52]. In contrast, photovoice represents a personal recovery-oriented approach that emphasises strength-based methods [57]. Strength-based approaches focus on increasing the resilience in PLWMI through the key characteristics of personal recovery including acceptance, hope, personal distinctiveness, agency/autonomy, social cohesion, and engagement [58].

Similarly, the efficacy of adopting participatory research methods such as photovoice by PLWMI within recovery-oriented mental health has been advocated [59-61]. This is facilitated by the sense of functionality photovoice provides, which promotes self-esteem and efficacy, as well as social dimensions of recovery by reducing isolation, fostering community engagement, and increasing the individual's sense of self-worth [59]. This can be seen in a quasi-experimental study done with participants from a psychosocial rehabilitation centre which highlights that photovoice facilitates recovery-related outcomes such as empowerment, positive sense of identity, and community integration, and therefore suggests its incorporation into recovery-oriented services of PLWMI [62]. Furthermore, such participatory research enables in-depth communication with the healthcare providers, wherein strength-based experiences beyond living with mental illness can be expressed, such as their interests and contributions to society, which has been shown to significantly reduce healthcare provider stigma, and inadvertently increase quality of care, and chance of recovery [63].

In contrast, stigma resistance, the ability to challenge mental health stigma, stereotypes, and public prejudice at the personal, peer, and societal level [64], was decreased in this study following the

administration of photovoice intervention. Perhaps, this reduction in stigma resistance is due to the short duration of studies analysed for this category because the photovoice intervention increases participants' sensitivity to stigmatising attitudes in the short term [65]. The study recommended that researchers should be aware of the initial phase of stereotype agreement to design efficacious interventions intended to mitigate self-stigma. However, further research revealed that Photovoice fosters empowerment and self-identity, which posed a protective barrier against internalising stigma and discrimination [11]. Likewise, its ability to build resilience, which is effective in mitigating internalised and self-stigma in vulnerable populations has been evidenced [66-69]. Consequently, increased resilience leads to an overall sense of empowerment for the PLWMI ensuring increased confidence, fostering engagement and connectedness within communities [57].

Also, self-efficacy, which can be described as the ability of PLWMI to cope and thrive with their mental illness [70], was shown to be enhanced through the influence of photo-elicitation programs, in building sustained sense of self-worth within themselves and engagement with their community [71,72]. This is evident in a community-based participatory action research with participants from a community mental health institution, where they expounded on the inherent challenges in executing activities of daily living, especially regarding healthy lifestyle choices, due to the inability of set measures to adequately cater to their needs [73]. The participants elaborated that photovoice availed an opportunity to be consulted for what they needed to lead healthier lives and facilitated co-creation of strategies with their mental health nurses which enabled successful execution of healthier behaviours [73]. This offers insight to the challenges of living with mental illness and empowers PLWMI to be advocates for addressing their unmet needs, which promotes a sense of purpose and achievement [70].

Furthermore, exposure to photovoice intervention significantly reduced depression among PLWMI in the current study. This relates to the findings of a qualitative study among rural adolescents [15/17 years old] with depression and suicidal tendencies in northeast America who experienced higher

sense of self-worth and empowerment after photovoice intervention [74]. Likewise, the current meta-analysis aligns with the finding of a study in Nepal among twenty-seven 49-year-old rural women with self-reported depression, as measured by the Beck Depression Inventory (BDI), which was found to be significantly reduced after exposure to photovoice intervention [75]. The current meta-analysis also highlights the potential benefits of photovoice intervention in reducing social withdrawal in PLWMI. This finding is consistent with a previous qualitative study conducted among individuals with mental health issues in a mental health club in Hawaii, which identified photovoice as a helpful tool in fostering self-appraisal and inner empowerment, leading to improved social support [76].

Concomitantly, the results also suggest that photovoice is effective for reducing social distancing, perception of danger, and anger from healthcare providers towards PLWMI. The visual narratives created can serve as a powerful medium for reducing stereotypes and foster empathy among the HCPs [77]. Importantly, social distancing from healthcare providers towards PLWMI can intensify the stigma experienced by PLWMI [78]. Two perspectives worth considering are the contact-based and humanistic approaches.

One of Allport's seminal criteria for the nature of contact in PLWMI is the perception of equal status between the contact and the receiver [57,79]. Traditional contact-based approaches however disregard the inherent inequality in social status between healthcare providers and PLWMI [57], explaining why this form of contact is ineffective or produces only short-term effects [80,81]. Photovoice, however, focuses on humanistic approaches, emphasizing empathy, dignity, and respect towards the challenges experienced by PLWMI [82]. In this context, health professionals can relate with the experiences of PLWMI, explaining its remarkable effect on mental health and stigma-related outcomes such as social distancing, anger, and perception of danger within this population [82]. Consequently, the balance of power shifts from a feeling of "power over" inherent in healthcare professionals to "power within" in PLWMI [83].

Implications for policy and practice and limitations of the study

This systematic review has explored the effectiveness of photovoice on mental health outcomes in PLWMI and their healthcare providers. Building from the findings, mental health services should consider incorporating photovoice as a personal recovery-oriented tool as opposed to emphasis on traditional functional recovery, acknowledging its role in fostering empowerment and reducing stigma. Policies that encourage participatory research methods should be implemented, emphasising communication between PLWMI and healthcare providers. The adoption of these measures can contribute to a more holistic and individualized approach to mental health care.

Additionally, the current meta-analysis was limited based on heterogeneity due to the small number of studies exploring certain outcomes. Three outcomes, anger (0.93), perception of danger (0.96), and social distance (0.99) demonstrated high heterogeneity, which was attributed to the limited number of included studies in the meta-analysis for these outcomes [84]. While the reported statistic, I^2, provides an estimate, it does not eradicate the uncertainty that arises from having a limited number of studies in a meta-analysis [85]. Consequently, it is important to interpret the heterogeneity encountered in both outcomes with caution. Nonetheless, the majority of the included studies were of moderate quality, with one study of high quality, and two studies having low quality. Future RCTs should therefore consider exhausting all outcomes of mental illness, extending to outcomes not included in this meta-analysis. As mental healthcare continues to evolve, further research and the successful implementation of these strategies are essential for promoting positive mental health outcomes for diverse populations.

Conclusion

Overall, it can be deduced that photovoice does have a significant effect on improving the mental health outcomes of PLWMI. Conversely, the findings suggest that photovoice reduces the

positive stigma-related outcomes in PLWMI, such as stigma resistance in the short term. Furthermore, photovoice significantly reduces the negative mental health and stigma-related outcomes in HCPs that have consequences for the mental health of PLWMI. Therefore, it can be concluded that photovoice improves the mental health outcomes of PLWMI and has a positive effect on the mental health and stigma-related behaviours of HCPs towards PLWMI. Therefore, the effect of photovoice on stigma resistance warrants further research.

Acknowledgements

The authors acknowledge See Me Scotland for providing funds and logistics support for this project.

Funding

This work was supported by See Me Scotland (URL: https://www.seemescotland.org/). There was no specific grant number associated with the grant. JW was the specific recipient of the award. CN, WO, and CS received compensation. "The funders had no role in study design, data collection and analysis, decision to publish, or preparation of the manuscript."

Competing interests

The authors have read the journal's policy and hereby declare no financial or non-financial conflicts of interest.

Author contributions

AA and JW were involved in the conception, design, project management, and supervision of the review. WO, CS, and CN designed and conducted the searches and screened retrieved records for inclusion. WO, CS, AA and AA extracted data from primary studies included for meta-analyses,

checked by JW and UA. WO and CS performed quality assessment, with differences to be reconciled by AA, AA and UA. AA, AA and UA conducted the meta-analyses, with AA validating the final results of the meta-analyses. UA drafted the manuscript, after which WO, CS, JW, AA and AA contributed to commenting on drafts and suggesting any revisions. All authors approved the final version and verified their contributions.

References

1. Han CS, Oliffe JL. Photovoice in mental illness research: A review and recommendations. Health Lond Engl 1997 [Internet]. 2016 Mar [cited 2023 May 19];20[2]:110–26. Available from: https://doi.org/10.1177/1363459314567790

2. Leyva-Moral JM, Gómez-Ibáñez R, San Rafael S, Guevara-Vásquez G, Aguayo-González M. Nursing students' satisfaction with narrative photography as a method to develop empathy towards people with Hiv: A mixed-design study. Nurse Educ Today [Internet]. 2021 Jan 1 [cited 2024 Jan 21];96:104646. Available from: https://doi.org/10.1016/j.nedt.2020.104646

3. Husin SS, Ab Rahman AA, Mukhtar D. The symbolic interactionism theory: a systematic literature review of current research. Int J Mod Trends Soc Sci [Internet]. 2021 Sep 15 [cited 2024 Jan 21];4[17]:113–26. Available from: DOI: 10.35631/IJMTSS.417010

4. Halvorsrud K, Eylem O, Mooney R, Haarmans M, Bhui K. Identifying evidence of the effectiveness of photovoice: a systematic review and meta-analysis of the international healthcare literature. J Public Health [Internet]. 2022 Sep 1 [cited 2023 May 24];44[3]:704–12. Available from: https://doi.org/10.1093/pubmed/fdab074

5. Elraz H. Identity, mental health and work: How employees with mental health conditions recount stigma and the pejorative discourse of mental illness. Hum Relat [Internet]. 2018 May 1 [cited 2024 Jan 21];71[5]:722–41. Available from: https://doi.org/10.1177/0018726717716752

6. Baron EC, Rathod SD, Hanlon C, Prince M, Fedaku A, Kigozi F, et al. Impact of district mental health care plans on symptom severity and functioning of patients with priority mental health conditions: the Programme for Improving Mental Health Care [PRIME] cohort protocol. BMC Psychiatry [Internet]. 2018 Mar 6 [cited 2024 Jan 21];18[1]:61. Available from: https://doi.org/10.1186/s12888-018-1642-x

7. Sestito SF, Rodriguez KL, Saba SK, Conley JW, Mitchell MA, Gordon AJ. Homeless Veterans' Experiences with Substance Use, Recovery, and Treatment through Photo Elicitation. Subst Abuse [Internet]. 2017 Oct 1 [cited 2024 Jan 21];38[4]:422–31. Available from: https://doi.org/10.1080/08897077.2017.1356422

8. True G, Rigg KK, Butler A. Understanding Barriers to Mental Health Care for Recent War Veterans Through Photovoice. Qual Health Res [Internet]. 2015 Oct [cited 2023 Nov 5];25[10]:1443–55. Available from: http://journals.sagepub.com/doi/10.1177/1049732314562894

9. Kopera M, Suszek H, Bonar E, Myszka M, Gmaj B, Ilgen M, et al. Evaluating Explicit and Implicit Stigma of Mental Illness in Mental Health Professionals and Medical Students. Community Ment Health J. 2015; 51(5):628-634 Available from: https://doi.org/10.1007/s10597-014-9796-6 doi:10.1007/s10597-014-9796-6.

10. Kohrt BA, Jordans MJD, Turner EL, Rai S, Gurung D, Dhakal M, et al. Collaboration With People With Lived Experience of Mental Illness to Reduce Stigma and Improve Primary Care Services: A Pilot Cluster Randomized Clinical Trial. JAMA Netw Open [Internet]. 2021 Nov 3 [cited 2023 Jun 2];4[11]:e2131475. Available from: https://doi.org/10.1001/jamanetworkopen.2021.31475

11. Mizock L, Russinova Z, DeCastro S. Recovery narrative photovoice: Feasibility of a writing and photography intervention for serious mental illnesses. Psychiatr Rehabil J [Internet]. 2015 Sep [cited 2023 Nov 5];38[3]:279–82. Available from: http://doi.apa.org/getdoi.cfm?doi=10.1037/prj0000111

12. Russinova Z, Gidugu V, Bloch P, Restrepo-Toro M, Rogers ES. Empowering individuals with psychiatric disabilities to work: Results of a randomized trial. Psychiatr Rehabil J. 2018;41:196–207. Available from: https://doi.org/10.1037/prj0000303

13. Becker K, Reiser M, Lambert S, Covello C. Photovoice: Conducting Community-Based Participatory Research and Advocacy in Mental Health. J Creat Ment Health [Internet]. 2014 Apr 3 [cited 2023 Apr 8];9[2]:188–209. Available from: https://doi.org/10.1080/15401383.2014.890088

14. Yanos PT, Lucksted A, Drapalski AL, Roe D, Lysaker P. Interventions targeting mental health self-stigma: A review and comparison. Psychiatr Rehabil J. 2015;38[2]:171–8. Available from: https://doi.org/10.1037/prj0000100

15. Nykiforuk CIJ. Engaging patients in research using photovoice methodology. CMAJ [Internet]. 2021 Jul 12 [cited 2023 Apr 6];193[27]:E1050–1. Available from: https://doi.org/10.1503/cmaj.210963

16. Creighton G, Oliffe JL, Ferlatte O, Bottorff J, Broom A, Jenkins EK. Photovoice Ethics: Critical Reflections From Men's Mental Health Research. Qual Health Res [Internet]. 2018 Feb [cited 2023 Jun 15];28[3]:446–55. Available from: DOI:10.1177/1049732317729137

17. Woda A, Haglund K, Belknap R, Cleek E. Photovoice: A Research Method and Intervention to Engage Older Adults. Journal of gerontological nursing . 2018 Jul 1, ; 44(7):43-49 Available from:https://www.ncbi.nlm.nih.gov/pubmed/29969139 doi:10.3928/00989134-20180614-07.

18. Call-Cummings M, Hauber-Özer M, Byers C, Mancuso GP. The power of/in Photovoice. International journal of research & method in education . 2019 Aug 8, ; 42(4):399-413 Available from:https://www.tandfonline.com/doi/abs/10.1080/1743727X.2018.1492536 doi:10.1080/1743727X.2018.1492536.

19. Christensen MC. Using Photovoice to Address Gender-Based Violence: A Qualitative Systematic Review. Trauma Violence Abuse . 2019 Oct ; 20(4):484-497 doi:10.1177/1524838017717746.

20. Wang C, Burris MA. Photovoice: Concept, Methodology, and Use for Participatory Needs Assessment. Health Educ Behav [Internet]. 1997 Jun [cited 2023 Apr 6];24[3]:369–87. Available from: http://journals.sagepub.com/doi/10.1177/109019819702400309

21. Carlson ED, Engebretson J, Chamberlain RM. Photovoice as a Social Process of Critical Consciousness. Qualitative health research . 2006 Jul ; 16(6):836-852 Available from:https://journals.sagepub.com/doi/full/10.1177/1049732306287525 doi:10.1177/1049732306287525.

22. Catalani C, Minkler M. Photovoice: A Review of the Literature in Health and Public Health. Health Education & Behavior . 2010 Jun 1, ; 37(3):424-451 Available from:https://www.jstor.org/stable/45056201 doi:10.1177/1090198109342084.

23. Liebenberg L. Thinking Critically About Photovoice. International journal of qualitative methods . 2018 Dec 1, ; 17(1):160940691875763 Available

from:https://journals.sagepub.com/doi/full/10.1177/1609406918757631 doi:10.1177/1609406918757631.

24. Hergenrather KC, Rhodes SD, Cowan CA, Bardhoshi G, Pula S. Photovoice as Community-Based Participatory Research: A Qualitative Review. American journal of health behavior . 2009 Nov 1, ; 33(6):686-698 Available from:https://www.ingentaconnect.com/content/png/ajhb/2009/00000033/00000006/art00006 doi:10.5993/AJHB.33.6.6.

25. Jacobs R. Investigating Patient Outcome Measures in Mental Health.: Centre for Health Economics, University of York; 2009.

26. Brohan E, Slade M, Clement S, Thornicroft G. Experiences of mental illness stigma, prejudice and discrimination: a review of measures. BMC Health Services Research . 2010; 10(1):80 Available from: https://doi.org/10.1186/1472-6963-10-80 doi:10.1186/1472-6963-10-80.

27. Uman LS. Systematic Reviews and Meta-Analyses. J Can Acad Child Adolesc Psychiatry [Internet]. 2011 Feb [cited 2023 Sep 27];20[1]:57–9. Available from: https://www.ncbi.nlm.nih.gov/pmc/articles/PMC3024725/

28. Tawfik GM, Dila KAS, Mohamed MYF, Tam DNH, Kien ND, Ahmed AM, et al. A step by step guide for conducting a systematic review and meta-analysis with simulation data. Trop Med Health [Internet]. 2019 Aug 1 [cited 2023 Sep 27];47[1]:46. Available from: https://doi.org/10.1186/s41182-019-0165-6

29. Mikolajewicz N, Komarova SV. Meta-Analytic Methodology for Basic Research: A Practical Guide. Front Physiol [Internet]. 2019 [cited 2023 Sep 27];10. Available from: https://doi.org/10.3389/fphys.2019.00203

30. Paul J, Barari M. Meta-analysis and traditional systematic literature reviews—What, why, when, where, and how? Psychol Mark [Internet]. 2022 [cited 2023 Sep 27];39[6]:1099–115. Available from: https://doi.org/10.1002/mar.21657

31. Lee YH. Strengths and Limitations of Meta-Analysis. Korean J Med [Internet]. 2019 Oct 1 [cited 2023 Sep 27];94[5]:391–5. Available from: https://doi.org/10.3904/kjm.2019.94.5.391

32. Haidich AB. Meta-analysis in medical research. Hippokratia [Internet]. 2010 Dec [cited 2023 Sep 27];14[Suppl 1]:29–37. Available from: https://www.ncbi.nlm.nih.gov/pmc/articles/PMC3049418/

33. Hariton E, Locascio JJ. Randomised controlled trials—the gold standard for effectiveness research. BJOG Int J Obstet Gynaecol [Internet]. 2018 Dec [cited 2023 Sep 28];125[13]:1716. Available from: https://doi.org/10.1111/1471-0528.15199

34. Clyne B, Boland F, Murphy N, Murphy E, Moriarty F, Barry A, et al. Quality, scope and reporting standards of randomised controlled trials in Irish Health Research: an observational study. Trials [Internet]. 2020 Jun 8 [cited 2023 Sep 28];21[1]:494. Available from: https://doi.org/10.1186/s13063-020-04396-x

35. Bearman M, Smith C, Carbone A, Slade S, Baik C, Hughes-Warrington M, et al. Systematic review methodology in higher education. High Educ Res Dev. 2012 Oct 1;31. Available from: https://doi.org/10.1080/07294360.2012.702735

36. Paez A. Gray literature: An important resource in systematic reviews. J Evid-Based Med [Internet]. 2017 [cited 2023 Jun 16];10[3]:233–40. Available from: https://onlinelibrary.wiley.com/doi/abs/10.1111/jebm.12266

37. Adams J, Hillier-Brown FC, Moore HJ, Lake AA, Araujo-Soares V, White M, et al. Searching and synthesising 'grey literature' and 'grey information' in public health: critical reflections on three case studies. Syst Rev [Internet]. 2016 Sep 29 [cited 2023 Jul 28];5[1]:164. Available from: https://doi.org/10.1186/s13643-016-0337-y

38. Almeida CPB de, Goulart BNG de. How to avoid bias in systematic reviews of observational studies. Rev CEFAC [Internet]. 2017 Aug [cited 2023 Jul 11];19:551–5. Available from: https://doi.org/10.1590/1982-021620171941117

39. Haddaway NR, Collins AM, Coughlin D, Kirk S. The Role of Google Scholar in Evidence Reviews and Its Applicability to Grey Literature Searching. PLOS ONE [Internet]. 2015 Sep 17 [cited 2023 Jun 13];10[9]:e0138237. Available from: https://doi.org/10.1371/journal.pone.0138237

40. Kellermeyer L, Harnke B, Knight S. Covidence and Rayyan. J Med Libr Assoc JMLA [Internet]. 2018 Oct [cited 2023 Dec 1];106[4]:580–3. Available from: https://doi.org/10.5195%2Fjmla.2018.513

41. Vu-Ngoc H, Elawady SS, Mehyar GM, Abdelhamid AH, Mattar OM, Halhouli O, et al. Quality of flow diagram in systematic review and/or meta-analysis. PLOS ONE [Internet]. 2018 Jun 27 [cited 2023 Jul 20];13[6]:e0195955. Available from: https://doi.org/10.1371/journal.pone.0195955

42. Page MJ, McKenzie JE, Bossuyt PM, Boutron I, Hoffmann TC, Mulrow CD, et al. The PRISMA 2020 statement: an updated guideline for reporting systematic reviews. BMJ [Internet]. 2021 Mar 29 [cited 2022 Dec 30];372:n71. Available from: https://doi.org/10.1136/bmj.n71

43. dos Santos WM, Secoli SR, Püschel VA de A. The Joanna Briggs Institute approach for systematic reviews. Rev Lat Am Enfermagem [Internet]. 2018 Nov 14 [cited 2023 Sep 29];26:e3074. Available from: https://doi.org/10.1590%2F1518-8345.2885.3074

44. Centre for Reviews and Dissemination. CRD's guidance for undertaking reviews in healthcare. 3. ed. York: York Publ. Services; 2009. 281 p. [Systematic reviews].

45. Buchter RB, Weise A, Pieper D. Development, testing and use of data extraction forms in systematic reviews: a review of methodological guidance. BMC Med Res Methodol [Internet]. 2020 Oct 19 [cited 2023 Jul 20];20[1]:259. Available from: https://doi.org/10.1186/s12874-020-01143-3

46. Bown MJ, Sutton AJ. Quality Control in Systematic Reviews and Meta-analyses. Eur J Vasc Endovasc Surg [Internet]. 2010 Nov 1 [cited 2023 Jul 29];40[5]:669–77. Available from: https://doi.org/10.1016/j.ejvs.2010.07.011

47. Harrer M, Cuijpers P, Furukawa TA, Ebert DD. Doing meta-analysis with R.First edition ed. Boca Raton ; London ; New York: CRC Press; 2022.

48. Effective Public Healthcare Panacea Project. Effective Public Healthcare Panacea Project. 2009 [cited 2023 Dec 1]. Quality Assessment Tool for Quantitative Studies. Available from: https://www.ephpp.ca/quality-assessment-tool-for-quantitative-studies/

49. Meyvis T, Van Osselaer SMJ. Increasing the Power of Your Study by Increasing the Effect Size. J Consum Res [Internet]. 2018 Feb 1 [cited 2024 Jan 21];44[5]:1157–73. Available from: https://doi.org/10.1093/jcr/ucx110

50. Goulet-Pelletier J, Cousineau D. A review of effect sizes and their confidence intervals, part I: the Cohen's d family. The Quantitative Methods for Psychology . 2018 Dec 1, ; 14(4):242-265 Available from:https://doi.org/10.20982/tqmp.14.4.p242 doi:10.20982/tqmp.14.4.p242.

51. Ebert MH Pim Cuijpers, Toshi Furukawa, David. Doing Meta-Analysis with R: A Hands-On Guide. New York: Chapman and Hall/CRC; 2021. 500 p.

52. Treichler EBH, Li F, O'Hare M, Evans EA, Johnson JR, Spaulding WD. Psychosocial and functional contributors to personal recovery in serious mental illness. Journal of Mental Health . 2019; 28[4]:427-435 Available from: https://doi.org/10.1080/09638237.2018.1521932

53. Jahn DR, Leith J, Muralidharan A, Brown CH, Drapalski AL, Hack S, et al. The Influence of Experiences of Stigma on Recovery: Mediating Roles of Internalized Stigma, Self-Esteem, and Self-Efficacy. Psychiatric rehabilitation journal. 2020 Jun 1, ; 43[2]:97-105 Available from: https://psycnet.apa.org/doi/10.1037/prj0000377

54. Slade M, Amering M, Farkas M, Hamilton B, O'Hagan M, Panther G, et al. Uses and abuses of recovery: implementing recovery-oriented practices in mental health systems. World Psychiatry [Internet]. 2014 [cited 2024 Jan 21];13[1]:12–20. Available from: https://onlinelibrary.wiley.com/doi/abs/10.1002/wps.20084

55. Capon W, Hickie IB, Varidel M, Prodan A, Crouse JJ, Carpenter JS, et al. Clinical staging and the differential risks for clinical and functional outcomes in young people presenting for youth mental health care. BMC Medicine . 2022; 20[1]:479 Available from:https://doi.org/10.1186/s12916-022-02666-w

56. Hickie IB, Scott EM, Cross SP, Iorfino F, Davenport TA, Guastella AJ, et al. Right care, first time: a highly personalised and measurement-based care model to manage youth mental health. Med J Aust . 2019; 211:S3-S46 Available from:https://doi.org/10.5694/mja2.50383

57. Ungar T, Knaak S, Szeto AC. Theoretical and Practical Considerations for Combating Mental Illness Stigma in Health Care. Community Ment Health J [Internet]. 2016 Apr 1 [cited 2024 Jan 21];52[3]:262–71. Available from: https://doi.org/10.1007/s10597-015-9910-4

58. Cripps L, Deyell Hood C. Recovery and Mental Health: Exploring the Basic Characteristics of Living Well with Mental Illness. Ther Recreation J [Internet]. 2020 Jun 2 [cited 2024 Jan 21];54[2]. Available from: https://doi.org/10.18666/TRJ-2020-V54-I2-9948

59. Cabassa LJ, Nicasio A, Whitley R. Picturing Recovery: A Photovoice Exploration of Recovery Dimensions Among People With Serious Mental Illness. Psychiatr Serv Wash DC [Internet]. 2013 Sep 1 [cited 2023 May 19];64[9]:10.1176/appi.ps.201200503. Available from: https://doi.org/10.1176%2Fappi.ps.201200503

60. Vansteenkiste T, Morrens M, Westerhof GJ. Images of Recovery: A PhotoVoice Study on Visual Narratives of Personal Recovery in Persons with Serious Mental Illness. Community Ment Health J [Internet]. 2021 Aug [cited 2023 May 24];57[6]:1151–63. Available from: https://doi.org/10.1007/s10597-020-00746-w

61. Flanagan EH, Buck T, Gamble A, Hunter C, Sewell I, Davidson L. 'Recovery Speaks': A Photovoice Intervention to Reduce Stigma Among Primary Care Providers. Psychiatr Serv. 2016 May;67[5]:566–9. Available from: https://doi.org/10.1176/appi.ps.201500049

62. Mizock L, Russinova Z, DeCastro S. Recovery Narrative Photovoice: Feasibility of a Writing and Photography Intervention for Serious Mental Illnesses. Psychiatr Rehabil J . 2015; 38[3]:279-282 doi:10.1037/prj0000111

63. Boyd Ritsher J, Otilingam PG, Grajales M. Internalized stigma of mental illness: psychometric properties of a new measure. Psychiatry Res [Internet]. 2003 Nov 1 [cited 2024 Jan 21];121[1]:31–49. Available from: https://doi.org/10.1016/j.psychres.2003.08.008

64. Thoits PA. Resisting the Stigma of Mental Illness. Soc Psychol Q [Internet]. 2011 Mar 1 [cited 2024 Jan 21];74[1]:6–28. Available from: https://doi.org/10.1177/0190272511398019

65. CORRIGAN PW, WATSON AC, BARR L. The Self–Stigma of Mental Illness: Implications for Self–Esteem and Self–Efficacy. Journal of Social and Clinical Psychology . 2006 Oct 1, ; 25(8):875-884 Available from:https://search.proquest.com/docview/224857864 doi:10.1521/jscp.2006.25.8.875.

66. Adeboye A, Ross MW, Wilkerson MJ, Springer A, Ahaneku H, Yusuf RA, et al. Resilience Factors as a Buffer against the Effects of Syndemic Conditions on HIV Risk and Infection among Tanzanian MSM. Journal of Health Education Research & Development . 2017; 5[3] Available from: https://doi.org/10.4172/2380-5439.1000232.

67. Dow DE, Mmbaga BT, Turner EL, Gallis JA, Tabb ZJ, Cunningham CK, et al. Building resilience: a mental health intervention for Tanzanian youth living with HIV. AIDS Care . 2018; 30:12-20 Available from:https://doi.org/10.1080/09540121.2018.1527008

68. Macpherson H, Hart A, Heaver B. Building resilience through group visual arts activities: Findings from a scoping study with young people who experience mental health complexities and/or learning difficulties. Journal of Social Work . 2016; 16[5]:541-560 Available from:https://doi.org/10.1177/1468017315581772

69. McAllister M, Knight BA, Hasking P, Withyman C, Dawkins J. Building resilience in regional youth: Impacts of a universal mental health promotion programme. Int J Mental Health Nurs . 2018; 27[3]:1044-1054 Available from:https://doi.org/10.1111/inm.12412

70. Werremeyer AB, Aalgaard-Kelly G, Skoy E. Using Photovoice to explore patients' experiences with mental health medication: A pilot study. Ment Health Clin [Internet]. 2016 Jan 1 [cited 2023 May 24];6[3]:142–53. Available from: https://doi.org/10.9740/mhc.2016.05.142

71. Russinova Z, Rogers ES, Gagne C, Bloch P, Drake KM, Mueser KT. A randomized controlled trial of a peer-run antistigma photovoice intervention. Psychiatr Serv. 2014;65[2]:242–6. Available from: https://doi.org/10.1176/appi.ps.201200572

72. Russinova Z, Link to external site this link will open in a new window, Gidugu V, Rogers ES, Legere L, Bloch P. Fostering the community participation of individuals with psychiatric disabilities: Effectiveness of a new peer-led photovoice-based intervention. Psychiatr Rehabil J [Internet]. 2023 Mar 30 [cited 2023 May 30]; Available from: DOI:10.1037/prj0000540

73. Edmondson AJ, Borthwick R, Hughes E, Lucock M. Using photovoice to understand and improve healthy lifestyles of people diagnosed with serious mental illness. J Psychiatr Ment Health Nurs

[Internet]. 2022 [cited 2023 May 24];29[5]:676–87. Available from: https://onlinelibrary.wiley.com/doi/abs/10.1111/jpm.12863

74. Dempsey JS. Using Photovoice as a Community-Based Participatory Research Method to Identify Perceived Risk and Protective Factors for Depression in Rural Adolescents. Int J Nurs Clin Pract [Internet]. 2016 Aug 4 [cited 2023 May 24];3[1]. Available from: http://dx.doi.org/10.15344/2394-4978/2016/194

75. MacFarlane EK, Shakya R, Berry HL, Kohrt BA. Implications of participatory methods to address mental health needs associated with climate change: 'photovoice' in Nepal. BJPsych international . 2015 May ; 12[2]:33-35 Available from:https://dx.doi.org/10.1192/S2056474000000246

76. Agner J, Nakamura L, Botero A, Cha T, Kaukau TM. Can Photovoice foster the development of social support? Am J Community Psychol [Internet]. 2023 [cited 2024 Jan 21];71[3–4]:371–81. Available from: https://onlinelibrary.wiley.com/doi/abs/10.1002/ajcp.12649

77. Levy B, Celen-Demirtas S, Surguladze T, Sweeney KK. Stigma and Discrimination: A Socio-Cultural Etiology of Mental Illness. The Humanistic Psychologist. 2014; 42[2]:199-214 Available from: https://doi.org/10.1080/08873267.2014.893513

78. Munir K, Oner O, Kerala C, Rustamov I, Boztas H, Juszkiewicz K, et al. Social distance and stigma towards persons with serious mental illness among medical students in five European Central Asia countries. Psychiatry Res . 2022 Mar ; 309:114409 doi:10.1016/j.psychres.2022.114409.

79. Allport GW. The nature of prejudice. Cambridge, Mass: Addison-Wesley Pub. Co; 1954.

80. Carrara BS, Fernandes RHH, Bobbili SJ, Ventura CAA. Health care providers and people with mental illness: An integrative review on anti-stigma interventions. Int J Soc Psychiatry . 2021 Nov ; 67[7]:840-853 doi:10.1177/0020764020985891.

81. Jorm AF. Effect of Contact-Based Interventions on Stigma and Discrimination: A Critical Examination of the Evidence. PS . 2020; 71[7]:735-737 Available from: https://doi.org/10.1176/appi.ps.201900587

82. Coffey J. Images as 'potentials': Feminist new materialist orientations to photovoice. Qualitative Research . 2023; 23[4]:847-865 Available from:https://doi.org/10.1177/14687941211049334

83. Sweeney A, Filson B, Kennedy A, Collinson L, Gillard S. A paradigm shift: relationships in trauma-informed mental health services. BJPsych Adv [Internet]. 2018 Sep [cited 2024 Jan 21];24[5]:319–33. Available from: https://doi.org/10.1192/bja.2018.29

84. Stanley TD, Doucouliagos H, Ioannidis JPA. Beyond Random Effects: When Small-Study Findings Are More Heterogeneous. Adv Methods Pract Psychol Sci [Internet]. 2022 Oct 1 [cited 2024 Jan 21];5[4]:25152459221120427. Available from: https://doi.org/10.1177/25152459221120427

85. von Hippel PT. The heterogeneity statistic I2 can be biased in small meta-analyses. BMC Medical Research Methodology. 2015; 15[1]:35 Available from:https://doi.org/10.1186/s12874-015-0024-z

Supplementary material

S1. Fig. Full search history on all databases.

S1 Fig.docx

S1. Table. Data Extraction Form.

S1 Table.docx

S1. Appendix. Reference list of included studies.

S1 Appendix.docx

S2. Appendix. PRISMA 2020 Checklist.

S2 Appendix.docx

Figure
Click here to access/download;Figure;fig 2 A to D.tif

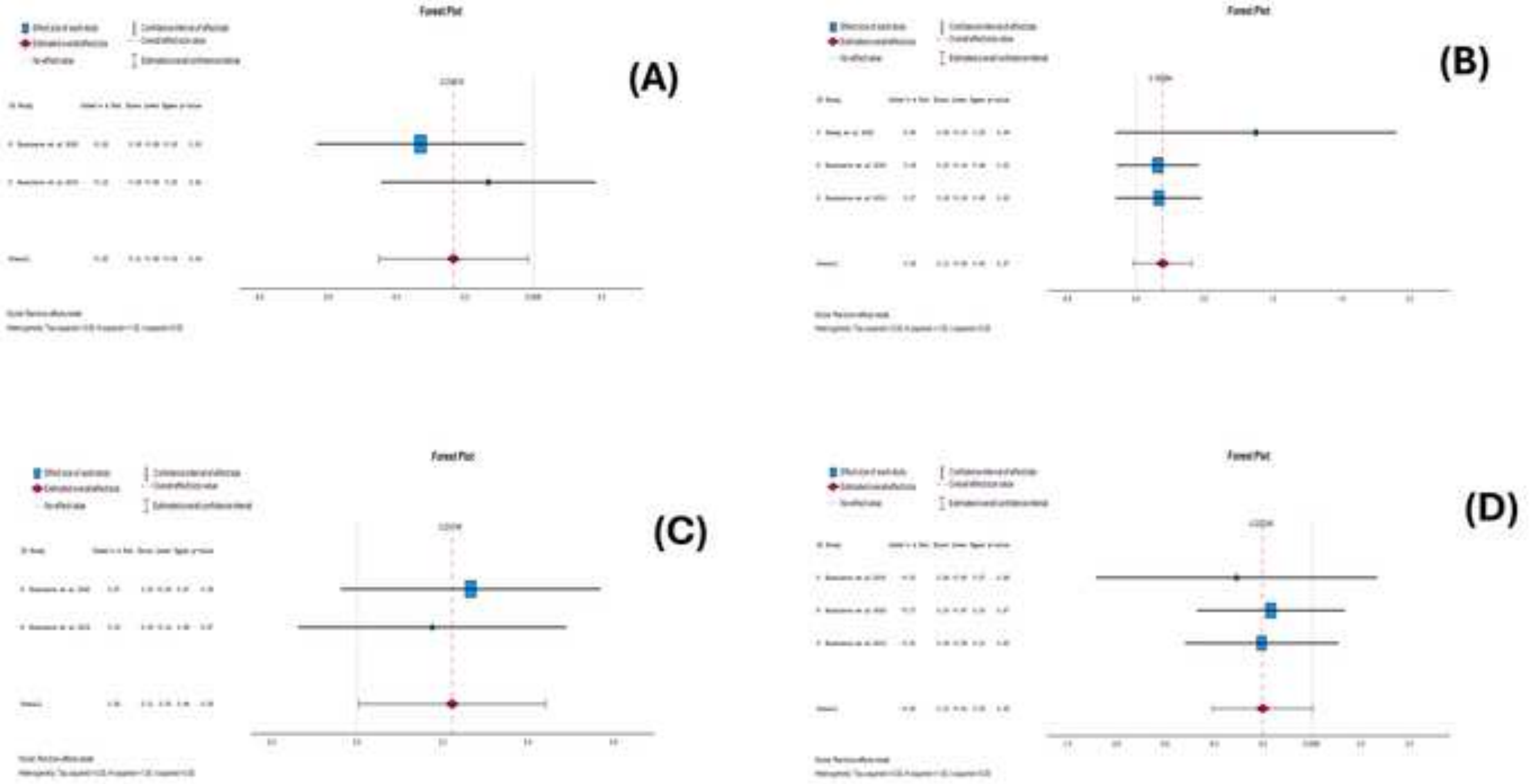

Figure

Click here to access/download;Figure;Fig 3.tif

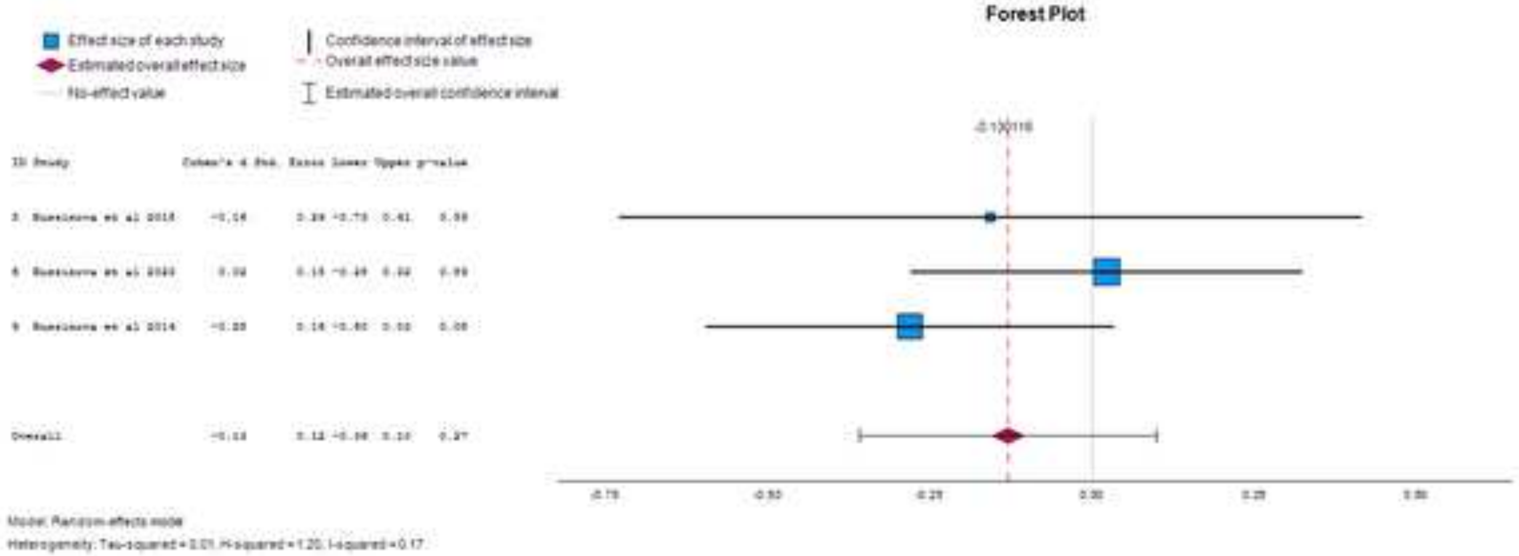

Figure Click here to access/download;Figure;Fig 4.tif

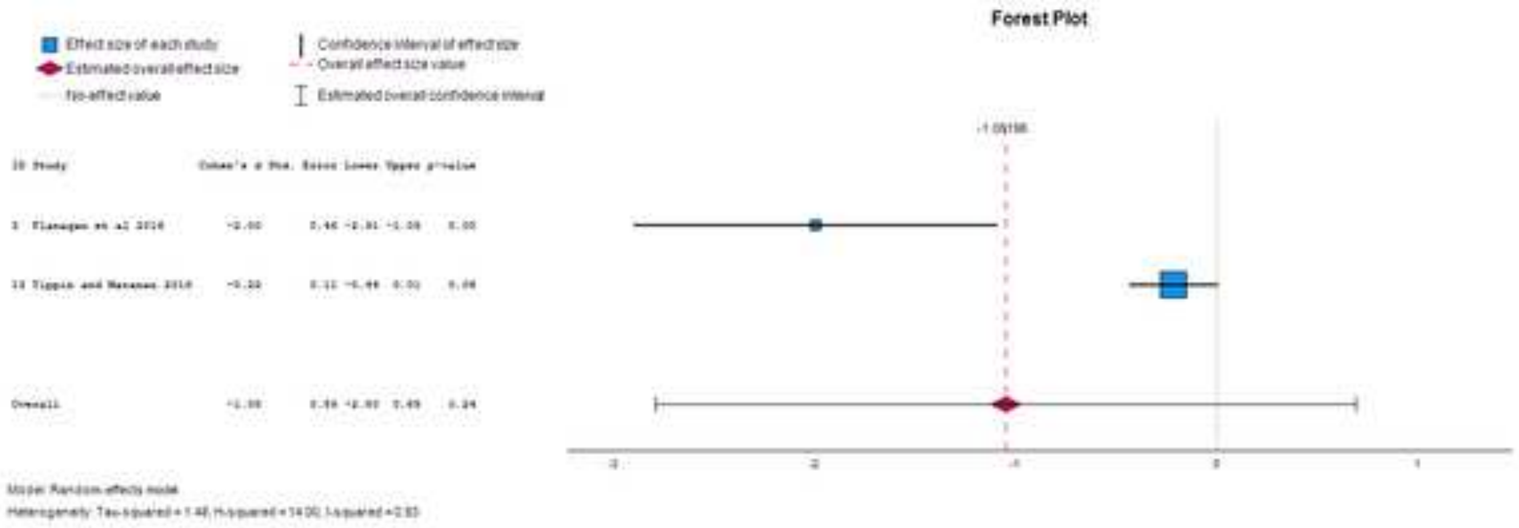

Figure

Click here to access/download;Figure;Fig 5 A to B.tif

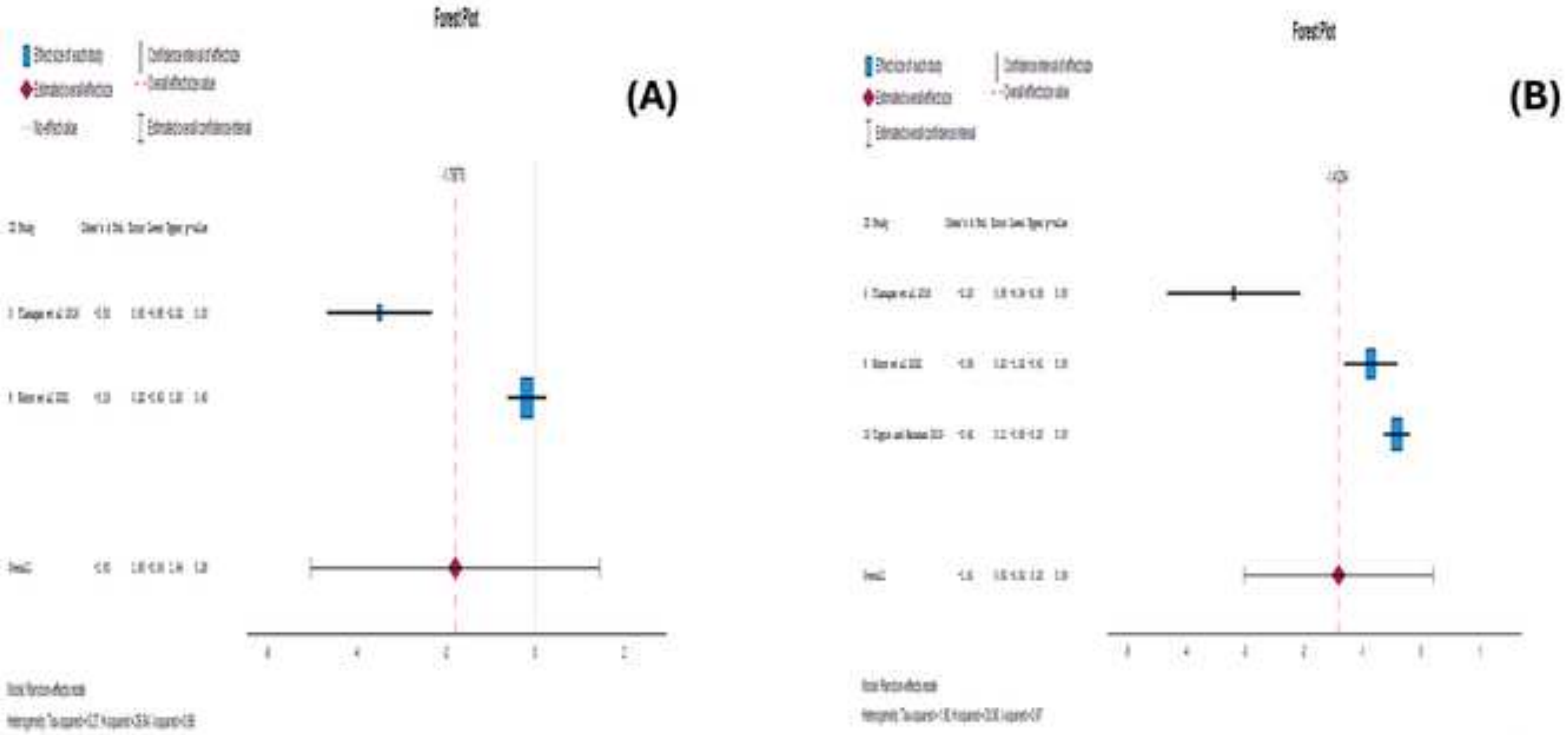

Click here to access/download
Supporting Information - Compressed/ZIP File Archive
supporting information for resubmission.zip

~~The~~The Impact of Photovoice on Mental Health and Stigma ~~effectiveness of Photovoice in mental health~~: A systematic review and meta-analysis.

Photovoice and mental health.

Adeniyi Adeboye[1¶], Whitney Onuorah[1&], Chizor Samuel-Nwokeji[1&], Ugochukwu Aghalu [1*], Chimaobi Nwanguma[1], Adekunle Akerele[2], Judy Wasige[1¶]

1 Department of Nursing and Community Health, School of Health and Life Sciences, Glasgow Caledonian University, Glasgow, Scotland, United Kingdom

2 Department of Epidemiology, Medical Statistics and Environmental Health, College of Medicine, University of Ibadan, Ibadan, Nigeria.

***** Corresponding author

E-mail: UAGHAL300@caledonian.ac.uk (UA)

¶These authors contributed equally to this work.

&These authors also contributed equally to this work.

Abstract

Background and Objective: Photovoice is a transformative tool for improving health outcomes among people living with mental ill-health (PLWMI), fostering awareness, community engagement, empowerment, and social change. Importantly, only one meta-analysis has evaluated the effectiveness of photovoice for mental health outcomes. However, the study focused on ~~a range of~~various health conditions and only addressed two mental health outcomes. This systematic review and meta-analysis, by exclusively evaluating the effect of photovoice on eight mental health and stigma-related outcomes in PLWMI and healthcare providers (HCPs), provides a comprehensive overview of the impact of photovoice ~~ion mental ill health and provides~~ on mental ill health and new evidence for practice.

Methods ~~and Study Design~~: Electronic database searches were conducted on Cochrane Trials, CINAHL Plus, ProQuest, Medline, and PsychInfo. Additionally, Google Scholar was searched to aid the retrieval of both published and unpublished Randomised Control Trials testing the effects of photovoice on PLWMI, HCPs or both from 2013 to 2023. ~~The quality of retrieved studies was assessed using the Effective Public Health Practice Project tool.~~ Random-effects meta-analyses ~~was~~were conducted for all outcomes evaluated in the study. A ~~within sample~~within-sample estimation of the effect size (Cohen's d) was calculated using the available raw data (mean and standard deviation) from each arm of each eligible study. The study protocol was pre-registered on PROSPERO, with registration number CRD42023476867.

Findings~~Results~~: Seven~~ix~~ studies (Sample size (n)=754~~819731~~) were included in the meta-analysis. The findings revealed that the application of photovoice resulted in a significant decrease in various mental health outcomes among PLWMI, including social withdrawal (3 studies, n=318; Cohen's d (*d*), 95% CI= -0.2, -0.41 to 0.00), depression (2 studies, n=267; *d*, 95% CI= -0.23, -0.45 to -0.02). Conversely, there was a significant increase in self-efficacy (2 studies, n=267; *d*, 95% CI= 0.22, 0.00 to 0.44) and recovery (3 studies, n=285~~350~~; *d*, 95% CI= 0.19, -0.02 to 0.41). Additionally, the stigma-related outcomes of PLWMI such as stigma resistance also decreased (3 studies, n=318; *d*, 95% CI= -0.13, -0.36 to -0.10). Furthermore, there was a significantly large reduction in the mental health-related outcome (anger) demonstrated by HCPs towards PLWMIs (2 studies, n=330; *d*, 95% CI= -1.05, -2.79 to 0.69). Additionally, there ~~was~~were also significantly large reductions in social distance (3 studies, n=418; *d*, 95% CI= -1.41, -3.02 to 1.03) and perception of danger (2 studies, n=115; *d*, 95% CI= -1.80, -5.04 to 1.44) which were classified as stigma-related outcomes in HCPs. ~~social distance (Effect size (Cohen's d (*d*), 95%CI= -1.20, -3.00 to 0.59), anger (*d*, 95%CI= -1.05, -2.79 to 0.69), stigma (*d*, 95%CI= -0.27, -0.53 to 0.00), social withdrawal (*d*, 95%CI= -0.2, -0.41 to 0.00), depression (*d*, 95%CI= -0.23, -0.45 to -0.02), and stigma resistance (*d*, 95%CI= -0.13, -0.36 to -0.10). Additionally, PLWMI exhibited significant improvements in self-efficacy (*d*, 95%CI= 0.22, 0.00 to 0.44) and recovery (*d*, 95%CI= 0.35, 0.00 to 0.69). Furthermore, one study (n=307) reported narratively suggests that photovoice may effectively reduce social distance and perception of danger towards PLWMI among healthcare providers (HCP).~~

Interpretation: The meta-analysis demonstrated that photovoice significantly improved several mental health outcomes in PLWMI. Specifically, photovoice was effective in reducing social withdrawal and depression among PLWMI. Moreover, the intervention led to significant increases in self-efficacy and

Formatted: Line spacing: Multiple 1.15 li

Formatted: Font: 12 pt

Formatted: Font: 12 pt

Formatted: Font: 12 pt

Formatted: Font: 12 pt

Formatted: Font: 12 pt

Formatted: Font: 12 pt

Formatted: Font: 12 pt

Formatted: Font: Bold, Italic

recovery of PLWMI. In terms of stigma-related outcomes, photovoice resulted in a reduction in stigma resistance among PLWMI. One plausible explanation of reduction in stigma resistance is the short duration of studies analysed for this category because the photovoice intervention increases participants' sensitivity to stigmatizing attitudes in the short term. In healthcare providers (HCPs), the findings indicated a significant reduction in negative mental health-related outcomes, such as anger towards PLWMI. Additionally, there were substantial decreases in social distance and the perception of danger, which are stigma-related outcomes in HCPs. Consequently, incorporating photovoice into mental health interventions could enhance patient mental health outcomes and reduce HCP-associated stigma, promoting better outcomes in PLWMI.This systematic review and meta-analysis reveals that photovoice is effective for improving mental health outcomes in PLWMI. Additionally, it shows that photovoice improves the behaviours of HCP towards PLWMI.

Conclusion: This systematic review and meta-analysis reveals that photovoice is effective for improving mental health outcomes in PLWMI. Additionally, it shows that photovoice improves the behaviours of HCP towards PLWMI.

Formatted: Font: Bold

Formatted: Indent: First line: 0", Line spacing: Multiple 1.15 li

Introduction

Photographs have been recognised as prominent sources of visual data [1], and narrative photography; a principle used in photovoice, which unveils the latent meaning of an image from the perspective of the reporter using storytelling, has garnered widespread adoption in research in recent times [2]. This is grounded in Goffman's symbolic interactionism, which posits that meanings are derived from people's interaction with symbols and other individuals [3]. Photovoice lends a crucial perspective in healthcare as it enables individuals to creatively elaborate the nuances of their health issues and lived experiences [4].

Notwithstanding an increased awareness ~~on~~ of mental health conditions, there is still a deficit of knowledge on how people with mental health issues deal with the challenging stressors associated with their conditions amidst their daily ~~life~~lives. This impedes the provision of relevant and adequate support [5]. According to extant studies, about 50-85% of the world population's mental health issues are currently untreated resulting in a high global burden of the condition [6]. Intentional avoidance of treatment due to stigma from healthcare providers (HCPs) ~~experiences~~ experienced ~~from~~ by people living with mental health illness (PLWMI)~~healthcare providers~~ ~~have~~ has been reported as one of the enabling factors of the increasing~~ed~~ number of untreated cases[7]. Consequently, explicit ~~HCP associated~~ stigma demonstrated by HCPs towards ~~people living with mental health illness (~~PLWMI~~)~~ has been linked with the underutilization of mental health care services among this population~~people living with mental health illness (PLWMI)~~ [8,9 ~~Kopera et al., 2015~~]. Likewise, implicit ~~healthcare provider (HCP) associated~~ stigma in HCPs has been related ~~with~~ to reduced detection of mental illness cases due to inadequate assessment of potential cases due to prejudice and discrimination [~~9,~~9,10 ~~Kopera et al., 2015~~]. This explicit~~ternal~~ or implicit ~~internalized~~ stigma faced by PLWMI results in hopelessness, low self-esteem, and reduced empowerment, quality of life, as well as social support, which gravely interferes with recovery [11~~0~~,12~~1~~].

Over the years, increased efforts have been made in developingto develop tools that would sufficiently support individuals with mental health conditions [132], while combatting stigma, internalization of negative stereotypes, and enhancement of self-efficacy [143]. Photovoice has been shown to have an inherent fluidity that enables its application as a methodology used in exploring various aspects of the lived experiences of people with mental ill health. The establishment of its methodology on Participatory Action Research (PAR) principles enables participants to play the role of collaborators and coresearchers, which asserts their wealth of experience in the conceptualization and development of suitable support to address their needs [1,154].

Photovoice avails PLWMI an innovative opportunity to tell their story, which aids in improving awareness of their lived experiences amongst the public, policy makerspolicymakers, service providers and other stakeholders, in a bid to drive social change [1,110,1615]. Likewise, it serves as an intervention that transforms individuals through empowerment and mitigation of harmful impacts of the stigma and discrimination faced by PLWMI [4,11]. Furthermore, photovoice facilitates the conduction of qualitative studies investigating sensitive and complex health conditions, including mental health issues [14-16], and studies have alluded to its effectiveness in alleviating negative mental health outcomes [4]. Photovoice can be utilised in two major ways, functioning both as a powerful intervention for improving mental health and as a robust qualitative methodology for eliciting outcomes suitable for quantitative evaluation [(Schenka et al., 2014; 17Woda et al., 2017]). As an intervention, one of the fundamental strengths of photovoice is its ability to transfer power to participants. By allowing individuals to document their lived experiences through photography, photovoice democratizes the research process, shifting control from the researcher to the participant [18(Call-Cummings et al., 2018]). This empowerment is particularly therapeutic for individuals with mental health issues, as it validates their experiences and provides a platform to voice their narratives [(12Russinova et al., 2018]). This transfer of power is crucial in mental health interventions, where

feeling heard and understood can significantly impact one's mental well-being [12(]Russinova et al., 2018).

Analysing the methodological underpinnings of photovoice, the data gathered through photovoice can uncover significant variables and outcomes that might be overlooked in traditional mental health research [(19]Christensen, 2019). Administering photovoice involves a series of steps designed to engage participants in capturing and reflecting on their lived experiences through photography. This process typically begins with participant recruitment and an orientation session where the aims of the study, ethical considerations, and technical aspects of photography are discussed [20](Wang & Burris, 1997). Participants are then tasked with taking photographs that represent their perspectives on a given issue, followed by a series of group discussions where they share their images and narratives. These discussions are crucial for co-constructing meaning and ensuring that the participants' voices are authentically represented [(21Carlson et al., 2006]). The facilitators play a significant role in guiding these discussions, encouraging deep reflection, and maintaining a non-judgmental space. One of the challenges in this process is ensuring that all participants are equally engaged and that power dynamics do not skew the interpretation of the visual data [(22Catalani & Minkler, 2010]). Moreover, the analysis of photovoice data requires a nuanced approach that considers both the visual and textual elements, often demanding interdisciplinary expertise [(23Liebenberg, 2018]). The insights derived from participants' photographs and the subsequent discussions can inform the development of more targeted and effective quantitative outcomes [(19Christensen, 2019]). For example, integrating photovoice methodology with surveys or standardized assessment tools can help quantify changes in mental health outcomes, such as reductions in anxiety or improvements in self-esteem. Additionally, photovoice methodology has been used to identify community-specific issues, such as the need for better HIV/AIDS education or the challenges faced by immigrant adolescents, which can then be quantified and addressed through targeted interventions [(24Hergenrather et al., 2009]).

Subsequently, photovoice's dual role as both an intervention for mental health and a methodology for quantitative outcome elicitation underscores its versatility and effectiveness. Its capacity to empower participants, raise consciousness, and foster community engagement, combined with its ability to generate rich qualitative data that can inform quantitative measures, makes photovoice a uniquely valuable tool in advancing mental health research and practice. Through this integrated approach, photovoice not only addresses immediate mental health needs but also contributes to long-term social change and policy development.

Consequently, this study evaluates the impact of photovoice ~~in~~ on mental ill health. It reviews appraised articles that report ~~on~~ the effect of photovoice on the~~in alleviating negative~~ mental health outcomes of PLWMI such as ~~outcomes such as stigma,~~ depression, recovery, self-efficacy, and social withdrawal, as well as reducing negative behaviours exhibited by HCPs towards PLWMI that ~~worsens exacerbates~~exacerbate mental ill health, such as anger. Furthermore, the study also assesses the impact of photovoice on the enhancement of positive stigma-related outcomes in~~of~~ PLWMI, including stigma resistance, in addition to the reduction of negative behaviours of HCPs that perpetuate feelings of stigma in PLWMI, such as the perception of danger and social distance.

In the context of this paper, mental health outcomes refer to outcomes that assess the internal psychic phenomena of patients, which cannot be externally examined or confirmed [~~(~~25~~Jacobs, 2009~~]~~)~~. These measures typically assess either the frequency and severity of symptoms related to mental health illnesses (psychopathological rating scales), or ~~they~~ evaluate the impact of mental health illnesses on an individual (measures of social functioning, or quality of life assessment) [25~~(Jacobs, 2009~~]~~)~~. Furthermore, stigma-related outcomes refer to the various consequences or impacts that stigma has on individuals living with mental illness (PLWMI), and their ability to overcome such consequences [~~(~~26~~Brohan et al., 2010~~]~~)~~. These outcomes can affect multiple aspects of a person's life, including their mental health, social interactions, access to healthcare, and overall quality of life [~~(~~26~~Brohan et al., 2010~~]~~)~~.

Formatted: Font color: Auto, Not Highlight

Formatted: Font color: Auto, Not Highlight

Formatted: Font color: Auto, Not Highlight

Formatted: Font: (Default) Times New Roman, 12 pt

Formatted: Font: (Default) Times New Roman, 12 pt

Formatted: Font: (Default) Times New Roman, 12 pt

Formatted: Font: (Default) Times New Roman, 12 pt

Formatted: Font: (Default) Times New Roman, 12 pt, Font color: Auto

augment positive outcomes such as self-efficacy, recovery, and stigma resistance in PLWMI as well as those that combat negative attitudes such as social distance and perception of danger towards PLWMI by HCP. The current study is expatiates on the works of Halvorsrud *et al.* [417], who conducted a systematic review and meta-analysis investigating the impact of photovoice on a range of health conditions such as diabetes, hepatitis, physical health, social function, and mental health. However, this is the first study to elaborately focus on investigating the facets of mental health and stigma as they relate to PLWMI and their HCPs, with analysis of eight key outcomes;outcomes: stigma, recovery, self-efficacy, stigma resistance, social withdrawal, depression, anger, social distancing, and perception of danger. This provides a comprehensive overview of the effectiveness of photovoice in mental health and informs new evidence for practice.

The study looks to answer the question "What is the impact of photovoice on mental health and stigma outcomes of PLWMI and HCPswhat is the impact of photovoice on mental health outcomes"? To this effect, fourtwo hypotheses were formulated. Firstly, the authors hypothesised that photovoice has a significant effect on improving the mental health outcomes of PLWMI. Secondly, it was hypothesised that photovoice significantly reduces has a significant effect on the negative mental health-related behaviours of HCP towards PLWMI. Thirdly, it was hypothesised that photovoice has a significant effect on improving the positive stigma-related outcomes of PLWMI. Fourthly, it was also hypothesised that photovoice significantly has a significantreduces the effect on the negative stigma-related behaviours of HCP towards PLWMI. Therefore, this study analysed the effectiveness of photovoice on the mental health and stigma outcomes of PLWMI and the behaviours of HCP HCPs towards PLWMI.

Methods

This review employed a systematic review and meta-analysis to investigate the effectiveness of photovoice in improving mental health outcomes among PLWMI and the attitudes of their HCP. It

Formatted: Font color: Auto, Not Highlight
Formatted: Font:
Formatted: Font color: Auto, Not Highlight
Formatted: Font color: Auto
Formatted: Font color: Auto, Not Highlight
Formatted: Font color: Auto, Not Highlight
Formatted: Font color: Auto, Not Highlight
Formatted: Font color: Auto, Not Highlight
Formatted: Font color: Auto, Not Highlight
Formatted: Font color: Auto, Not Highlight
Formatted: Font color: Auto, Not Highlight

was pre-registered on PROSPERO with identification number - CRD42023476867. A systematic review provides a comprehensive summary of evidence regarding a specific research focus through a systematic approach to identify, appraise, and synthesize all relevant evidence [27~~18~~,28~~19~~]. Additionally, meta-analysis involves statistical analysis that combines and synthesizes the results of numerous studies ~~with the aim of integrating their findings and producing~~to integrate their findings and produce a quantitative estimate [29,30~~20,21~~]. This proffers increased accuracy in effect estimation, conflict resolution between studies of the same foci, and generalizability of research findings [31,32~~22,23~~].

Eligibility Criteria

Types of studies

Primary quantitative experimental studies, exclusively randomized control trials or mixed methods studies incorporating a qualitative photovoice-based study as an intervention in a randomized controlled design, were included. [33~~24~~,34~~25~~]. The population of interest were PLWMI and their healthcare providers. No restrictions were implemented ~~in~~ on type of mental illness, age, or setting, and studies addressing mental health outcomes such as depression, recovery, self-efficacy, social withdrawal, stigma, and stigma-resistance and published in the English language from January 2013 to November 2023 were included.

Only studies with explicitly stated photovoice methodology, or participatory photography methodology that entailed training participants on photography and facilitating focused group discussions on photography and mental health were included. PLWMI would take photographic images that depicted their lived experiences of mental ill-health and interpret the photographs to the researcher to determine the meanings attributed. Participants were co-researchers and solely responsible for conceptualizing and allocating meaning to the pictures, as well as selecting photos and uncovering the stories behind them to the researchers [15~~2~~,16~~4~~,20~~6~~].

Included studies with healthcare practitioners measured the effectiveness of photovoice in ameliorating negative attitudes towards PLWMI. Excerpts from photovoice participatory studies would be introduced to some healthcare practitioners as an intervention and compared with others where there was either no intervention~~,~~ or practice as usual on anti-stigma interventions.

Search strategy and study selection

A scoping search was carried out on Cochrane Trials, CINAHL Plus, and Google Scholar to determine the availability of studies addressing the research question, inform the search development process, and keywords used in relevant articles [35~~27~~]. Between October to November 2023, five academic databases– Cochrane Trials, CINAHL Plus, ProQuest, Medline, and PsychInfo, were systematically perused for relevant papers. Google Scholar search engine was also incorporated to identify grey literature, and minimize publication bias [36~~28~~,37~~29~~], which promulgates comprehensiveness of the search process and evidence base, as well as minimizes omission of otherwise relevant studies which may not be present on generic databases [38~~0~~,39~~1~~]. Covidence automation software, alongside manual data management, ~~were~~ was incorporated in this review to ensure completeness in the retrieval of all available data, transparency, and bias reduction in the review process. Two reviewers, CN and JW independently screened the titles and abstracts of identified studies against the eligibility criteria, and an agreement was reached on the articles for full-text screening. Additionally, the reference lists of relevant articles were manually scanned for relevant papers. Concomitantly, CSJ and WO utilized Covidence for citation screening, data extraction and quality appraisal, which facilitated the reliability and ~~rigor~~ rigour of the review process ~~.~~ [40~~32~~]. The search process and study selection for inclusion ~~was~~ were stringently guided by the Preferred Reporting Items for Systematic Reviews and Meta-Analyses (PRISMA), for which a ~~flow diagram~~flow diagram was generated to aid transparency and reproducibility [41~~33~~,42~~34~~].

Data extraction

Data extraction was carried out independently by two researchers, CSJ and WO on Covidence. A form was developed from manuals of the Centre for Reviews and Dissemination, Joanna Briggs Institute, and consultation with experts ~~of~~ in the subject area [43~~35~~,44~~36~~]. This form was piloted on two included studies to ascertain suitability and adjusted as required [45~~37~~]. Data was extracted in textual, numerical, and tabular formats, and a third reviewer, AA, was invited to resolve any conflicts. This was done to ensure quality control, completeness, and reliability of extracted data [46~~38~~]. The data extraction focused on study characteristics (publication year, author, design, aim, sample size, survey instrument); participant characteristics (age, gender); intervention (photovoice and comparator); study outcome and effect size (mean, standard deviation, power calculation). Furthermore, AA and AA transferred the data into Microsoft excel which was already prepared with appropriate and relevant columns detailing shortlisted studies that met the eligibilities criteria. Variables extracted from these studies included mean/SD of study outcomes as well as effect sizes where they are available. Extracted data were shared with the members of the research team for second level of data verification before analysis was conducted. Standard error of each effect size was estimated using recommended formula as none was available in all the eight eligible studies [47].

Formatted: English (United States)

Quality assessment

Quality appraisal was done using a validated tool for quantitative studies, the Effective Public Health Practice Project [EPHPP] [48~~39~~] on Covidence. This assessed quality within six domains - selection bias; study design; confounders; blinding; data collection methods; withdrawals and dropouts. These result in an overall global rating, wherein studies with no weak ratings amongst the six domains are considered ‘strong,’ studies with one weak rating are considered ‘moderate,’ and studies with two or more weak ratings are considered ‘weak.’ Two reviewers, CSJ and WO,

independently assessed quality, and discordances were resolved through discussion, or by a third reviewer, AA.

Data Analysissynthesis

A meta-analysis was conducted to pool studies with similar outcomes. This was done in two phases; one phase focusing focused on studies on positive and negative mental health outcomes of PLWMI, and the other on studies on positive and negative attitudes of healthcare providers towards PLWMI. Precalculated effect size of photovoice on each outcome variable was identified and extracted. In the absence of precalculated effect size, Cohen's d (standardized mean difference) value was used to estimate effect size for the outcome variable. Variables appearing in less than two studies were not considered in the analysis. The pre and post mean, standard deviation, and precalculated effect size of each variable was extracted into a Microsoft excel spreadsheet and imported into SPSS version 28 for analysis.

Quality control was done and maintained by members of the review team to ensure validity. Raw data (i.e. mean and standard deviation of the two arms of each study) were used to calculate effect size from all the included studies using the recommended formula. This is a preferred approach to calculatecalculating effect size due to inconsistency in reporting the precalculated effect size across the studies extracted. This approach gives a concise estimate and direction of the effect size [49]. Based on the RCT study design of each eligible study with a focus on variable outcomes of interest, a within sample estimation of photovoice effectiveness was calculated using mean/standard deviation of each arm of each eligible study. Results reported included confidence intervals of effect size of each outcome, and overall effect size of the study outcome (Cohen's d). Due to expected differentials of the studies, random effect model was used for calculation of overall effect size of photovoice on each outcome [50]. Z distribution of 95% confidence intervals (CIs) was employed to estimate overall effect size. This approach is said to offer a parsimonious and narrower CI to the study outcomes when one

considers the available sample size of at least two studies per outcome [50]. To reduce the effect of disparity and to normalize the data, a log transformation of the precalculated effect size was conducted.

The I squared statistic (I^2) and Hegger test from forest plots were used to assess the heterogeneity of the precalculated effect size from various studies. Funnel plots were used to assess the publication bias of studies included in the analysis. Within-group heterogeneity was assessed using I^2-statistic with ≥50% threshold indicating substantial heterogeneity. The Forest plots were used to present the magnitude and direction of the overall effect size relative to the line of no effect. Estimation was done at a 5% significant level.

SPSS version 22.0 was used to conduct all analyses.

~~Quality control was done and maintained by members of the review team to ensure validity. Due to expected differentials of the studies, random effect model was used for calculation of overall effect size of photovoice on each outcome. To reduce the effect of disparity and to normalize the data, log transformation of precalculated and estimated effect size was conducted. The I squared statistic (I^2) and Hegger test from forest plot was used to assess heterogeneity of the precalculated effect size from various studies. Funnel plots were used to assess publication bias of studies included in the analysis. All results were reported 95% confidence interval. Threshold of I^2 greater than 80% was used to determine if the meta-analysis of an outcome should be reported.~~

Data Synthesis~~Analysis~~

Further data management, quality control and required analysis were carried out based on the recommended guidance [51~~17~~]. ~~AA and AA checked and extracted relevant data required for conducting meta-analyses into an Excel spreadsheet as a way of addressing the aim and objectives of the study.~~ Overall, four studies were considered to address the mental health-related outcomes in PLWMI. The outcomes considered included “Depression, Recovery, Self-Efficacy, and Social

Withdrawal". Additionally, three studies were considered to address stigma-related outcomes in PLWMI, with "stigma resistance" being the only outcome evaluated. Furthermore, in addressing the mental health-related outcomes in HCPs, three studies that met eligibility criteria were available for the analysis and one outcome, "anger" was evaluated for this analysis. Finally, addressing the stigma-related outcomes in HCPs necessitated the evaluation of three studies that met the criteria for two outcomes, "perception of danger" and social distance". AA and AA checked the already extracted relevant data, including mean/SD of study outcomes as well as effect sizes where available from the seven included studies for conducting meta-analyses as a way of addressing the two research objectives. Extracted data was shared with the members of the research team for second level of data verification before analysis was conducted. Overall, six studies were considered to address the mental health outcomes of PLWMI. The outcomes considered included anger, recovery, depression, social distance, stigma, self-efficacy, stigma resistance.

Overall effect size (Cohen's d) of each of these outcomes was calculated as a way of estimating effectiveness of photovoice among people living with mental illness. Z distribution of 95% confidence intervals (CIs) was employed to estimate overall effect size. This approach is said to offer a parsimonious and narrower CI to the study outcomes when one considers the available sample size [Goulet-Pelletier and Cousineau, 2018] of at least two studies per outcome. Raw data (i.e. mean and standard deviation of the two arms of each study) were used to calculate effect size using recommended formula. This is a preferred approach to calculate effect size due to inconsistency in reporting the precalculated effect size among few studies extracted. This approach gives a concise estimate and direction of the effect size [Tom and Osselaer, 2018]. Based on the RCT study design of each eligible study with a focus on variable outcomes of interest, a within sample estimation of photovoice effectiveness was calculated using mean/standard deviation of each arm of each eligible study. Results reported included confidence intervals of effect size of each outcome, and overall effect size of the study outcome (Cohen's d). Within-group heterogeneity was assessed using I^2 statistic with ≥50%

Formatted: Superscript

threshold indicating substantial heterogeneity. Forest plot was used to present magnitude and direction of the overall effect size relative to line of no effect. Estimation was done at 5% significant level.

SPSS version 22.0 was used to conduct all analyses.Overall effect size (Cohen's d) of each of these outcomes was calculated as a way of estimating effectiveness of photovoice among people living with mental illness.

Z distribution of 95% confidence intervals (CIs) was employed to estimate overall effect size. This approach is said to offer a parsimonious and narrower CI to the study outcomes when one considers the available sample size of at least two studies per outcome.

Raw data (that is, mean and standard deviation of the two arms of each study) were used to calculate effect size using recommended formula. This is a preferred approach to calculate effect size due to inconsistency in reporting the precalculated effect size among few studies extracted. This approach gives a concise estimate and direction of the effect size [40]. Based on the RCT study design of each eligible study with a focus on variable outcomes of interest, a within sample estimation of photovoice effectiveness was calculated using mean/standard deviation of each arm of each eligible study. Standard error of each effect size was estimated using recommended formula as none was available in all the eight eligible studies [41].

Results reported included confidence intervals of effect size of each outcome, overall effect size of the study outcome (Cohen's d). Within-group heterogeneity was assessed using I^2 statistic with ≥50% threshold indicating substantial heterogeneity. Forest plot was used to present magnitude and direction of the overall effect size relative to line of no effect. Estimation was done at 5% significant level.

In addressing the effect on the behaviours of HCP towards PLWMI , one study that met eligibility criteria was available for the analysis and the study offered precalculated effect sizes. The study

Formatted: Indent: First line: 0"

presented effect size of intervention relative to control groups. Hence, between sample, as opposed to within sample, effect size was estimated. Study outcomes considered for the sample of health caregivers included ''perception of danger'' and ''social distance scale''. Consequently, a meta-analysis could not be conducted for this outcome, and the results of this outcome was reported narratively.

Results

Study Selection

The database search identified a total of 331 potentially relevant studies which were imputed into Covidence for the removal of duplicates. At this stage, a total of 31 duplicates were removed, leaving a total of 300 studies for screening based on the titles and abstracts on Covidence. The titles and abstracts screening led to the removal of a further 283 articles, leaving a total of 17 articles for full textfull-text reading. At this stage, two reviewers read the full text of the 17 articles to determine their adherence to the eligibility criteria in the current review. This led to an exclusion of 10 articles, leaving a total of 7 articles for quality assessment in this review. Reasons for exclusion included the identification of 2 studies as study protocols, 2 studies did not utilise photovoice-based interventions, while and 6 studies were not randomised controlled trials. Fig 1 illustrates the study selection process for the current review. See Table 1 for the characteristics of the seven included studies.

Formatted: Caption

Fig 1: PRISMA flow chart.

~~Fig 1.tif~~Fig 1.tif

Fig 1.tif

Formatted: Normal

Formatted: Tab stops: Not at 4.09"

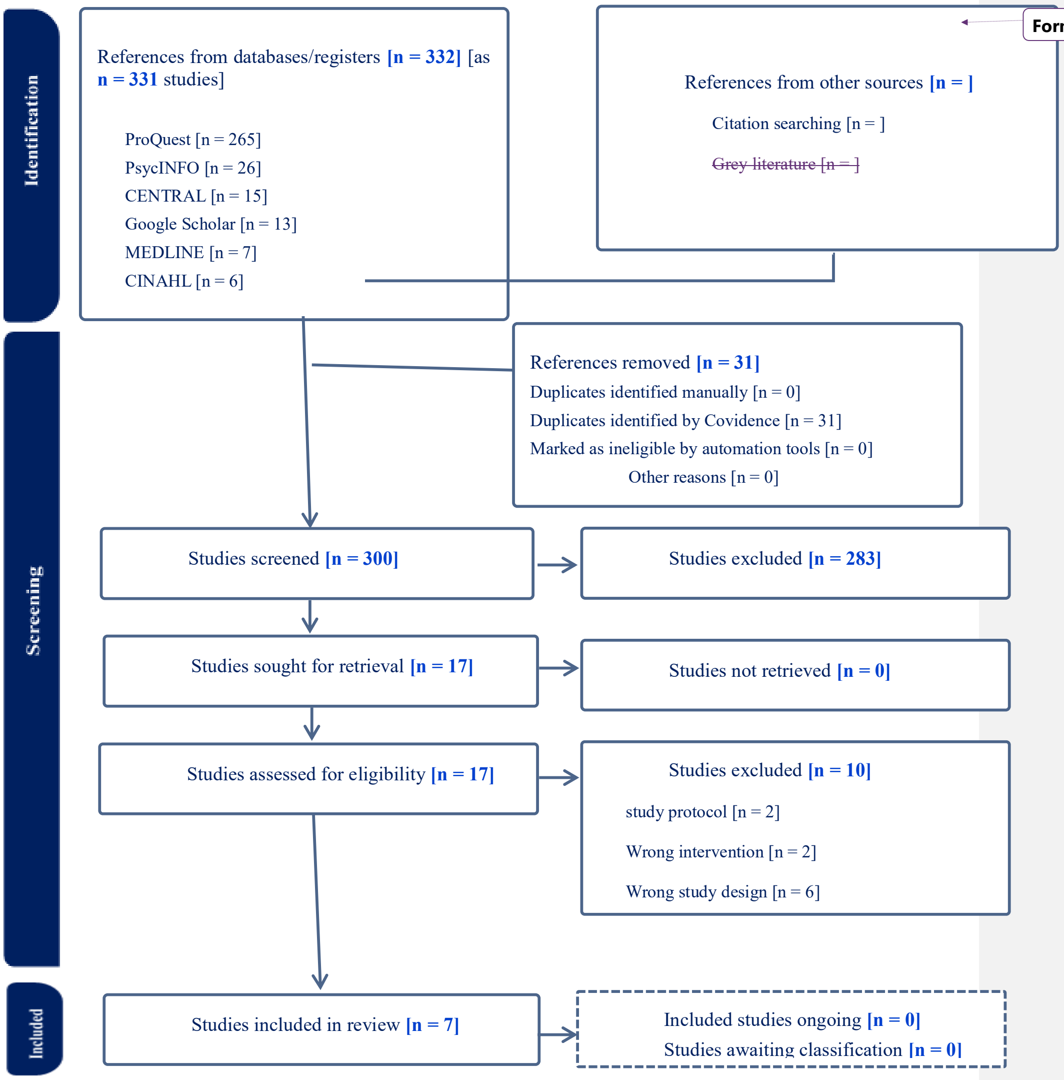

Table 1: Characteristics of included studies.

Study / Setting / Country	Questionnaire [Likert Scale]	Design	Intervention	Comparison	Outcomes	Gender/ Age	Sample size / Setting	Results
1. Zhang et al 2023 Hong Kong, China	Social Distance Scale (SDS) by PCPs	Mixed Methods (quantita-tive analysis designed as an RCT).	Mindfulness-Based Family Psychoeducation Intervention with a Photovoice component	Family Psychoedu-cation (FPE)	H1 – better caregiving experience for caregivers of YAIR H2 – better recovery for YAIR whose caregivers participated	78.5% female caregiv-ers	65 caregivers; 18 Young adults in recovery (YAIR)	integrating mindfulness into a conventional family psychoeducation program may reduce the expressed emotions of caregivers, especially over-involvement.
2. Flanagan et al 2016 USA	Link and Phelan's and Corrigan's definitions of stigma	RCT	1hr photovoice performance by PLWMI	No intervention	Stigma of PCPs	93% female / mean age ± SD 49.36±10.5	27 PCPs	significantly decreased negative stereotypes, attribution of dangerousness, fear, desire to coerce people into treatment,

Formatted: Highlight

Formatted: Highlight

Formatted: Highlight

								desire to segregate people from the community, and desire for avoidance.
3. Russinova et al 2018 USA	Employment and Vocational Activities Checklist * they used a LOT of scales to check several things	RCT	Vocational Empowerment Photovoice (VEP)	No intervention	Empower individuals with psychiatric disabilities to consider employment services and pursue work - work-related outcomes; extrinsic (enrollment in employment services and employm	Intervention group – 66.7% female; Control – 55.6% female	51 individuals with psychiatric disabilities	– significantly higher rate of engagement in employment services – significantly higher rate of engagement in employment services – increased work hope, self-efficacy and sense of vocational identity

Formatted: Highlight

					ent rates) and intrinsic (work hope, motivation and self-efficacy, vocational identity, overall empowerment and internalized stigma)			
4. Russinova et al 2023 USA	- Inventory of Community Participation - Mental Health (ICP-MH; Rogers et al., 2021) - Internalized Stigma of Mental Illness Scale (ISMI; Ritsher et al., 2003) etc	RCT	Bridging Community Gaps Photovoice (BCGP)" program	Services as usual	- self efficacy - self stigma - community engagement	Incterventention group - 60.6% female; Control - 61.5% female	185 participants	- increased community engagement - reduced self-stigma - significant impact on self-efficacy

Formatted: Highlight

5. Russinova et al 2014 USA	- Approaches to Coping with Stigma scales<br>- Internalized Stigma of Mental Illness Scale etc	RCT	peer-run photography-based intervention	No intervention	Self-stigma, coping with stigma, empowerment, perceived recovery, self-efficacy, and depression.	68% >40yrs; 68% female	82 PLWMI	significantly reduced self-stigma, greater use of proactive coping with societal stigma, greater increase in a sense of community activism, and perceived recovery and growth.<br>no differences between groups in regard to depression, self-efficacy, and other subscales of the internalized stigma, coping with stigma, and empowerment measures.
6. Tegerson et al 2021 Nepal	Social Distance Scale (SDS Implicit Association Test (IAT))	RCT in a mixed-method.<br>(2 RCTs from this sample.	Two arms of intervention were compared with the control arm. The first arm	Control with no videos	Perception of danger.<br>Social distance scale.	> 18 years for all sample; Mean age for	(Study 1: n = 94, Study 2: n = 213)	Contrary to expectations, including the psychosis service user testimonial video in addition to depression

Formatted: Highlight

Formatted: Highlight

		~~Study 1 on DEPRESSION only. Study 2 on DEPRESSION and PSYCHOSIS)~~	~~involved didactic videos which involved a lecture on mental health condition, and the second arm of the intervention involved a video created by a service user regarding his/her mental health experiences (photovoice-based). Th~~			~~the 3 arms~~<br>~~21.03 (1.14)~~<br>~~21.19 (1.11)~~<br>~~21.00 (1.08)~~<br>~~Age range for the 3 arms~~<br>~~-Male: 23 (24%) Female: 8 (8%)~~<br><br>~~-Male: 25 (27%)~~<br>~~Female: 6 (6%)~~<br><br>~~-Male:23 (24%) Female: 8 (8%)~~		~~videos may have increased explicit stigma towards both depression and psychosis.~~

7. Tippin and Maranzan 2019 Canada	Dangerousness Scale<br><br>Social Distance Scale (SDS)<br><br>Attribution Questionnaire<br><br>12-item self-report Level of Contact Report<br><br>Empathy Scale<br><br>40-item Balanced Inventory of Desirable Responding	RCT	Online anti-stigma photovoice-based video	Control video	Dangerousness, social distance, attributions (responsibility, anger, fear), mental health experience, empathy concern, social desirability	Female: 73.9%; Male:26.1%<br><br>Mean age - 21.44; SD (5.0)	n = 303	Photovoice-based video was efficacious in reducing mental illness stigma, including reduced fear and anger toward people with a mental illness, decreased perceptions of dangerousness, and decreased desired social distance. In addition, the intervention was efficacious in maintaining reduced desired social distance relative to the control at 1-month post-intervention. Finally, empathic concern was found to mediate the relationship between the Photovoice-based

Formatted: Highlight

video and reduced mental illness stigma, suggesting that the way the intervention reduced mental illness stigma was by eliciting empathy in the viewer

Study / Setting / Country	Population	Design	Intervention	Comparison	Outcomes	Outcome measures	Gender / Age	Sample size / Setting	Results
1. Zhang et al 2023 Hong Kong, China	PLWMI	Mixed Methods (quantita-tive analysis designed as an RCT).	Mindfulness-Based Family Psychoeducation Intervention with a Photovoice component	Family Psychoedu-cation (FPE)	H1 – better caregiving experience for caregivers of YAIR 1) H2 – better recovery for YAIR whose caregivers participated recovery	1) MHRM	78.5% female caregiv-ers	65 caregivers; 18 Young adults in recovery (YAIR)	integrating mindfulness into a conventional family psychoeducation program may reduce the expressed emotions of caregivers, especially over-involvement. Recovery level of YAIR increased after their caregivers participated in MBFPE
2. Flanagan et al	HCPs	RCT	1hr photovoice performance by PLWMI	No intervention	1) Stigma of PCPsanger 2) recovery	1) Attribution Q.	93% female / mean	27 HPCPs	significantly decreased negative

Formatted: Left

2016<br>USA					3) social distance	2) RKI<br>3) SDS	age ± SD 49.36± 10.5		stereotypes, attribution of dangerousness, fear, desire to coerce people into treatment, desire to segregate people from the community, and desire for avoidance.
3. Russinova et al<br>2018<br>USA	PLWMI	RCT	Vocational Empowerment Photovoice (VEP)	No intervention	Empower individuals with psychiatric disabilities to consider employment services and pursue work<br>- work-related outcomes;<br>1) extrinsic (enrollment in employment services and	ISMI	Interve-ntion group – 66.7% female; Control – 55.6% female	51 individuals with psychiatric disabilities	- significantly higher rate of engagement in employment services<br>- significantly higher rate of engagement in employment services<br>- increased work hope,

					~~employment rates) and intrinsic (work hope, motivation and self-efficacy, vocational identity, overall empowerment and internalized stigma)~~social withdrawal<br>2) stigma resistance				self-efficacy and sense of vocational identity
4. Russinova et al 2023 USA	PLWMI	RCT	Bridging Community Gaps Photovoice (BCGP)" program	Services as usual	~~- self efficacy~~<br>~~- self stigma~~<br>1) ~~- community engagem~~depression~~ent~~<br>2) recovery<br>3) self-efficacy<br>4) social withdrawal	1) BASIS-24<br>2) PGRS<br>3) ICP-MH<br>4&5) ISMI	Incterv ention group – 60.6% female; Control – 61.5% female	185 participants	- increased community engagement<br>- reduced self-stigma<br>- significant impact on self-efficacy

Formatted: Indent: Left: 0"

					5) stigma resistance				
5. Russinova et al 2014 USA	PLWMI	RCT	peer-run photography-based intervention	No intervention	1) Depression 2) Recovery 3) Self-efficacy 4) Social withdrawal 5) Stigma resistance Self-stigma, coping with stigma, empowerment, perceived recovery, self-efficacy, and depression.	1) Depression scale 2) PGRS 3) GPSES 4&5) ISMI	68% >40yrs; 68% female	82 PLWMI	significantly reduced self-stigma, greater use of proactive coping with societal stigma, greater increase in a sense of community activism, and perceived recovery and growth. no differences between groups in regard toabout depression, self-efficacyy, and other subscales of the internalized

									stigma, coping with stigma, and empowerment measures.
6. Kohrt et al 2021Tegerson et al 2021<br><br>Nepal	HCPs	Pilot Cluster RCT RCT in a mixed-method:<br><br>(2 RCTs from this sample. Study 1 on DEPRESSION only. Study 2 on DEPRESSION and PSYCHOSIS)	RESHAPE training Two arms of intervention were compared with the control arm. The first arm involved didactic videos which involved a lecture on mental health condition, and the second arm of the intervention involved a video created by a service user regarding his/her mental health experiences	Training as usual (mhGAP-IG)Control with no videos	1) Perception of danger.<br><br>2) Social distance scale.	1) SDS<br>2) IAT	85% male / mean age ± SD 36.2 ± 8.8 years ≥ 18 years for all sample; Mean age for the 3 arms 21.03 (1.14) 21.19 (1.11) 21.00 (1.08) Age range	88 PCPs(Study 1: n = 94, Study 2: n = 213)	reduced stigmaContrary to expectations, including the psychosis service user testimonial video in addition to depression videos may have increased explicit stigma towards both depression and psychosis. compared with standarad training

Formatted: Font color: Auto

			(photovoice-based). Th				for the 3 arms<br>-Male: 23 (24%) Female: 8 (8%)<br>-Male: 25 (27%) Female: 6 (6%)<br>-Male:23 (24%) Female: 8 (8%)		
7. Tippin and Maranzan 2019 Canada	HCPs	RCT	Online anti-stigma photovoice-based video	Control video	1) Dangerousness, social distance, attributions (responsibility, anger, fear), mental healthanger	1) Atrribution Q<br>2) SDS	Female: 73.9%; Male:26.1%<br>Mean age - 21.44; SD (5.0)	n = 303	Photovoice-based video was efficacious in reducing mental illness stigma, including ; reduced fear and anger

Formatted: Font color: Auto

					2) social distance experience, empathy concern, social desirability					toward people with a mental illness,PLWMI, decreased perceptions of dangerousness, and decreased desired social distance. In addition, the intervention was efficacious in maintaining reduced desired social distance relative to the control at 1-month post-intervention. Finally, empathic concern was found to mediate the relationship between the

									~~Photovoice-based video and reduced mental illness stigma, suggesting that one way the intervention reduced mental illness stigma was by eliciting empathy in the viewer~~

KEY

HCPs – Healthcare Practitioners

MBFPE - Mindfulness-based Family Psychoeducation

PLWMI - People living with mental illness

YAIR - Young adults in recovery

BASIS-24 – Behaviour and Symptom Identification Scale

GPSES – Generalized Perceived Self-efficacy Scale

IAT – Implicit Attitude Test

ICP – MH – Inventory of Community Participation – Mental Health

ISMI – Internalized Stigma of Mental Illness

MHRM – Mental Health Recovery Measure

PGRS – Personal Growth and Recovery Scale

- RKI – Recovery Knowledge Inventory
- SDS – Social Distance Scale

Formatted: Font: Bold

Formatted: Tab stops: 1.24", Left

Formatted: Justified, Indent: First line: 0.5", Space Before: 4 pt, After: 10 pt, Line spacing: Double, Tab stops: 1.24", Left

Quality Assessment results

As earlier indicated, the seven included studies were assessed for quality by utilising the EPHPP tool. The EPHPP tool on Covidence facilitated the extraction of relevant data for quality assessment. Regarding selection bias, two studies were rated strong, while five studies were rated moderate. Regarding the study design, all included studies were rated strong which is a characteristic shortcoming of the EPHPP tool as RCTs are automatically assigned a strong rating for this domain.

Regarding the influence of confounders on the outcomes, two studies received a strong rating which indicated that there were no differences in baseline measures between intervention and control groups, while five studies were rated weak due to differences between groups at baseline.

Only one study received a strong rating for blinding which indicated that the research question was concealed from participants and that outcome assessors were not aware of the intervention status of participants, while the remaining studies received a moderate rating for this category.

Additionally, all studies utilised valid and reliable tools for data collection, with full details provided on how the tools were validated before data collection leading to a strong rating for all included studies in this domain.

Regarding withdrawals and dropouts, two studies were rated strong because they reported the withdrawals fully, including reasons for loss of participants and above 80% of study participants completed the studies. Additionally, 3 studies received a moderate rating due to recording a 75% study completion rate among study participants. Furthermore, 2 studies received a weak rating in this category. One of the studies recorded less than 60% study completion rate, in contrast to the final study that did not report on withdrawals. Overall, only one study was assessed as strong indicating a high quality, five studies were assessed as moderate indicating moderate quality, while one study was assessed as weak indicating a weak quality.

Table 2 details the methodological quality assessment conducted based on the EPHPP tool.

Table 2: EPHPP tool Quality assessment results

		Khort et al 2023~~Torgesen et al 2021~~	Flanagan *et al* 2016	Zhang *et al* 2023	Russinova *et al* 2014	Russinova *et al* 2018	Russinova *et al* 2023	Tippin and Maranzan 2019
**A. SELECTION BIAS**								
Q1	Representative of the target population?	VL	SL	SL	SL	SL	VL	SL
Q2	Percentage of selected that agreed to participate?	100%	CT	CT	CT	CT	80-100%	CT
Section Rating		Strong	Mod	Mod	Mod	Mod	Strong	Mod
**B. STUDY DESIGN**								
Q1	Described as RCT?	Yes	Yes	Yes	Yes	Yes	Yes	Yes
Q2	Randomization method described?	Yes	No	Yes	No	No	No	No
Q3	Randomization method appropriate?	Yes	N/A	Yes	No	No	No	No
Section Rating		Strong	Strong	Strong	Strong	Strong	Strong	Strong
**C. CONFOUNDERS**								
Q1	Important differences between groups before intervention?	No	CT	CT	No	Yes	CT	CT
Q2	If Yes, percentage of confounders controlled?	N/A	N/A	N/A	N/A	CT	CT	CT
Section Rating		Strong	Weak	Weak	Strong	Weak	Weak	Weak
**D. BLINDING**								
Q1	Outcome assessors aware of intervention status of participants?	Yes	Yes	CT	Yes	Yes	CT	No
Q2	Study participants aware of the research question?	No	No	No	CT	CT	CT	No
Section Rating		Mod	Mod	Mod	Mod	Mod	Mod	Strong
**E. DATA COLLECTION METHODS**								
Q1	Data collection tool valid?	Yes	Yes	Yes	Yes	Yes	Yes	Yes
Q2	Data collection tool reliable?	Yes	Yes	Yes	Yes	Yes	Yes	Yes
Section Rating		Strong	Strong	Strong	Strong	Strong	Strong	Strong
**F. WITHDRAWALS**								
Q1	Withdrawals and dropouts reported?	Yes	No	Yes	Yes	Yes	Yes	Yes
Q2	Indicated the percentage of participants that completed the study?	93%	N/A	N/A	75%	75%	90%	<60%
Section Rating		Strong	Weak	Mod	Mod	Mod	Strong	Weak
**Global Rating For Each Study**		**STRONG**	**WEAK**	**MOD**	**MOD**	**MOD**	**MOD**	**WEAK**

KEY: VL – Very Likely; SL – Somewhat Likely; CT – Can't Tell; MOD – Moderate; N/A – Not Available

Adapted from: Effective Public Healthcare Panacea Project [2009] *Quality Assessment Tool for Quantitative Studies*, *Effective Public Healthcare Panacea Project*. Available at: https://www.ephpp.ca/quality-assessment-tool-for-quantitative-studies

Formatted: Not Highlight

Effect of photovoice on the mental health-related outcomes in PLWMI

Formatted: Heading 2, Space After: 0 pt, Line spacing: single

Depression

Two papers provided data on the effect of photovoice on depression as an outcome of mental illness. ~~Overall~~The overall effect size was -0.23 (95% CI, - 0.45 to -0.02) demonstrating a 23% reduction in depression following the use of photovoice. The forest plot of the effect size of photovoice on depression is presented in Fig 2A.

Formatted: Justified, Indent: First line: 0.5", Space Before: 4 pt, After: 10 pt, Line spacing: Double

Recovery

Formatted: Heading 3, Space After: 0 pt, Line spacing: single

Three papers provided data on the effect of photovoice on recovery as an outcome of mental illness. The o~~O~~verall effect size was 0.19 (95% CI, -0.02 to 0.41) demonstrating a 19% chance of recovery from mental illness when photovoice is used. The forest plot of the effect size of photovoice on recovery is presented in Fig 2B.

Formatted: Justified, Indent: First line: 0.5", Space Before: 4 pt, After: 10 pt, Line spacing: Double

Self-Efficacy

Formatted: Heading 3, Space After: 0 pt, Line spacing: single

Two papers provided data on the effect of photovoice on self-efficacy as an outcome of mental illness. The o~~O~~verall effect was 0.22 (95% CI, 0.00 to 0.44) demonstrating a 22% increase in self-efficacy after photovoice was used. The forest plot of the effect size of photovoice on self-efficacy is presented in Fig 2C.

Formatted: Justified, Indent: First line: 0.5", Space Before: 4 pt, After: 10 pt, Line spacing: Double

Social Withdrawal

Formatted: Heading 3, Space After: 0 pt, Line spacing: single

Three papers provided data on the effect of photovoice on self-withdrawal as an outcome of mental illness. The o~~O~~verall effect size was -0.2 (95% CI, -0.41 to 0.00) demonstrating a 20% reduction in social withdrawal after photovoice was used. The forest plot of the effect size of photovoice on anger is presented in Fig 2D.

Fig 2: Forest Plot of Effect Size of photovoice on mental health-related outcomes of PLWMI.

fig 2 A to D.tif (A) Depression, (B) Recovery, (C) Self-Efficacy (D) Social withdrawal.

Effect of photovoice on the stigma-related outcomes in PLWMI

Stigma Resistance

Three papers provided data on the effect of photovoice on stigma resistance as an outcome of mental illness. The o~~O~~verall effect size was -0.13 (95% CI, -0.36 to 0.10) demonstrating a 13% reduction in stigma resistance after photovoice was used. The forest plot of the effect size of photovoice on stigma resistance is presented in Fig 3.

Fig 3: Forest Plot of Effect Size of photovoice on stigma-related outcomes of PLWMI.

fig 3.tif

Effect of photovoice on the mental health-related outcomes in HCPs

Anger

Two papers provided data on the effect of photovoice on anger as an outcome of mental illness. ~~Overall~~The overall effect size of photovoice on anger was -1.05 (95% CI, - 2.80 to 0.69). This demonstrates that photovoice reduced anger in study participants by 105% following the use of photovoice. The forest plot of the effect size of photovoice on anger is presented in Fig 4.

Fig 4: Forest Plot of Effect Size of photovoice on mental health-related outcomes in HCPs.

Fig 4.tif

Formatted: Caption

Formatted: Heading 2

Formatted: English (United Kingdom)

Formatted: Caption

Formatted: Heading 2, Space After: 0 pt, Line spacing: single

Formatted: Caption

Formatted: Font: Bold, English (United Kingdom)

Formatted: Justified, Indent: First line: 0.5", Space Before: 4 pt, After: 10 pt, Line spacing: Double

Effect of photovoice on the stigma-related outcomes in HCPs

Social distance

Three papers were utilised to extract data on the ~~health caregiver's use of photovoice generated~~photovoice-generated effect size for social distancing in HCPs~~scale~~. Overall, the effect size of photovoice on social distance in HCP~~health caregiver~~s was -1.41 (95% CI, -3.02 to 1.03). This demonstrates that photovoice reduced social distance in study participants by 141% following use of photovoice. The forest plot of the effect size of photovoice on social distance is presented in Fig 5A.

Perception of Danger

Two papers were utilised to extract data on the photovoice-generated effect size for social distancing in HCPs.~~used to extract data on health caregiver's use of photovoice generated effect size for perception of danger.~~ Photovoice had a -1.80 (95% CI, -5.04 to 1.44) effect size on the perception of danger in HCPs~~of health caregivers~~. This demonstrates that photovoice reduced the perception of danger in study participants by 180% following the use of photovoice. The forest plot of the effect size of photovoice on perception of danger is presented in Fig 5B. Table 3 details the effect sizes calculated from the available raw data for all outcomes.

Fig 5: Forest Plot of Effect Size of photovoice on stigma-related outcomes in HCPs.

Fig 5 A to B.tif (A) Social Distance (B) Perception of Danger.

Formatted: Heading 2, Space After: 0 pt, Line spacing: single

Formatted: Justified, Indent: First line: 0.5", Space Before: 4 pt, After: 10 pt, Line spacing: Double

Formatted: Heading 3, Space After: 0 pt, Line spacing: single

Formatted: Caption

Formatted: Tab stops: 0.87", Left

Table 3: Effect Size of photovoice on mental health and stigma-related outcomes in both PLWMI and HCPs.

**Outcome**	**Study ID**	**Cohen's d**	**Std. Error**	**95% C I**		**Heterogeneity**
				**Lower**	**Upper**	
**Mental Health related outcomes in PLWMI**						
Depression	Russionova et al 2014	-0.33	0.16	-0.63	0.03	Tau-squared =0.00<br>H-squared=1.00<br>I-squared= 0.00
	Russinova et al 2023	-0.13	0.16	-0.45	0.18	
	Overall	-0.23	0.11	-0.45	-0.02	
Recovery	Zhang et al 2023	0.88	0.52	-0.15	1.90	Tau-squared=0.00<br>H-squared = 1.00<br>I-squared= 0.00
	Russinova et al 2014	0.16	0.15	-0.14	0.46	
	Russionova et al 2023	0.17	0.16	-0.15	0.48	
	Overall	0.19	0.11	-0.02	0.41	
Self-Efficacy	Russionova et al 2014	0.27	0.15	-0.04	0.57	Tau-squared=0.00<br>H-squared = 1.00<br>I-squared= 0.00
	Russinova et al 2023	0.18	0.16	-0.14	0.49	
	Overall	0.22	0.11	0.00	0.44	
Social Withdrawal	Russionova et al 2014	-0.31	0.29	-0.88	0.27	Tau-squared=0.00<br>H-squared = 1.00<br>I-squared= 0.00
	Russionova et al 2018	-0.17	0.15	-0.47	0.13	
	Russionova et al 2023	-0.21	0.16	-0.52	0.11	
	Overall	-0.20	0.10	-0.41	0.00	
**Mental Health related Outcomes in HCPs**						
Anger	Flanagan et al 2016	-2.00	0.46	-2.91	-1.09	Tau-squared=1.48

Formatted: Default Paragraph Font, Font color: Text 1, English (United Kingdom), Pattern: Clear
Formatted: Caption, Space After: 0 pt, Line spacing: single
Formatted: Default Paragraph Font, Font color: Text 1, English (United Kingdom), Pattern: Clear
Formatted: Font: Bold
Formatted: Left
Formatted Table
Formatted: Font: Bold
Formatted: Left
Formatted: Font: Bold
Formatted: Left
Formatted: Left
Formatted: Left
Formatted: Left
Formatted: Left
Formatted: Left
Formatted: Left
Formatted: Left
Formatted: Left
Formatted: Font: Bold
Formatted: Left
Formatted: Font: Bold
Formatted: Left
Formatted: Left

**Outcome**	**Study ID**	**Cohen's d**	**Std. Error**	**95% C I**		**Heterogeneity**
				**Lower**	**Upper**	
	Tippin and Maranzan 2019	-0.22	0.11	-0.44	0.01	H-squared = 14.00<br>I-squared= 0.93
	Overall	-1.05	0.89	-2.80	0.69	
**Stigma-related outcomes in PLWMI**						
Stigma Resistance	Russionova et al 2014	-0.16	0.29	-0.73	0.41	Tau-squared=0.01<br>H-squared = 1.20<br>I-squared= 0.17
	Russionova et al 2018	0.02	0.15	-0.28	0.32	
	Russinova et al 2023	-0.28	0.16	-0.60	0.02	
	Overall	-0.13	0.12	-0.36	0.10	
**Stigma-related outcomes in HCPs**						
Social Distance	Flanagan et al 2016	-3.20	0.58	-4.34	-2.06	Tau-squared=1.90<br>H-squared = 33.86<br>I-squared= 0.97
	Tippin and Maranzan 2019	-0.86	0.22	-1.32	-0.41	
	Kohrt et al 2021	-0.42	0.11	-0.65	-0.20	
	Overall	-1.41	0.82	-3.02	0.20	
Perception of danger	Kohrt et al 2021	-3.5	0.60	-4.68	-2.32	Tau-squared=5.27<br>H-squared = 26.64<br>I-squared= 0.96
	Flanagan et al 2016	-0.19	0.22	-0.63	0.25	
	Overall	-1.80	1.65	-5.04	1.44	

~~Data Analysis~~

~~Further data management, quality control and required analysis were carried out based on the recommended guidance [17]. AA and AA checked the already extracted relevant data, including mean/SD of study outcomes as well as effect sizes where available from the seven included studies for conducting meta-analyses as a way of addressing the two research objectives. Extracted data was shared with the members of the research team for second level of data verification before analysis was~~

Formatted: Font: Bold
Formatted: Left
Formatted Table
Formatted: Font: Bold
Formatted: Left
Formatted: Left
Formatted: Font: Bold
Formatted: Left
Formatted: Left
Formatted: Left
Formatted: Font: Bold
Formatted: Left
Formatted: Left
Formatted: Left
Formatted: Left
Formatted: Left
Formatted: Left

conducted. Overall, six studies were considered to address the mental health outcomes of PLWMI. The outcomes considered included anger, recovery, depression, social distance, stigma, self-efficacy, stigma resistance. Overall effect size (Cohen's d) of each of these outcomes was calculated as a way of estimating effectiveness of photovoice among people living with mental illness.

Z distribution of 95% confidence intervals (CIs) was employed to estimate overall effect size. This approach is said to offer a parsimonious and narrower CI to the study outcomes when one considers the available sample size of at least two studies per outcome.

Raw data (that is, mean and standard deviation of the two arms of each study) were used to calculate effect size using recommended formula. This is a preferred approach to calculate effect size due to inconsistency in reporting the precalculated effect size among few studies extracted. This approach gives a concise estimate and direction of the effect size [40]. Based on the RCT study design of each eligible study with a focus on variable outcomes of interest, a within sample estimation of photovoice effectiveness was calculated using mean/standard deviation of each arm of each eligible study. Standard error of each effect size was estimated using recommended formula as none was available in all the eight eligible studies [41].

Results reported included confidence intervals of effect size of each outcome, overall effect size of the study outcome (Cohen's d). Within-group heterogeneity was assessed using I^2-statistic with ≥50% threshold indicating substantial heterogeneity. Forest plot was used to present magnitude and direction of the overall effect size relative to line of no effect. Estimation was done at 5% significant level.

In addressing the effect on the behaviours of HCP towards PLWMI , one study that met eligibility criteria was available for the analysis and the study offered precalculated effect sizes. The study presented effect size of intervention relative to control groups. Hence, between sample, as opposed to within sample, effect size was estimated. Study outcomes considered for the sample of

health caregivers included ''perception of danger'' and ''social distance scale''. Consequently, a meta-analysis could not be conducted for this outcome, and the results of this outcome was reported narratively.

Effect of Photovoice on PLWMI

Anger

Two papers provided data on effect of photovoice on anger as an outcome of mental illness. Overall effect size was -1.05 (95% CI, -2.79 to 0.69) demonstrating 105% reduction in anger following the use of photovoice. The forest plot of the effect size of photovoice on anger is presented in Fig 2A.

Depression

Two papers provided data on effect of photovoice on depression as an outcome of mental illness. Overall effect size was -0.23 (95% CI, - 0.45 to -0.02) demonstrating 23% reduction in depression following the use of photovoice. The forest plot of the effect size of photovoice on depression is presented in Fig 2B..

Recovery

Four papers provided data on effect of photovoice on recovery as an outcome of mental illness. Overall effect size was 0.35 (95% CI, 0.00 to 0.69) demonstrating a 35% chance of recovery from mental illness when photovoice is used. The forest plot of the effect size of photovoice on recovery is presented in Fig 2C.

Social withdrawal

Three papers provided data on effect of photovoice on self-withdrawal as an outcome of mental illness. Overall effect size was -0.2 (95% CI, -0.41 to 0.00) demonstrating a 20% reduction in social

withdrawal after photovoice was used. The forest plot of the effect size of photovoice on social withdrawal is presented in Fig 2D.

Formatted: Normal

Stigma Resistance

Three papers provided data on effect of photovoice on stigma resistance as an outcome of mental illness. Overall effect size was -0.13(95% CI, -0.36 to -0.10) demonstrating 13% reduction in stigma resistance after photovoice was used. The forest plot of the effect size of photovoice on recovery is presented in Fig 2E.

Formatted: Normal

Self-Efficacy

Two papers provided data on effect of photovoice on self-efficacy as an outcome of mental illness. Overall effect was 0.22 (95% CI, 0.00 to 0.44) demonstrating a 22% increase in self-efficacy after photovoice was used. The forest plot of the effect size of photovoice on self-efficacy is presented in Fig 2.

Social distance

Three papers provided data on effect of photovoice on self-distancing as an outcome of mental illness. Overall effect size was -1.2 (95% CI, -3 to 0.59) demonstrating a 120% reduction in social distancing due to mental illness after photovoice was used. The forest plot of the effect size of photovoice on social distance is presented in Fig 2F.

Social withdrawal

Three papers provided data on effect of photovoice on self-withdrawal as an outcome of mental illness. Overall effect size was -0.2 (95% CI, -0.41 to 0.00) demonstrating a 20% reduction in social

withdrawal after photovoice was used. The forest plot of the effect size of photovoice on social withdrawal is presented in Fig 2.

Stigma

Two papers provided data on effect of photovoice on stigma as an outcome of mental illness. Overall effect was -0.27(95% CI, -0.53 to 0.00) demonstrating a 27% reduction in stigma following the use of photovoice. The forest plot of the effect size of photovoice on recovery is presented in Fig 2G.

Self-Efficacy

Two papers provided data on effect of photovoice on self-efficacy as an outcome of mental illness. Overall effect was 0.22 (95% CI, 0.00 to 0.44) demonstrating a 22% increase in self-efficacy after photovoice was used. The forest plot of the effect size of photovoice on self-efficacy is presented in Fig 2H.

Stigma Resistance

Three papers provided data on effect of photovoice on stigma resistance as an outcome of mental illness. Overall effect size was -0.13(95% CI, -0.36 to -0.10) demonstrating 13% reduction in stigma resistance after photovoice was used. The forest plot of the effect size of photovoice on recovery is presented in Fig 2.

Table 3 details the effect sizes calculated from the available raw data to addressing outcomes of mental illness.

~~Table 3: Effect Size of photovoice calculated from raw data addressing outcomes of mental illness.~~

Formatted: Normal

Formatted: Left: 1", Right: 1", Top: 0.75", Bottom: 0.75", Width: 8.27", Height: 11.69"

~~**Fig 2: Forest Plot of Effect Size of photovoice on outcomes of mental illness**~~

~~fig 2.tifFig 2.zip~~

Formatted: Default Paragraph Font

Formatted: Indent: First line: 0"

~~fig 2 E to H tif~~

Formatted: Normal

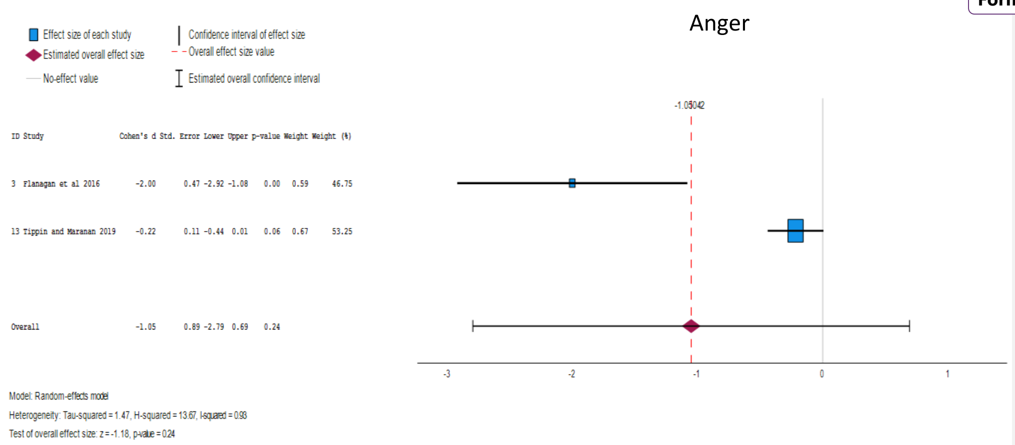

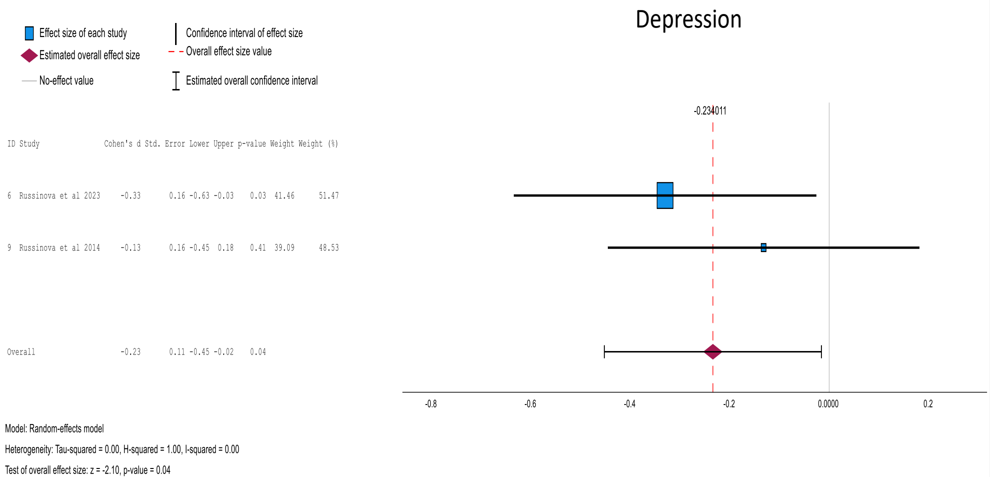

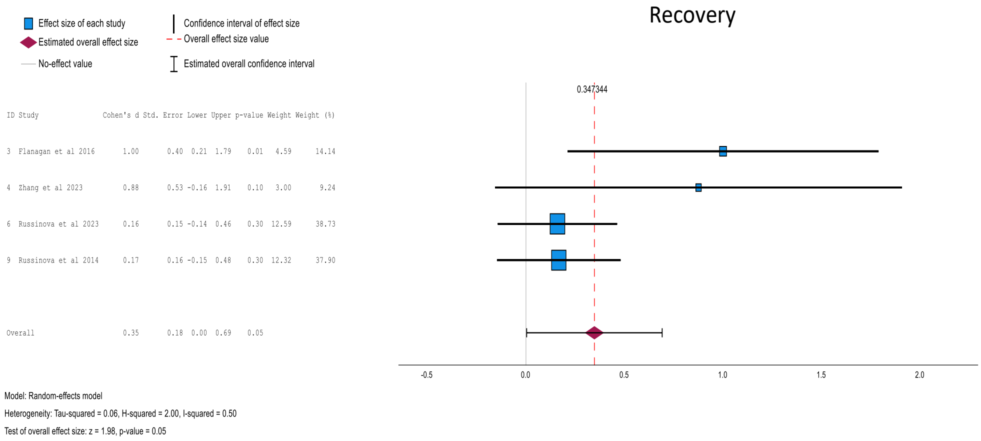

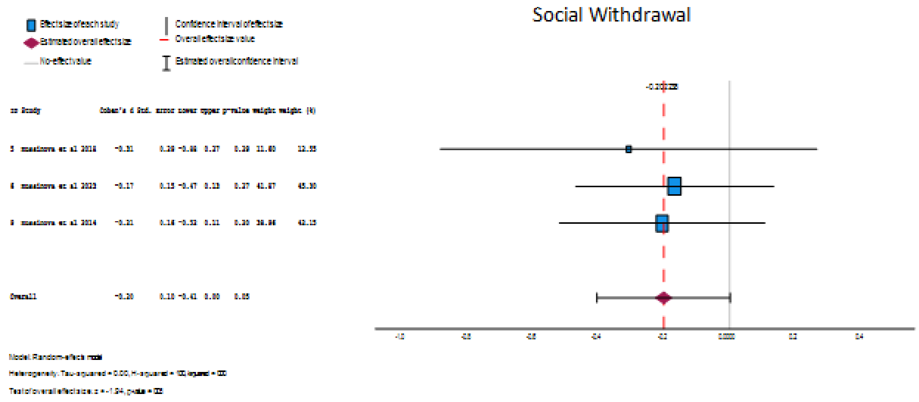

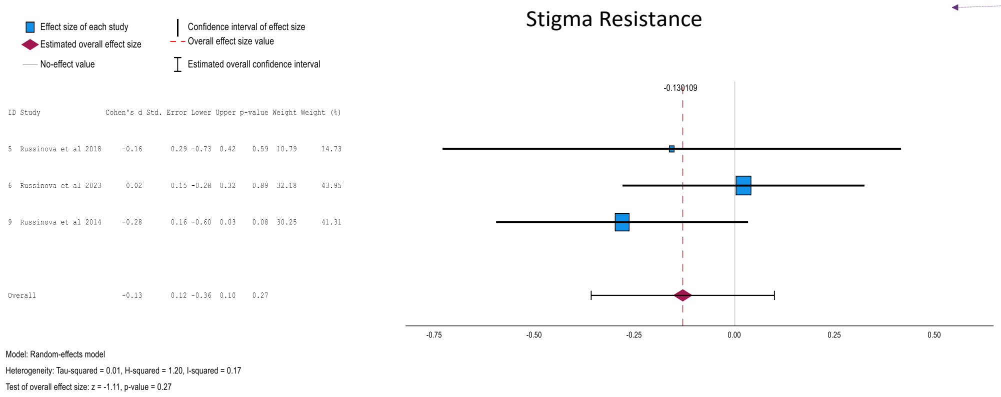

Formatted: Justified, Space Before: 4 pt, After: 10 pt, Line spacing: Double

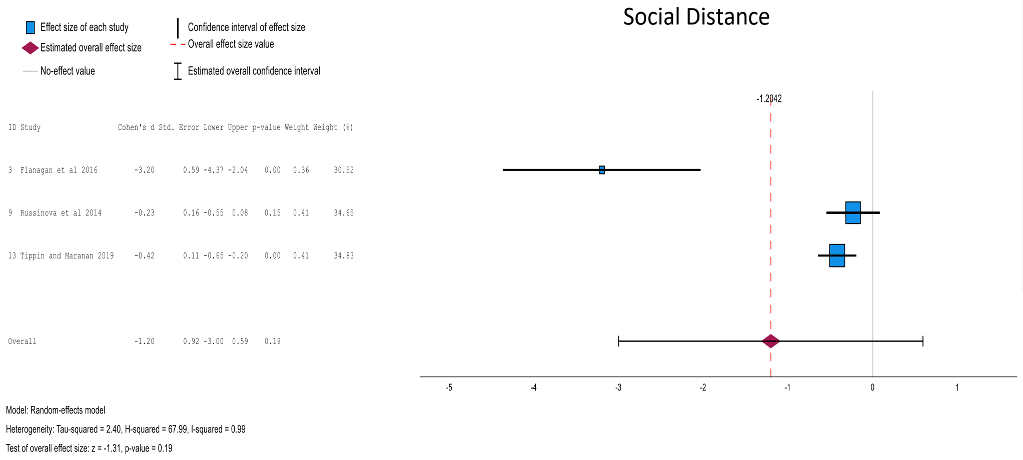

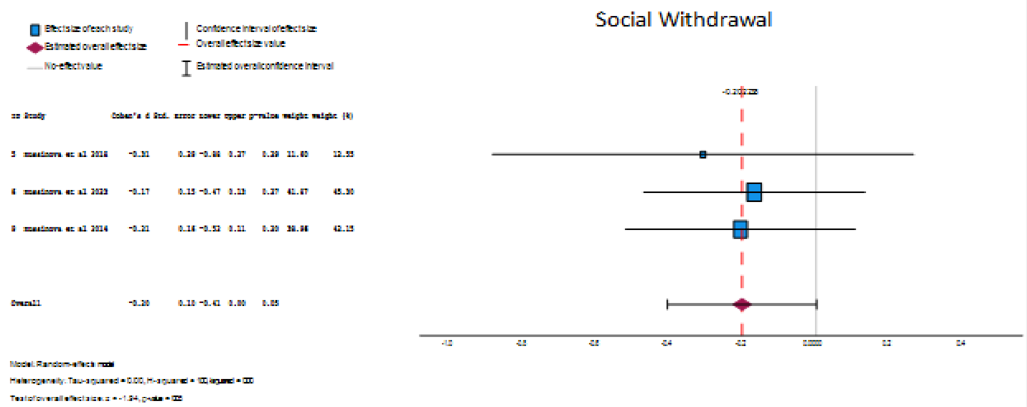

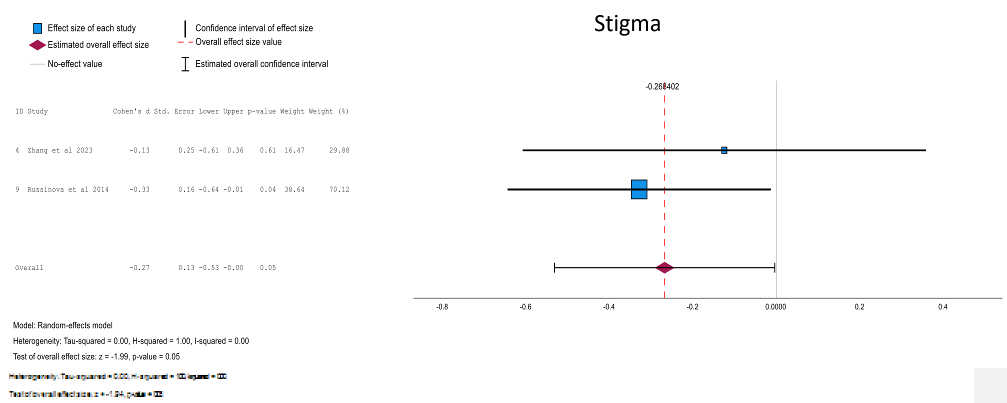

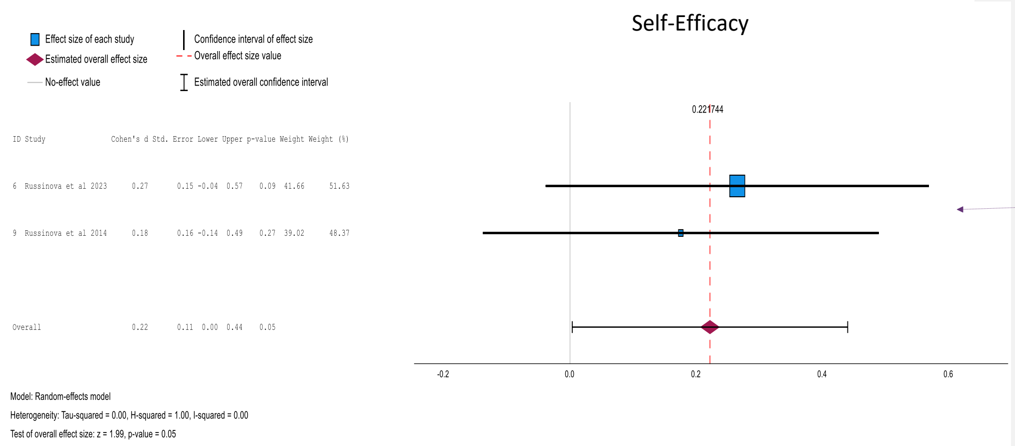

Formatted: Justified, Space Before: 4 pt, After: 10 pt, Line spacing: Double

Formatted: Normal

Effect of Photovoice on the healthcare providers of PLWMI

Formatted: Indent: First line: 0"

One paper utilized to extract data on photovoice intervention generated effect size for perception of danger and social distancing scale, therefore a meta-analysis could not be conducted for both outcomes. The reported effect size of photovoice on perception of danger in healthcare providers was -0.43(95% CI, -0.78 to -0.07). Healthcare providers that used photovoice had a 43% reduction in perception of danger relative to the control group. Additionally, the paper compared the effect of photovoice to an intervention based on didactic videos. The didactic videos showed a smaller effect on perception of danger in healthcare providers -0.01(-0.35 to 0.36). The difference between the effect of photovoice, compared to the effect of didactic videos on perception of danger was also found to be statistically significant (p=0.02). Furthermore, photovoice had -8.85 (95% CI, -13.85 to -3.84) effect size on social distance scale of healthcare providers. With healthcare providers, photovoice had 885% reduction in social distancing scale relative to the control group who never used the intervention. Didactic videos also had a smaller effect on this outcome, compared to photovoice (-5.90; -10.95 to -0.86). However, the difference between the effect of photovoice and the effect of didactic videos was not statistically significant (p=0.23). Table 4 Table 3 presents the detailed pre-calculated effect size measures for the studies exploring caregivers.

Formatted: Normal

Table 4: Pre-calculated Effect Size of photovoice addressing outcomes of healthcare providers of PLWMI.

Outcome	Study ID	Effect size	95% C I	p-value

			Lower	Upper	
Perception of Danger	Tergesen et al 2021	-0.43	-0.78	-0.07	0.02
Social Distance Scale	Tergesen et al 2021	-8.85	-13.85	-3.84	0.23

Formatted: Normal

Formatted: Normal

Formatted: Indent: First line: 0.5"

Formatted: Justified, Space Before: 4 pt, After: 10 pt, Line spacing: Double

Discussion

The results of this meta-analysis suggest that photovoice is an effective tool for reducing stigma negative mental health and stigma outcomes in PLWMI and improving positive outcomes. The results of this meta-analysis suggest that photovoice is an effective tool for reducing stigma in PLWMI. Conceptually, this is interlinked with synthesised outcome measuress in this meta-analysis, including recovery, self-efficacy, and stigma resistance. However, debates among researchers on the notion of recovery continue [542,543], with two prominent perspectives: personal and functional recovery [544].

Traditional mental health interventions such as clinical staging, a deficit-based strategy that aims to mitigate outcomes of mental illness for achieving functional recovery have been effective in reducing clinical symptoms [545,546]. However, such interventions have been criticised for being reductionistic, and not focusing on the perspective of the PLWMI regarding the meaning of recovery [542]. In contrast, photovoice represents a personal recovery-oriented approach that emphasises strength-based methods [547]. Strength-based approaches focus on increasing the resilience in PLWMI through the key characteristics of personal recovery including acceptance, hope, personal distinctiveness, agency/autonomy, social cohesion, and engagement [548].

Similarly, the efficacy of adoptingon of participatory research methods such as photovoice by PLWMI within recovery-oriented mental health have has been advocated [549-651]. This is facilitated by the sense of functionality photovoice provides, which promotes self-esteem and efficacy, as well as social dimensions of recovery by reducing isolation, fostering community engagement, and increasing the individual's sense of self-worth [549]. This can be seen in a quasi-experimental study done with participants from a psychosocial rehabilitation centre which highlights that photovoice facilitates recovery-related outcomes such as empowerment, positive sense of identity, and community integration, and therefore suggests its incorporation into recovery-oriented services of PLWMI [652]. Furthermore, such participatory research enables in-depth communication with the healthcare

Commented [MOU1]: Cannot find these references…

Commented [AU2R1]:

providers, wherein strength-based experiences beyond living with mental illness can be expressed, such as their interests and contributions to society, which has been shown to significantly reduce healthcare provider stigma, and inadvertently increase quality of care, and chance of recovery [63~~10~~].

In contrast~~Additionally~~, stigma resistance, the ability to challenge mental health stigma, stereotypes, and public prejudice at the personal, peer, and societal level [6~~5~~4], was dec~~was seen to be inc~~reased in this study following the administration of photovoice intervention. Perhaps, this reduction in stigma resistance is due to the short duration of studies analysed for this category because the photovoice intervention increases participants' sensitivity to stigmatising attitudes in the short term [65]. The study recommended that researchers should be aware of the initial phase of stereotype agreement to design efficacious interventions intended to mitigate self-stigma. However, further research revealed that Photovoice ~~was found to~~ fosters empowerment and self-identity, which posed a protective barrier against internalis~~z~~ing stigma and discrimination [11]. Likewise, its ability to build resilience, which is effective in mitigating internalised and self-stigma in vulnerable populations has been evidenced [66~~55~~-69~~58~~]. Consequently, increased resilience leads to an overall sense of empowerment for the PLWMI ensuring increased confidence, fostering engagement and connectedness within communities [5~~4~~7].

Also, self-efficacy, which can be described as the ability of PLWMI to cope and thrive with their mental illness [70~~59~~], was shown to be enhanced through the influence of photo-elicitation programs, in building sustained sense of self-worth within themselves and engagement with their community [71~~60~~,72~~61~~]. This is evident in a community-based participatory action research with participants from a community mental health institution, where they expounded on the inherent challenges in executing activities of daily living, especially regarding healthy lifestyle choices, due to the inability of set measures to adequately cater to their needs [73~~62~~]. The participants elaborated that photovoice availed an opportunity to be consulted for what they needed to lead healthier lives and facilitated co-creation of strategies with their mental health nurses which enabled successful execution

of healthier behaviours [7362]. This offers insight to the challenges of living with mental illness and empowers PLWMI to be advocates for addressing their unmet needs, which promotes a sense of purpose and achievement [7059].

Furthermore, exposure to photovoice intervention significantly reduced depression among PLWMIparticipants in the current study. This relates to the findings of a qualitative study among rural adolescents [15/17 years old] with depression and suicidal tendencies in northeast America who experienced higher sense of self-worth and empowerment after photovoice intervention [7463]. Likewise, the current meta-analysis aligns with the finding of a study in Nepal among twenty-seven 27-49 years old49-year-old rural women with self-reported depression, as measured by the Beck Depression Inventory (BDI), which was found to be significantly reduced after exposure to photovoice intervention [7564].

The current meta-analysis also highlights the potential benefits of photovoice intervention in reducing social withdrawal and angerin PLWMI. This finding is consistent with a previous qualitative study conducted among individuals with mental health issues in a mental health club in Hawaii, which identified photovoice as a helpful tool in fostering self-appraisal and inner empowerment, leading to improved social support [7665].

Concomitantly, the results also suggest that photovoice is effective for reducing social distancing, and perception of danger, and anger from healthcare providers towards PLWMI. The visual narratives created can serve as a powerful medium for reducing stereotypes and foster empathy among the HCPspublic [7766]. Importantly, social distancing from healthcare providers towards PLWMI can intensify the is a by-product of the stigma experienced by PLWMI in this population [7867]. Two perspectives worth considering are the contact-based and humanistic approaches.

One of Allport’s seminal criteria for the nature of contact in PLWMI is the perception of equal status between the contact and the receiver [5747,7968]. Traditional contact-based approaches

however disregard the inherent inequality in social status between healthcare providers and PLWMI [5~~4~~7], explaining why this form of contact is ineffective or produces only short-term effects [80~~69~~,81~~70~~]. Photovoice, however, focuses on humanistic approaches, emphasizing empathy, dignity, and respect towards the challenges experienced by PLWMI [82~~71~~]. In this context, health professionals can relate with the experiences of PLWMI, explaining its remarkable effect on mental health and~~outcomes of~~ stigma-related outcomes such as~~and~~ social distancing, anger, and perception of danger within this population [82~~71~~]. Consequently, the balance of power shifts from a feeling of "power over" inherent in healthcare professionals to "power within" in PLWMI [83~~72~~].

Implications for policy and practice and limitations of the study

This systematic review has explored the effectiveness of photovoice on mental health outcomes in ~~among~~ PLWMI and their healthcare providers. Building from the findings, mental health services should consider incorporating photovoice as a personal recovery-oriented tool as opposed to emphasis on traditional functional recovery, acknowledging its role in fostering empowerment and reducing stigma. Policies that encourage participatory research methods should be implemented, emphasising communication between PLWMI and healthcare providers. The adoption of these measures can contribute to a more holistic and individualized approach to mental health care.

Additionally, the current meta-analysis was limited based on heterogeneity due to the small number of studies exploring certain outcomes. Three~~wo~~ outcomes, anger (0.93), perception of danger (0.96), and ~~and~~ social distance (0.99) demonstrated high heterogeneity, which was attributed to the limited number of included studies ~~resulted~~ in the meta-analysis for these outcomes [84~~73~~]. While the reported statistic, I^2, provides an estimate, it does not eradicate the uncertainty that arises from having a limited number of studies in a meta-analysis [85~~74~~]. Consequently, it is important to interpret the heterogeneity encountered in both outcomes with caution. Nonetheless, the majority of the included studies were of moderate quality, with one study of high quality, and two studies having low quality.

Future RCTs should therefore consider exhausting all outcomes of mental illness, extending to outcomes not included in this meta-analysis. As ~~the~~ mental healthcare continues to evolve, further research and the successful implementation of these strategies are essential for promoting positive mental health outcomes for diverse populations.

Conclusion

Overall, it can be deduced that ~~there is sufficient evidence to reject the null hypothesis that~~ photovoice does ~~not~~ have a significant ~~significant~~ effect on improving the mental health outcomes of PLWMI. Conversely, the findings suggest that photovoice reduces the positive stigma-related outcomes in PLWMI, such as stigma resistance in the short term. Furthermore, photovoice significantly reduces the negative mental health and stigma-related outcomes in HCPs that have consequences for the mental health of PLWMI. ~~The second null hypothesis that photovoice does not have a significant effect on the behaviours of HCP towards PLWMI is also rejected.~~ Therefore, it can be concluded that photovoice improves the mental health outcomes of PLWMI and has a positive effect on the mental health and stigma-related behaviours of HCPs~~healthcare providers~~ towards PLWMI. Therefore, the effect of photovoice on stigma resistance warrants further research.

Formatted: Not Highlight

Formatted: Not Highlight

Formatted: Not Highlight

Formatted: Not Highlight

Formatted: Not Highlight

Formatted: Not Highlight

Formatted: Not Highlight

Formatted: Not Highlight

Formatted: Not Highlight

Formatted: Not Highlight

~~Acknowledgments~~Acknowledgements

The authors acknowledge See Me Scotland for providing funds and logistics support for this project.

Funding

This work was supported by See Me Scotland (URL: https://www.seemescotland.org/). There was no specific grant number associated with the grant. JW was the specific recipient of the award.

CN, WO, and CS received compensation. “The funders had no role in study design, data collection and analysis, decision to publish, or preparation of the manuscript.”

Competing interests

The authors have read the journal’s policy and hereby declare no financial or non-financial conflicts of interest.

Author contributions

AA and JW were involved in the conception, design, project management, and supervision of the review. WO, CS, and CN designed and conducted the searches and screened retrieved records for inclusion. WO, CS, AA and AA extracted data from primary studies included for meta-analyses, checked by JW and UA. WO and CS performed quality assessment, with differences to be reconciled by AA, AA and UA. AA, AA and UA conducted the meta-analyses, with AA validating the final results of the meta-analyses. UA drafted the manuscript, after which WO, CS, JW, AA and AA contributed to commenting on drafts and suggesting any revisions. All authors approved the final version and verified their ~~individual~~ contributions.

References

1. Han CS, Oliffe JL. Photovoice in mental illness research: A review and recommendations. Health Lond Engl 1997 [Internet]. 2016 Mar [cited 2023 May 19];20[2]:110–26. Available from: https://doi.org/10.1177/1363459314567790

Formatted: Indent: Left: -0.26", Hanging: 0.26"

2. Leyva-Moral JM, Gómez-Ibáñez R, San Rafael S, Guevara-Vásquez G, Aguayo-González M. Nursing students' satisfaction with narrative photography as a method to develop empathy towards people with Hiv: A mixed-design study. Nurse Educ Today [Internet]. 2021 Jan 1 [cited 2024 Jan 21];96:104646. Available from: https://doi.org/10.1016/j.nedt.2020.104646

3. Husin SS, Ab Rahman AA, Mukhtar D. The symbolic interactionism theory: a systematic literature review of current research. Int J Mod Trends Soc Sci [Internet]. 2021 Sep 15 [cited 2024 Jan 21];4[17]:113–26. Available from: DOI: 10.35631/IJMTSS.417010

4. Halvorsrud K, Eylem O, Mooney R, Haarmans M, Bhui K. Identifying evidence of the effectiveness of photovoice: a systematic review and meta-analysis of the international healthcare literature. J Public Health [Internet]. 2022 Sep 1 [cited 2023 May 24];44[3]:704–12. Available from: https://doi.org/10.1093/pubmed/fdab074

5. Elraz H. Identity, mental health and work: How employees with mental health conditions recount stigma and the pejorative discourse of mental illness. Hum Relat [Internet]. 2018 May 1 [cited 2024 Jan 21];71[5]:722–41. Available from: https://doi.org/10.1177/0018726717716752

6. Baron EC, Rathod SD, Hanlon C, Prince M, Fedaku A, Kigozi F, et al. Impact of district mental health care plans on symptom severity and functioning of patients with priority mental health conditions: the Programme for Improving Mental Health Care [PRIME] cohort protocol. BMC Psychiatry [Internet]. 2018 Mar 6 [cited 2024 Jan 21];18[1]:61. Available from: https://doi.org/10.1186/s12888-018-1642-x

7. Sestito SF, Rodriguez KL, Saba SK, Conley JW, Mitchell MA, Gordon AJ. Homeless Veterans' Experiences with Substance Use, Recovery, and Treatment through Photo Elicitation. Subst Abuse [Internet]. 2017 Oct 1 [cited 2024 Jan 21];38[4]:422–31. Available from: https://doi.org/10.1080/08897077.2017.1356422

8. True G, Rigg KK, Butler A. Understanding Barriers to Mental Health Care for Recent War Veterans Through Photovoice. Qual Health Res [Internet]. 2015 Oct [cited 2023 Nov 5];25[10]:1443–55. Available from: http://journals.sagepub.com/doi/10.1177/1049732314562894

9. Kopera M, Suszek H, Bonar E, Myszka M, Gmaj B, Ilgen M, et al. Evaluating Explicit and Implicit Stigma of Mental Illness in Mental Health Professionals and Medical Students. Community Ment Health J. 2015; 51(5):628-634 Available from: https://doi.org/10.1007/s10597-014-9796-6 doi:10.1007/s10597-014-9796-6.

Formatted: Normal, Indent: Hanging: 0.26"

10~~9~~. Kohrt BA, Jordans MJD, Turner EL, Rai S, Gurung D, Dhakal M, et al. Collaboration With People With Lived Experience of Mental Illness to Reduce Stigma and Improve Primary Care Services: A Pilot Cluster Randomized Clinical Trial. JAMA Netw Open [Internet]. 2021 Nov 3 [cited 2023 Jun 2];4[11]:e2131475. Available from: https://doi.org/10.1001/jamanetworkopen.2021.31475

Formatted: Indent: Left: -0.26", Hanging: 0.26"

11~~0~~. Mizock L, Russinova Z, DeCastro S. Recovery narrative photovoice: Feasibility of a writing and photography intervention for serious mental illnesses. Psychiatr Rehabil J [Internet]. 2015 Sep [cited 2023 Nov 5];38[3]:279–82. Available from: http://doi.apa.org/getdoi.cfm?doi=10.1037/prj0000111

12~~1~~. Russinova Z, Gidugu V, Bloch P, Restrepo-Toro M, Rogers ES. Empowering individuals with psychiatric disabilities to work: Results of a randomized trial. Psychiatr Rehabil J. 2018;41:196–207. Available from: https://doi.org/10.1037/prj0000303

13~~2~~. Becker K, Reiser M, Lambert S, Covello C. Photovoice: Conducting Community-Based Participatory Research and Advocacy in Mental Health. J Creat Ment Health [Internet]. 2014 Apr 3 [cited 2023 Apr 8];9[2]:188–209. Available from: https://doi.org/10.1080/15401383.2014.890088

14~~3~~. Yanos PT, Lucksted A, Drapalski AL, Roe D, Lysaker P. Interventions targeting mental health self-stigma: A review and comparison. Psychiatr Rehabil J. 2015;38[2]:171–8. Available from: https://doi.org/10.1037/prj0000100

15~~4~~. Nykiforuk CIJ. Engaging patients in research using photovoice methodology. CMAJ [Internet]. 2021 Jul 12 [cited 2023 Apr 6];193[27]:E1050–1. Available from: https://doi.org/10.1503/cmaj.210963

16~~5~~. Creighton G, Oliffe JL, Ferlatte O, Bottorff J, Broom A, Jenkins EK. Photovoice Ethics: Critical Reflections From Men's Mental Health Research. Qual Health Res [Internet]. 2018 Feb [cited 2023 Jun 15];28[3]:446–55. Available from: DOI:10.1177/1049732317729137

17. Woda A, Haglund K, Belknap R, Cleek E. Photovoice: A Research Method and Intervention to Engage Older Adults. Journal of gerontological nursing . 2018 Jul 1, ; 44(7):43-49 Available from:https://www.ncbi.nlm.nih.gov/pubmed/29969139 doi:10.3928/00989134-20180614-07.

18. Call-Cummings M, Hauber-Özer M, Byers C, Mancuso GP. The power of/in Photovoice. International journal of research & method in education . 2019 Aug 8, ; 42(4):399-413 Available from:https://www.tandfonline.com/doi/abs/10.1080/1743727X.2018.1492536 doi:10.1080/1743727X.2018.1492536.

19. Christensen MC. Using Photovoice to Address Gender-Based Violence: A Qualitative Systematic Review. Trauma Violence Abuse . 2019 Oct ; 20(4):484-497 doi:10.1177/1524838017717746.

20. Wang C, Burris MA. Photovoice: Concept, Methodology, and Use for Participatory Needs Assessment. Health Educ Behav [Internet]. 1997 Jun [cited 2023 Apr 6];24[3]:369–87. Available from: http://journals.sagepub.com/doi/10.1177/109019819702400309

21. Carlson ED, Engebretson J, Chamberlain RM. Photovoice as a Social Process of Critical Consciousness. Qualitative health research . 2006 Jul ; 16(6):836-852 Available from:https://journals.sagepub.com/doi/full/10.1177/1049732306287525 doi:10.1177/1049732306287525.

22. Catalani C, Minkler M. Photovoice: A Review of the Literature in Health and Public Health. Health Education & Behavior . 2010 Jun 1, ; 37(3):424-451 Available from:https://www.jstor.org/stable/45056201 doi:10.1177/1090198109342084.

23. Liebenberg L. Thinking Critically About Photovoice. International journal of qualitative methods . 2018 Dec 1, ; 17(1):160940691875763 Available

Formatted: Indent: Hanging: 0.26"

from:https://journals.sagepub.com/doi/full/10.1177/1609406918757631 doi:10.1177/1609406918757631.

24. Hergenrather KC, Rhodes SD, Cowan CA, Bardhoshi G, Pula S. Photovoice as Community-Based Participatory Research: A Qualitative Review. American journal of health behavior . 2009 Nov 1, ; 33(6):686-698 Available from:https://www.ingentaconnect.com/content/png/ajhb/2009/00000033/00000006/art00006 doi:10.5993/AJHB.33.6.6.

25. Jacobs R. Investigating Patient Outcome Measures in Mental Health.: Centre for Health Economics, University of York; 2009.

26. Brohan E, Slade M, Clement S, Thornicroft G. Experiences of mental illness stigma, prejudice and discrimination: a review of measures. BMC Health Services Research . 2010; 10(1):80 Available from: https://doi.org/10.1186/1472-6963-10-80 doi:10.1186/1472-6963-10-80.

16. Raccichini M, Biancalani G, Franchini L, Varani S, Ronconi L, Testoni I. Death education and photovoice at school: A workshop with Italian high school students. Death Stud [Internet]. 2023 Mar 16 [cited 2024 Jan 21];47[3]:279–86. Available from: https://doi.org/10.1080/07481187.2022.2052206

17. Popay J, Roberts H, Sowden A, Petticrew M, Arai L, Rodgers M, et al. Guidance on the Conduct of Narrative Synthesis in Systematic Reviews. 2006;

2718. Uman LS. Systematic Reviews and Meta-Analyses. J Can Acad Child Adolesc Psychiatry [Internet]. 2011 Feb [cited 2023 Sep 27];20[1]:57–9. Available from: https://www.ncbi.nlm.nih.gov/pmc/articles/PMC3024725/

2819. Tawfik GM, Dila KAS, Mohamed MYF, Tam DNH, Kien ND, Ahmed AM, et al. A step by step guide for conducting a systematic review and meta-analysis with simulation data. Trop Med Health [Internet]. 2019 Aug 1 [cited 2023 Sep 27];47[1]:46. Available from: https://doi.org/10.1186/s41182-019-0165-6

290. Mikolajewicz N, Komarova SV. Meta-Analytic Methodology for Basic Research: A Practical Guide. Front Physiol [Internet]. 2019 [cited 2023 Sep 27];10. Available from: https://doi.org/10.3389/fphys.2019.00203

3021. Paul J, Barari M. Meta-analysis and traditional systematic literature reviews—What, why, when, where, and how? Psychol Mark [Internet]. 2022 [cited 2023 Sep 27];39[6]:1099–115. Available from: https://doi.org/10.1002/mar.21657

3122. Lee YH. Strengths and Limitations of Meta-Analysis. Korean J Med [Internet]. 2019 Oct 1 [cited 2023 Sep 27];94[5]:391–5. Available from: https://doi.org/10.3904/kjm.2019.94.5.391

3223. Haidich AB. Meta-analysis in medical research. Hippokratia [Internet]. 2010 Dec [cited 2023 Sep 27];14[Suppl 1]:29–37. Available from: https://www.ncbi.nlm.nih.gov/pmc/articles/PMC3049418/

3324. Hariton E, Locascio JJ. Randomised controlled trials—the gold standard for effectiveness research. BJOG Int J Obstet Gynaecol [Internet]. 2018 Dec [cited 2023 Sep 28];125[13]:1716. Available from: https://doi.org/10.1111/1471-0528.15199

Formatted: Font: Bold

Formatted: Indent: Left: -0.26", Hanging: 0.26"

34~~25~~. Clyne B, Boland F, Murphy N, Murphy E, Moriarty F, Barry A, et al. Quality, scope and reporting standards of randomised controlled trials in Irish Health Research: an observational study. Trials [Internet]. 2020 Jun 8 [cited 2023 Sep 28];21[1]:494. Available from: https://doi.org/10.1186/s13063-020-04396-x

~~26. Wang C, Burris MA. Photovoice: Concept, Methodology, and Use for Participatory Needs Assessment. Health Educ Behav [Internet]. 1997 Jun [cited 2023 Apr 6];24[3]:369–87. Available from: http://journals.sagepub.com/doi/10.1177/109019819702400309~~

35~~27~~. Bearman M, Smith C, Carbone A, Slade S, Baik C, Hughes-Warrington M, et al. Systematic review methodology in higher education. High Educ Res Dev. 2012 Oct 1;31. Available from: https://doi.org/10.1080/07294360.2012.702735

36~~28~~. Paez A. Gray literature: An important resource in systematic reviews. J Evid-Based Med [Internet]. 2017 [cited 2023 Jun 16];10[3]:233–40. Available from: https://onlinelibrary.wiley.com/doi/abs/10.1111/jebm.12266

37~~29~~. Adams J, Hillier-Brown FC, Moore HJ, Lake AA, Araujo-Soares V, White M, et al. Searching and synthesising 'grey literature' and 'grey information' in public health: critical reflections on three case studies. Syst Rev [Internet]. 2016 Sep 29 [cited 2023 Jul 28];5[1]:164. Available from: https://doi.org/10.1186/s13643-016-0337-y

38~~0~~. Almeida CPB de, Goulart BNG de. How to avoid bias in systematic reviews of observational studies. Rev CEFAC [Internet]. 2017 Aug [cited 2023 Jul 11];19:551–5. Available from: https://doi.org/10.1590/1982-021620171941117

39~~1~~. Haddaway NR, Collins AM, Coughlin D, Kirk S. The Role of Google Scholar in Evidence Reviews and Its Applicability to Grey Literature Searching. PLOS ONE [Internet]. 2015 Sep 17 [cited 2023 Jun 13];10[9]:e0138237. Available from: https://doi.org/10.1371/journal.pone.0138237

40~~32~~. Kellermeyer L, Harnke B, Knight S. Covidence and Rayyan. J Med Libr Assoc JMLA [Internet]. 2018 Oct [cited 2023 Dec 1];106[4]:580–3. Available from: https://doi.org/10.5195%2Fjmla.2018.513

41~~33~~. Vu-Ngoc H, Elawady SS, Mehyar GM, Abdelhamid AH, Mattar OM, Halhouli O, et al. Quality of flow diagram in systematic review and/or meta-analysis. PLOS ONE [Internet]. 2018 Jun 27 [cited 2023 Jul 20];13[6]:e0195955. Available from: https://doi.org/10.1371/journal.pone.0195955

42~~34~~. Page MJ, McKenzie JE, Bossuyt PM, Boutron I, Hoffmann TC, Mulrow CD, et al. The PRISMA 2020 statement: an updated guideline for reporting systematic reviews. BMJ [Internet]. 2021 Mar 29 [cited 2022 Dec 30];372:n71. Available from: https://doi.org/10.1136/bmj.n71

43~~35~~. dos Santos WM, Secoli SR, Püschel VA de A. The Joanna Briggs Institute approach for systematic reviews. Rev Lat Am Enfermagem [Internet]. 2018 Nov 14 [cited 2023 Sep 29];26:e3074. Available from: https://doi.org/10.1590%2F1518-8345.2885.3074

44~~36~~. Centre for Reviews and Dissemination. CRD's guidance for undertaking reviews in healthcare. 3. ed. York: York Publ. Services; 2009. 281 p. [Systematic reviews].

45~~37~~. Buchter RB, Weise A, Pieper D. Development, testing and use of data extraction forms in systematic reviews: a review of methodological guidance. BMC Med Res Methodol [Internet]. 2020 Oct 19 [cited 2023 Jul 20];20[1]:259. Available from: https://doi.org/10.1186/s12874-020-01143-3

4638. Bown MJ, Sutton AJ. Quality Control in Systematic Reviews and Meta-analyses. Eur J Vasc Endovasc Surg [Internet]. 2010 Nov 1 [cited 2023 Jul 29];40[5]:669–77. Available from: https://doi.org/10.1016/j.ejvs.2010.07.011

47. Harrer M, Cuijpers P, Furukawa TA, Ebert DD. Doing meta-analysis with R.First edition ed. Boca Raton ; London ; New York: CRC Press; 2022.

4839. Effective Public Healthcare Panacea Project. Effective Public Healthcare Panacea Project. 2009 [cited 2023 Dec 1]. Quality Assessment Tool for Quantitative Studies. Available from: https://www.ephpp.ca/quality-assessment-tool-for-quantitative-studies/

490. Meyvis T, Van Osselaer SMJ. Increasing the Power of Your Study by Increasing the Effect Size. J Consum Res [Internet]. 2018 Feb 1 [cited 2024 Jan 21];44[5]:1157–73. Available from: https://doi.org/10.1093/jcr/ucx110

50. Goulet-Pelletier J, Cousineau D. A review of effect sizes and their confidence intervals, part I: the Cohen's d family. The Quantitative Methods for Psychology . 2018 Dec 1, ; 14(4):242-265 Available from:https://doi.org/10.20982/tqmp.14.4.p242 doi:10.20982/tqmp.14.4.p242.

541. Ebert MH Pim Cuijpers, Toshi Furukawa, David. Doing Meta-Analysis with R: A Hands-On Guide. New York: Chapman and Hall/CRC; 2021. 500 p.

542. Treichler EBH, Li F, O'Hare M, Evans EA, Johnson JR, Spaulding WD. Psychosocial and functional contributors to personal recovery in serious mental illness. Journal of Mental Health . 2019; 28[4]:427-435 Available from: https://doi.org/10.1080/09638237.2018.1521932

543. Jahn DR, Leith J, Muralidharan A, Brown CH, Drapalski AL, Hack S, et al. The Influence of Experiences of Stigma on Recovery: Mediating Roles of Internalized Stigma, Self-Esteem, and Self-Efficacy. Psychiatric rehabilitation journal. 2020 Jun 1, ; 43[2]:97-105 Available from: https://psycnet.apa.org/doi/10.1037/prj0000377

544. Slade M, Amering M, Farkas M, Hamilton B, O'Hagan M, Panther G, et al. Uses and abuses of recovery: implementing recovery-oriented practices in mental health systems. World Psychiatry [Internet]. 2014 [cited 2024 Jan 21];13[1]:12–20. Available from: https://onlinelibrary.wiley.com/doi/abs/10.1002/wps.20084

545. Capon W, Hickie IB, Varidel M, Prodan A, Crouse JJ, Carpenter JS, et al. Clinical staging and the differential risks for clinical and functional outcomes in young people presenting for youth mental health care. BMC Medicine . 2022; 20[1]:479 Available from:https://doi.org/10.1186/s12916-022-02666-w

546. Hickie IB, Scott EM, Cross SP, Iorfino F, Davenport TA, Guastella AJ, et al. Right care, first time: a highly personalised and measurement-based care model to manage youth mental health. Med J Aust . 2019; 211:S3-S46 Available from:https://doi.org/10.5694/mja2.50383

547. Ungar T, Knaak S, Szeto AC. Theoretical and Practical Considerations for Combating Mental Illness Stigma in Health Care. Community Ment Health J [Internet]. 2016 Apr 1 [cited 2024 Jan 21];52[3]:262–71. Available from: -https://doi.org/10.1007/s10597-015-9910-4

548. Cripps L, Deyell Hood C. Recovery and Mental Health: Exploring the Basic Characteristics of Living Well with Mental Illness. Ther Recreation J [Internet]. 2020 Jun 2 [cited 2024 Jan 21];54[2]. Available from: https://doi.org/10.18666/TRJ-2020-V54-I2-9948

Formatted: Normal, Indent: Hanging: 0.26"

Formatted: Indent: Left: -0.26", Hanging: 0.26"

Formatted: Normal, Indent: Hanging: 0.26"

Formatted: Indent: Left: -0.26", Hanging: 0.26"

549. Cabassa LJ, Nicasio A, Whitley R. Picturing Recovery: A Photovoice Exploration of Recovery Dimensions Among People With Serious Mental Illness. Psychiatr Serv Wash DC [Internet]. 2013 Sep 1 [cited 2023 May 19];64[9]:10.1176/appi.ps.201200503. Available from: https://doi.org/10.1176%2Fappi.ps.201200503

6050. Vansteenkiste T, Morrens M, Westerhof GJ. Images of Recovery: A PhotoVoice Study on Visual Narratives of Personal Recovery in Persons with Serious Mental Illness. Community Ment Health J [Internet]. 2021 Aug [cited 2023 May 24];57[6]:1151–63. Available from: https://doi.org/10.1007/s10597-020-00746-w

651. Flanagan EH, Buck T, Gamble A, Hunter C, Sewell I, Davidson L. 'Recovery Speaks': A Photovoice Intervention to Reduce Stigma Among Primary Care Providers. Psychiatr Serv. 2016 May;67[5]:566–9. Available from: https://doi.org/10.1176/appi.ps.201500049

652. Mizock L, Russinova Z, DeCastro S. Recovery Narrative Photovoice: Feasibility of a Writing and Photography Intervention for Serious Mental Illnesses. Psychiatr Rehabil J . 2015; 38[3]:279-282 doi:10.1037/prj0000111

653. Boyd Ritsher J, Otilingam PG, Grajales M. Internalized stigma of mental illness: psychometric properties of a new measure. Psychiatry Res [Internet]. 2003 Nov 1 [cited 2024 Jan 21];121[1]:31–49. Available from: https://doi.org/10.1016/j.psychres.2003.08.008

Formatted: Normal, Indent: Hanging: 0.26"

654. Thoits PA. Resisting the Stigma of Mental Illness. Soc Psychol Q [Internet]. 2011 Mar 1 [cited 2024 Jan 21];74[1]:6–28. Available from: https://doi.org/10.1177/0190272511398019

Formatted: Indent: Left: -0.26", Hanging: 0.26"

65. CORRIGAN PW, WATSON AC, BARR L. The Self–Stigma of Mental Illness: Implications for Self–Esteem and Self–Efficacy. Journal of Social and Clinical Psychology . 2006 Oct 1, ; 25(8):875-884 Available from:https://search.proquest.com/docview/224857864 doi:10.1521/jscp.2006.25.8.875.

Formatted: Normal, Indent: Hanging: 0.26"

6565. Adeboye A, Ross MW, Wilkerson MJ, Springer A, Ahaneku H, Yusuf RA, et al. Resilience Factors as a Buffer against the Effects of Syndemic Conditions on HIV Risk and Infection among Tanzanian MSM. Journal of Health Education Research & Development . 2017; 5[3] Available from: https://doi.org/10.4172/2380-5439.1000232.

Formatted: Indent: Left: -0.26", Hanging: 0.26"

6576. Dow DE, Mmbaga BT, Turner EL, Gallis JA, Tabb ZJ, Cunningham CK, et al. Building resilience: a mental health intervention for Tanzanian youth living with HIV. AIDS Care . 2018; 30:12-20 Available from:https://doi.org/10.1080/09540121.2018.1527008

6857. Macpherson H, Hart A, Heaver B. Building resilience through group visual arts activities: Findings from a scoping study with young people who experience mental health complexities and/or learning difficulties. Journal of Social Work . 2016; 16[5]:541-560 Available from:https://doi.org/10.1177/1468017315581772

6958. McAllister M, Knight BA, Hasking P, Withyman C, Dawkins J. Building resilience in regional youth: Impacts of a universal mental health promotion programme. Int J Mental Health Nurs . 2018; 27[3]:1044-1054 Available from:https://doi.org/10.1111/inm.12412

7059. Werremeyer AB, Aalgaard-Kelly G, Skoy E. Using Photovoice to explore patients' experiences with mental health medication: A pilot study. Ment Health Clin [Internet]. 2016 Jan 1 [cited 2023 May 24];6[3]:142–53. Available from: https://doi.org/10.9740/mhc.2016.05.142

71~~60~~. Russinova Z, Rogers ES, Gagne C, Bloch P, Drake KM, Mueser KT. A randomized controlled trial of a peer-run antistigma photovoice intervention. Psychiatr Serv. 2014;65[2]:242–6. Available from: https://doi.org/10.1176/appi.ps.201200572

72~~61~~. Russinova Z, Link to external site this link will open in a new window, Gidugu V, Rogers ES, Legere L, Bloch P. Fostering the community participation of individuals with psychiatric disabilities: Effectiveness of a new peer-led photovoice-based intervention. Psychiatr Rehabil J [Internet]. 2023 Mar 30 [cited 2023 May 30]; Available from: DOI:10.1037/prj0000540

73~~62~~. Edmondson AJ, Borthwick R, Hughes E, Lucock M. Using photovoice to understand and improve healthy lifestyles of people diagnosed with serious mental illness. J Psychiatr Ment Health Nurs [Internet]. 2022 [cited 2023 May 24];29[5]:676–87. Available from: https://onlinelibrary.wiley.com/doi/abs/10.1111/jpm.12863

74~~63~~. Dempsey JS. Using Photovoice as a Community-Based Participatory Research Method to Identify Perceived Risk and Protective Factors for Depression in Rural Adolescents. Int J Nurs Clin Pract [Internet]. 2016 Aug 4 [cited 2023 May 24];3[1]. Available from: http://dx.doi.org/10.15344/2394-4978/2016/194

75~~64~~. MacFarlane EK, Shakya R, Berry HL, Kohrt BA. Implications of participatory methods to address mental health needs associated with climate change: 'photovoice' in Nepal. BJPsych international . 2015 May ; 12[2]:33-35 Available from:https://dx.doi.org/10.1192/S2056474000000246

76~~65~~. Agner J, Nakamura L, Botero A, Cha T, Kaukau TM. Can Photovoice foster the development of social support? Am J Community Psychol [Internet]. 2023 [cited 2024 Jan 21];71[3–4]:371–81. Available from: https://onlinelibrary.wiley.com/doi/abs/10.1002/ajcp.12649

77~~66~~. Levy B, Celen-Demirtas S, Surguladze T, Sweeney KK. Stigma and Discrimination: A Socio-Cultural Etiology of Mental Illness. The Humanistic Psychologist. 2014; 42[2]:199-214 Available from: https://doi.org/10.1080/08873267.2014.893513

78~~67~~. Munir K, Oner O, Kerala C, Rustamov I, Boztas H, Juszkiewicz K, et al. Social distance and stigma towards persons with serious mental illness among medical students in five European Central Asia countries. Psychiatry Res . 2022 Mar ; 309:114409 doi:10.1016/j.psychres.2022.114409.

79~~68~~. Allport GW. The nature of prejudice. Cambridge, Mass: Addison-Wesley Pub. Co; 1954.

80~~69~~. Carrara BS, Fernandes RHH, Bobbili SJ, Ventura CAA. Health care providers and people with mental illness: An integrative review on anti-stigma interventions. Int J Soc Psychiatry . 2021 Nov ; 67[7]:840-853 doi:10.1177/0020764020985891.

81~~70~~. Jorm AF. Effect of Contact-Based Interventions on Stigma and Discrimination: A Critical Examination of the Evidence. PS . 2020; 71[7]:735-737 Available from: https://doi.org/10.1176/appi.ps.201900587

82~~71~~. Coffey J. Images as 'potentials': Feminist new materialist orientations to photovoice. Qualitative Research . 2023; 23[4]:847-865 Available from:https://doi.org/10.1177/14687941211049334

83~~72~~. Sweeney A, Filson B, Kennedy A, Collinson L, Gillard S. A paradigm shift: relationships in trauma-informed mental health services. BJPsych Adv [Internet]. 2018 Sep [cited 2024 Jan 21];24[5]:319–33. Available from: https://doi.org/10.1192/bja.2018.29

8473. Stanley TD, Doucouliagos H, Ioannidis JPA. Beyond Random Effects: When Small-Study Findings Are More Heterogeneous. Adv Methods Pract Psychol Sci [Internet]. 2022 Oct 1 [cited 2024 Jan 21];5[4]:25152459221120427. Available from: https://doi.org/10.1177/25152459221120427

8574. von Hippel PT. The heterogeneity statistic I2 can be biased in small meta-analyses. BMC Medical Research Methodology. 2015; 15[1]:35 Available from:https://doi.org/10.1186/s12874-015-0024-z

Other references

- Carlson, E. D., Engebretson, J., & Chamberlain, R. M. (2006). Photovoice as a social process of critical consciousness. *Qualitative Health Research, 16*(6), 836-852.

- Catalani, C., & Minkler, M. (2010). Photovoice: A review of the literature in health and public health. *Health Education & Behavior, 37*(3), 424-451.

- Liebenberg, L. (2018). Thinking critically about photovoice: Achieving empowerment and social change. *International Journal of Qualitative Methods, 17*(1), 1609406918757631.

- Wang, C., & Burris, M. A. (1997). Photovoice: Concept, methodology, and use for participatory needs assessment. *Health Education & Behavior, 24*(3), 369-387.

Call-Cummings, M., Hauber-Özer, M., Byers, C., & Mancuso, G. P. (2018). The power of/in Photovoice. International Journal of Research & Method in Education, 42(4), 399–413. https://doi.org/10.1080/1743727X.2018.1492536

Hergenrather, K. C., Rhodes, S. D., Cowan, C. A., Bardhoshi, G., & Pula, S. (2009). Photovoice as Community-Based Participatory Research: A Qualitative Review. American Journal of Health Behavior, 33(6), 686–698. https://doi.org/10.5993/AJHB.33.6.6

Russinova Z, Gidugu V, Bloch P, Restrepo-Toro M, Rogers ES. Empowering individuals with psychiatric disabilities to work: Results of a randomized trial. Psychiatr Rehabil J. 2018;41(3):196-207. doi:10.1037/prj0000303.

Schenker, M. B., Castañeda, X., & Rodriguez-Lainz, A. (Eds.). (2014). Migration and health: A research methods handbook. University of California Press.

Woda, A., Haglund, K., Belknap, R., & Cleek, E. (2018). Photovoice: A Research Method and Intervention to Engage Older Adults. Journal of Gerontological Nursing, 44(7), 43-49. https://doi.org/10.3928/00989134-20180614-07

(1) Brohan E, Slade M, Clement S, Thornicroft G. Experiences of mental illness stigma, prejudice and discrimination: a review of measures. BMC Health Services Research . 2010; 10(1):80 Available from: https://doi.org/10.1186/1472-6963-10-80 doi:10.1186/1472-6963-10-80.

Formatted: Bibliography, Indent: Left: -0.26", Hanging: 0.26", Line spacing: single

Formatted: Font: Bold

Formatted: Bibliography, Indent: Hanging: 0.26", Tab stops: Not at 0.86"

Formatted: Bibliography, Indent: Hanging: 0.26"

Formatted: Indent: Hanging: 0.26", Line spacing: single

Formatted: Indent: Hanging: 0.26"

Formatted: Font: 13.5 pt, Font color: Black, Ligatures: None

Formatted: Justified, Indent: Hanging: 0.26", Space Before: 4 pt, After: 10 pt

Formatted: Indent: Hanging: 0.26", Line spacing: single, Tab stops: Not at 0.86"

Formatted: Font: Not Bold

Field Code Changed

Formatted: Hyperlink, Font: Not Bold

Formatted: Font: Not Bold

Supplementary material

S1. Fig. Full search history on all databasesProQuest Central.

S1 Fig.docxS1 Fig.docx

Formatted: Default Paragraph Font, Font: Not Bold, English (United Kingdom)

S1. Table. Data Extraction Form.

S1 Table.docxS1 Table.docx

Field Code Changed

S1. Appendix. Reference list of included studies.

S1 Appendix.docxS1 Appendix.docx

Field Code Changed

S2. Appendix. PRISMA 202009 Checklist.

S2 Appendix.docx

S2 Appendix.docx

Ugochukwu Aghalu [MPH]
Glasgow Caledonian University,
Cowcaddens Road,
Glasgow, United Kingdom G4 0BA,
[UAGHAL300@caledonian.ac.uk]

The Editorial team,
PLOS GLOBAL
PUBLIC HEALTH.
[6th July, 2024]

Rebuttal letter to the reviewers

Reviewer 1:

Thank you for taking your time to thoroughly review the manuscript.

Abstract: Please follow Prisma 2020 guidelines for the reporting of systematic review abstract.

We have endeavoured to follow the PRISMA 2020 guidelines in the new abstract that we have provided in the revised manuscript.

Pg4, p2: study evaluates the impact of photovoice in mental ill health: vague and not clear based on included papers. The abstract mentions the evaluation of the effects of photovoice on 8 mental health outcomes. For clarity, it would be helpful if authors separate mental health outcomes and stigma outcomes as different.

Pg5 p2: "what is the impact of photovoice on mental health outcomes?" – Similar to above comment, it would be great if authors could modify this into the impact of photovoice on mental health and stigma outcomes of PLWMI and HCPs.

We have incorporated these great points into the new manuscript, separating outcomes into mental health and stigma outcomes.

Pg6: Search strategy- The authors need to include full search strategies used for all databases including any filters and limits used. (See Prisma guideline for reporting)- I saw that authors have included prisma 2009 checklist- this need to be referenced in the main manuscript and as there is prisma guideline/checklist for 2020, it would be great if authors updated the checklist.

The full search strategy for all databases searched have been updated. Likewise, the PRISMA checklist has been updated to 2020.

Table 1: Characteristics of included studies- it is confusing as some studies only had PLWMIs some only had HCPs while some had both as population and it is also not clear which outcomes/questionnaires were used for which population? Would be helpful if the authors added a column on population for the studies.
Another useful thing to mention in the table is the outcome measure as per your analysis. Currently the outcomes listed in the table does not match with the outcomes you report. How were the listed outcomes such as dangerousness, social desirability, empowerment, coping with stigma etc categorized into the outcomes you report on later?

The characteristics of included studies have been expanded accordingly for more comprehension. All the authors were consulted in the process of pulling and logically matching outcomes from studies to be able to carry out meta-analysis, and that is how we arrived at the outcomes for this current meta-analysis.

Pg 19, 20: The data analysis section should go into the methods section rather than the results.

This has been addressed in the new manuscript.

Pg 21: The effects of photovoice on stigma resistance and social distance of PLWMIs is not clear. Social distance is used to measure public/provider stigma towards PLWMIs – not with PLWMIs. Is this social withdrawal instead of social distance?

Also, how was stigma resistance measured- I don't see stigma resistance mentioned in any of the outcomes or questionnaires you have listed in Table 1? Is this stigma coping?

The papers listed under PLWMI outcomes of anger, recovery, social distance, etc. (Flanagan 2016, Tippin and Maranzan 2019) are targeting health workers rather than PLWMI- I am not sure how the authors assigned the health workers' outcomes to PLWMIs?

We have repeated the meta-analysis and assigned the correct papers to the correct populations in the new manuscript. We have also evidenced the measure of stigma resistance in Table 1, so we believe these points have been addressed.

Pg 25 I think there were a lot more papers on health worker stigma outcomes- as mentioned in the study characteristics (table 1), however, the authors have only described the effects of Tergesen's 2021 paper on the effect of photovoice on the healthcare provider section. In addition, the Tergesen paper mentions an increase in stigma scores among medical students after watching the psychosis photovoice video. This is not reflected in the findings of the current paper.

We have decided to remove the Tegersen's paper due to its unsuitability for meta-analysis and hone in on the outcomes that can be verifiable by conducting thorough meta-analysis. Addressing the initial point, we have re-assigned the right papers to the right population, and expanded the characteristics table to reflect populations.

On a similar note, the paper Zhang et al 2023, the study uses mindfulness-based psychoeducation as an intervention with a component of photovoice and its effects on YAIR. The author's interpretation that this is the effect of photovoice rather than the effect of mindfulness-based psychoeducation might be a bit farfetched.

Firstly, we have included a discussion in the introduction that photovoice can be utilized as both an intervention, and a method, and backed it up with relevant references.

Additionally, the paper, Zhang et al., 2023 utilised the methodology of photovoice to deliver the intervention. We refer to the appendix in the study that demonstrates that photovoice was consistently utilized over the duration of the study in all five phases of the intervention administered by the Mindfulness-Based Family Psychoeducation (MBFBE) (Arm 1). Furthermore, the detailed discussion of how the methodology of photovoice was utilized in this study to deliver the MBFBE is provided in the qualitative aspect of the study in Lo et al. (2022).

Lo HH, Liu KH, Ho WC, Lau EN, Poon MF, Lo CS, et al. Using Photovoice in a Mindfulness-Based Program to Understand the Experiences of Caregivers of Young Adults with Psychosis. International Journal of Environmental Research and Public Health. 2022; 19(23) doi:10.3390/ijerph192315461.

Overall, we thank you for the hugely insightful feedback as we have utilized this to thoroughly improve the rigour and internal validity of our meta-analysis.

Reviewer 2:

Thank you for taking your time to thoroughly review our manuscript.

A. Introduction: The authors talk about the rationale and advantages of Photovoice but more detail on the content and process of Photovoice is required – e.g. how are the participants selected, trained, how do they write stories and take their photos and how are they presented across different settings. This should help in setting up the background of the study.

We have taken our time to incorporate the expanded discussion of photovoice into the introduction section of the new manuscript.

"Mental health outcomes" – that the author describes in the abstract and rest of the paper should be described in detail. E.g. having depression (a mental health condition) and self-efficacy under the same domain is confusing. A clear operational definition is required.

We have provided clear operational definitions for mental health outcomes, and separated it from stigma outcomes. We hope this aids comprehension.

B. Results: Need more conceptual clarity on the eight outcomes. e.g. Recovery is a very vague term that means different thing to different people. The authors note that in the discussion, but they are also requested to discuss how it was conceptualized in the studies they reviewed. This should help in making the results more cohesive. Social distance scale is also often used as a measure of stigma – can the authors discuss how the studies have used/defined it so that it can be presented as a different domain from stigma?

We believe we have clearly distinguished personal recovery from functional recovery in the discussion section of the manuscript, and have delineated social distancing and perception of danger as outcomes of stigma in HCPs, and stigma resistance in PLWMI. Again, we appreciate that stigma is an issue that cannot be comprehensively exhausted in one paper as there are many seminal works published on this, and hope that the new way in which stigma has been presented in the new manuscript aids overall comprehension and cohesiveness. We have also expanded the characteristics of included studies to encompass the different ways in which the articles have conceptualized stigma.

Table 1 needs more clarity – E.g. Zhang 2023: uses social distance scale but the outcome is better caregiving experience and better recovery. In Russinova 2014 the questionnaire used by the study is ISMI and coping with stigma, but outcome is more than that including depression. In the table, it is important to note what measure was used for depression? This makes reading the table confusing. The authors are requested to revise the table making it more specific and detail and subsequently reflect that while describing the result section.

We have expanded the table to reflect and address these concerns.

C. Discussion: The authors have initiated good discussion points but needs to be tighter. Some suggested papers to **support this section and introduction.**
a. On methods:
• Abayneh 2022 paper: https://link.springer.com/article/10.1186/s12913-022-08290-x
• Rai 2023 paper: https://www.cambridge.org/core/journals/global-mental-health/article/photovoice-method-for-collaborating-with-people-with-lived-experience-of-mental-health-conditions-to-strengthen-mental-health-services/4FA9A30F1C6DF9F07FE9F952403F20FB

b. On theory: Kohrt 2020 paper https://www.sciencedirect.com/science/article/abs/pii/S027795362030071X

D. Other comments
a. One study to my knowledge I see missing here, though cited is Kohrt 2021 JAMA paper on RESHAPE RCT – doi: 10.1001/jamanetworkopen.2021.31475
Was it excluded because it was a pilot RCT and not a full RCT?

We have tweaked sections of the discussion with a view to making it tighter. We have considered the recommended references, but still feel the current references we have used pretty much reflect the same thoughts to the references that have been already included and we greatly appreciate the broader reading and knowledge provided by these references.

The Khort 2021 paper is a pilot RCT and on further reflection, we have decided to include it and repeat the meta-analyses where appropriate.

Overall, thank you for the insights as we believe it has greatly enhanced the rigour and internal validity of our meta-analysis.

Sincerely,
Ugochukwu Aghalu [MPH]
Department of Nursing and Community Health,
Glasgow Caledonian University.
Phone: +447867313820.
Email: UAGHAL300@caledonian.ac.uk

Additional Contact,
Dr Adeniyi Adeboye [PhD],
Senior lecturer,
Department of Nursing and Community Health,
Glasgow Caledonian University.
Phone: +447960266152
Email: adeniyi.adeboye@gcu.ac.uk
