# Supplementary material for: The impact of photovoice on mental health and stigma: A systematic review and meta-analysis
Source: PLOS Glob Public Health. 2025 Jul 22;5(7):e0004272. doi: 10.1371/journal.pgph.0004272 (PMC12282929; doi:10.1371/journal.pgph.0004272)
Supplement: Attachment — Submitted filename: Rebuttal letter to reviewers.docx [file pgph.0004272.s011.docx]

Ugochukwu Aghalu [MPH] Glasgow Caledonian University, Cowcaddens Road,

Glasgow, United Kingdom G4 0BA, [[UAGHAL300@caledonian.ac.uk](mailto:UAGHAL300@caledonian.ac.uk)]

The Editorial team, *PLOS GLOBAL PUBLIC HEALTH.*

[6^th^ July, 2024]

**Rebuttal letter to the reviewers**

Reviewer 1:

Thank you for taking your time to thoroughly review the manuscript.

**Abstract: Please follow Prisma 2020 guidelines for the reporting of systematic review abstract.**

We have endeavoured to follow the PRISMA 2020 guidelines in the new abstract that we have provided in the revised manuscript.

**Pg4, p2: study evaluates the impact of photovoice in mental ill health: vague and not clear based on included papers. The abstract mentions the evaluation of the effects of photovoice on 8 mental health outcomes. For clarity, it would be helpful if authors separate mental health outcomes and stigma outcomes as different.

Pg5 p2: “what is the impact of photovoice on mental health outcomes?” – Similar to above comment, it would be great if authors could modify this into the impact of photovoice on mental health and stigma outcomes of PLWMI and HCPs.**

We have incorporated these great points into the new manuscript, separating outcomes into mental health and stigma outcomes.

**Pg6: Search strategy- The authors need to include full search strategies used for all databases including any filters and limits used. (See Prisma guideline for reporting)- I saw that authors have included prisma 2009 checklist- this need to be referenced in the main manuscript and as there is prisma guideline/checklist for 2020, it would be great if authors updated the checklist.**

The full search strategy for all databases searched have been updated. Likewise, the PRISMA checklist has been updated to 2020.

**Table 1: Characteristics of included studies- it is confusing as some studies only had PLWMIs some only had HCPs while some had both as population and it is also not clear which outcomes/questionnaires were used for which population? Would be helpful if the authors added a column on population for the studies.
Another useful thing to mention in the table is the outcome measure as per your analysis. Currently the outcomes listed in the table does not match with the outcomes you report. How were the listed outcomes such as dangerousness, social desirability, empowerment, coping with stigma etc categorized into the outcomes you report on later?**

The characteristics of included studies have been expanded accordingly for more comprehension. All the authors were consulted in the process of pulling and logically matching outcomes from studies to be able to carry out meta-analysis, and that is how we arrived at the outcomes for this current meta-analysis.

**Pg 19, 20: The data analysis section should go into the methods section rather than the results.**

This has been addressed in the new manuscript.

**Pg 21: The effects of photovoice on stigma resistance and social distance of PLWMIs is not clear. Social distance is used to measure public/provider stigma towards PLWMIs – not with PLWMIs. Is this social withdrawal instead of social distance?

Also, how was stigma resistance measured- I don’t see stigma resistance mentioned in any of the outcomes or questionnaires you have listed in Table 1? Is this stigma coping?

The papers listed under PLWMI outcomes of anger, recovery, social distance, etc. (Flanagan 2016, Tippin and Maranzan 2019) are targeting health workers rather than PLWMI- I am not sure how the authors assigned the health workers' outcomes to PLWMIs?**

We have repeated the meta-analysis and assigned the correct papers to the correct populations in the new manuscript. We have also evidenced the measure of stigma resistance in Table 1, so we believe these points have been addressed.

**Pg 25 I think there were a lot more papers on health worker stigma outcomes- as mentioned in the study characteristics (table 1), however, the authors have only described the effects of Tergesen's 2021 paper on the effect of photovoice on the healthcare provider section. In addition, the Tergesen paper mentions an increase in stigma scores among medical students after watching the psychosis photovoice video. This is not reflected in the findings of the current paper.**

We have decided to remove the Tegersen’s paper due to its unsuitability for meta-analysis and hone in on the outcomes that can be verifiable by conducting thorough meta-analysis. Addressing the initial point, we have re-assigned the right papers to the right population, and expanded the characteristics table to reflect populations.

**On a similar note, the paper Zhang et al 2023, the study uses mindfulness-based psychoeducation as an intervention with a component of photovoice and its effects on YAIR. The author’s interpretation that this is the effect of photovoice rather than the effect of mindfulness-based psychoeducation might be a bit farfetched.**

Firstly, we have included a discussion in the introduction that photovoice can be utilized as both an intervention, and a method, and backed it up with relevant references.

Additionally, the paper, Zhang et al., 2023 utilised the methodology of photovoice to deliver the intervention. We refer to the appendix in the study that demonstrates that photovoice was consistently utilized over the duration of the study in all five phases of the intervention administered by the Mindfulness-Based Family Psychoeducation (MBFBE) (Arm 1). Furthermore, the detailed discussion of how the methodology of photovoice was utilized in this study to deliver the MBFBE is provided in the qualitative aspect of the study in Lo et al. (2022).

Lo HH, Liu KH, Ho WC, Lau EN, Poon MF, Lo CS, et al. Using Photovoice in a Mindfulness-Based Program to Understand the Experiences of Caregivers of Young Adults with Psychosis. International Journal of Environmental Research and Public Health. 2022; 19(23) doi:10.3390/ijerph192315461.

Overall, we thank you for the hugely insightful feedback as we have utilized this to thoroughly improve the rigour and internal validity of our meta-analysis.

Reviewer 2:

Thank you for taking your time to thoroughly review our manuscript.

**A. Introduction: The authors talk about the rationale and advantages of Photovoice but more detail on the content and process of Photovoice is required – e.g. how are the participants selected, trained, how do they write stories and take their photos and how are they presented across different settings. This should help in setting up the background of the study.**

We have taken our time to incorporate the expanded discussion of photovoice into the introduction section of the new manuscript.

**“Mental health outcomes” – that the author describes in the abstract and rest of the paper should be described in detail. E.g. having depression (a mental health condition) and self-efficacy under the same domain is confusing. A clear operational definition is required.**

We have provided clear operational definitions for mental health outcomes, and separated it from stigma outcomes. We hope this aids comprehension.

**B. Results: Need more conceptual clarity on the eight outcomes. e.g. Recovery is a very vague term that means different thing to different people. The authors note that in the discussion, but they are also requested to discuss how it was conceptualized in the studies they reviewed. This should help in making the results more cohesive.
Social distance scale is also often used as a measure of stigma – can the authors discuss how the studies have used/defined it so that it can be presented as a different domain from stigma?**

We believe we have clearly distinguished personal recovery from functional recovery in the discussion section of the manuscript, and have delineated social distancing and perception of danger as outcomes of stigma in HCPs, and stigma resistance in PLWMI. Again, we appreciate that stigma is an issue that cannot be comprehensively exhausted in one paper as there are many seminal works published on this, and hope that the new way in which stigma has been presented in the new manuscript aids overall comprehension and cohesiveness. We have also expanded the characteristics of included studies to encompass the different ways in which the articles have conceptualized stigma.

**Table 1 needs more clarity – E.g. Zhang 2023: uses social distance scale but the outcome is better caregiving experience and better recovery. In Russinova 2014 the questionnaire used by the study is ISMI and coping with stigma, but outcome is more than that including depression. In the table, it is important to note what measure was used for depression? This makes reading the table confusing. The authors are requested to revise the table making it more specific and detail and subsequently reflect that while describing the result section.**

We have expanded the table to reflect and address these concerns.

C. Discussion: The authors have initiated good discussion points but needs to be tighter. Some suggested papers to **support this section and introduction.
a. On methods:
• Abayneh 2022 paper: https://link.springer.com/article/10.1186/s12913-022-08290-x
• Rai 2023 paper: https://www.cambridge.org/core/journals/global-mental-health/article/photovoice-method-for-collaborating-with-people-with-lived-experience-of-mental-health-conditions-to-strengthen-mental-health-services/4FA9A30F1C6DF9F07FE9F952403F20FB
b. On theory: Kohrt 2020 paper https://www.sciencedirect.com/science/article/abs/pii/S027795362030071X

D. Other comments
a. One study to my knowledge I see missing here, though cited is Kohrt 2021 JAMA paper on RESHAPE RCT – doi: 10.1001/jamanetworkopen.2021.31475
Was it excluded because it was a pilot RCT and not a full RCT?**

We have tweaked sections of the discussion with a view to making it tighter. We have considered the recommended references, but still feel the current references we have used pretty much reflect the same thoughts to the references that have been already included and we greatly appreciate the broader reading and knowledge provided by these references.

The Khort 2021 paper is a pilot RCT and on further reflection, we have decided to include it and repeat the meta-analyses where appropriate.

Overall, thank you for the insights as we believe it has greatly enhanced the rigour and internal validity of our meta-analysis.

Sincerely,	Additional Contact,

Ugochukwu Aghalu [MPH]	Dr Adeniyi Adeboye [PhD],

Department of Nursing and Community Health,	Senior lecturer,

Glasgow Caledonian University.	Department of Nursing and Community Health,

Phone: +447867313820.	Glasgow Caledonian University.

Email: [UAGHAL300@caledonian.ac.uk](mailto:UAGHAL300@caledonian.ac.uk)	Phone: +447960266152

Email: [adeniyi.adeboye@gcu.ac.uk](mailto:adeniyi.adeboye@gcu.ac.uk)
