# Supplementary material for: The impact of photovoice on mental health and stigma: A systematic review and meta-analysis
Source: PLOS Glob Public Health. 2025 Jul 22;5(7):e0004272. doi: 10.1371/journal.pgph.0004272 (PMC12282929; doi:10.1371/journal.pgph.0004272)
Supplement: Attachment — Submitted filename: Rebuttal letter to reviewers (1).docx [file pgph.0004272.s012.docx]

Ugochukwu Aghalu [MPH] Glasgow Caledonian University, Cowcaddens Road,

Glasgow, United Kingdom G4 0BA, [[UAGHAL300@caledonian.ac.uk](mailto:UAGHAL300@caledonian.ac.uk)]

The Editorial team, *PLOS GLOBAL PUBLIC HEALTH.*

[19^th^ September, 2024]

**Rebuttal letter to the reviewers**

Reviewer 3:

Thank you for taking the time to thoroughly review the manuscript.

**Question 1;** I congratulate the authors for addressing adequately the comments raised in the previous review round.
Upon reviewing the references, I have observed ref number 72 to be incorrectly cited The words "Link to external site this link will open in a new window" should be omitted.

Response: The reference has been corrected and adjusted, and all included references have been re-worked. Overall, we thank you for the insightful feedback as we have utilized this to thoroughly improve the rigour and internal validity of our meta-analysis.

Reviewer 4:

Thank you for taking the time to thoroughly review our manuscript.

>Major Points:

**Question: Relevance and Novelty:**
Your study is highly relevant and novel, particularly in its exclusive focus on photovoice's impact on both PLWMI and HCPs. It fills a critical gap in the existing literature. However, I suggest emphasizing this point more clearly in the introduction, especially when discussing the study's significance and how it advances the current knowledge.

**Response:** We have dedicated a section titled study rationale in the updated introduction within which the study gaps have been identified, added several references and have made both the novelty and relevance particularly clear for the reader.

**Question:** Clarification of Terms and Concepts:
Some key concepts, such as personal recovery versus functional recovery, would benefit from clearer definitions and distinctions. This will help readers unfamiliar with these terms better understand the implications of your findings.

**Response:** Recovery has been expounded on in the introduction, and we have categorized and expanded this to include clinical, functional, and personal recovery, adding more references to back this up.

**Question: Heterogeneity and Study Quality:**
While you acknowledge the high heterogeneity in certain outcomes (e.g., anger, perception of danger, and social distance), a more detailed discussion on how this heterogeneity might affect the interpretation of your findings would be beneficial. Additionally, consider discussing potential strategies to address this issue in future research.

**Response:** This has been detailed and addressed, in the section titled Limitations of the study with added references.

**Question: Discussion of Stigma Resistance:**
The reduction in stigma resistance following photovoice intervention is an intriguing and somewhat counterintuitive finding. The explanation provided—related to the short duration of the studies analyzed—could be expanded. I recommend exploring alternative explanations or discussing the potential impact of the intervention's context on this outcome.

**Response:** This has been also expanded and addressed in its relevant section, with added examples and references included.

**Question: Implications for Practice:**
While you provide a solid discussion on the implications for mental health services, more specific examples or recommendations on how photovoice can be integrated into current practices would enhance the practical utility of your findings. This could include concrete steps for implementation in various healthcare settings.

**Response:** This has been also expanded and addressed in its relevant section, with added examples and references included.

>Minor Points:
**Formatting:**
Ensure consistency in the formatting of tables and figures. Some figures appear to have formatting issues that should be corrected to improve the overall presentation.

**Response:** The tables and figures have been uniformly formatted in this resubmission.

**Literature Review:**

The introduction could be strengthened by providing a brief overview of key studies that have previously explored photovoice’s impact on mental health. This would establish a stronger foundation for your study and highlight the specific gaps your research addresses.

**Response:** This has been detailed in the rationale section.

Statistical Analysis:

The choice of random-effect models and the handling of potential publication bias are appropriate, but further clarification in these areas would add to the robustness of your analysis. Consider providing a brief explanation of these choices in the methods or results section.

We have added the implication of the random-effect model and provided an expanded rationale for this choice in the methods. A section titled publication bias has been added to the results section as well, and these have been backed up with more relevant references.

I recommend minor revisions to address these points for the final version of your manuscript.

Sincerely,	Additional Contact,

Ugochukwu Aghalu [MPH]	Dr Adeniyi Adeboye [PhD],

Department of Nursing and Community Health,	Senior lecturer,

Glasgow Caledonian University.	Department of Nursing and Community Health,

Phone: +447867313820.	Glasgow Caledonian University.

Email: [UAGHAL300@caledonian.ac.uk](mailto:UAGHAL300@caledonian.ac.uk)	Phone: +447960266152

Email: [adeniyi.adeboye@gcu.ac.uk](mailto:adeniyi.adeboye@gcu.ac.uk)
