# Supplementary material for: The impact of photovoice on mental health and stigma: A systematic review and meta-analysis
Source: PLOS Glob Public Health. 2025 Jul 22;5(7):e0004272. doi: 10.1371/journal.pgph.0004272 (PMC12282929; doi:10.1371/journal.pgph.0004272)
Supplement: Attachment — Submitted filename: response to reviewers file.docx [file pgph.0004272.s013.docx]

JOURNAL REQUIREMENTS:

1. Please ensure that the author list and affiliations are correct on the title page of your manuscript, and that your author contributions, competing interests, and financial disclosure are correct as listed below. All of these sections will be indexed in PubMed and published by PLOS Global Public Health as you have written them. Please email globalpubhealth@plos.org if any changes to this content needs to be made.

Name: Adeniyi Adeboye
Contributions: Conceptualization
Data curation
Formal analysis
Investigation
Methodology
Project administration
Software
Supervision
Validation
Visualization
Writing – original draft

Name: Ugochukwu Aghalu
Contributions: Formal analysis
Software
Visualization
Writing – original draft
Writing – review & editing

Name: Whitney Onuorah
Contributions: Data curation
Investigation
Methodology
Validation
Visualization
Writing – original draft
Writing – review & editing

Name: Chizor Samuel-Nwokeji
Contributions: Data curation
Investigation
Methodology
Visualization
Writing – original draft
Writing – review & editing

Name: Chimaobi Nwanguma
Contributions: Data curation
Formal analysis
Methodology
Validation

Name: Adekunle Akerele
Contributions: Data curation
Formal analysis
Software
Validation
Visualization
Writing – review & editing

Name: Judy Wasige      
Contributions: Conceptualization
Funding acquisition
Project administration
Resources
Supervision
Visualization
Writing – review & editing

**Response: This has been reviewed, and no changes need to be made.**

2. Competing Interests:

The authors have read the journal’s policy and hereby declare no financial or non-financial conflicts of interest.

We have amended your Competing Interest statement to comply with journal style. We kindly ask that you double check the statement and let us know if anything is incorrect.

**Response: This has been double-checked. Everything is correct.**


3. Please provide a detailed Financial Disclosure statement. This is published with the article. It must therefore be completed in full sentences and contain the exact wording you wish to be published.

**Please only choose the relevant sentences from below**

Financial Disclosure:
This study was funded by a grant from See Me Scotland (URL: [https://www.seemescotland.org/)](https://www.seemescotland.org/). There was no specific number associated with the grant. JW through ILFA Project Charity, was the specific recipient of the award that was designed as a community engagement project. See Me Scotland’s understanding of the community engagement process provided an enabling environment that facilitated the integration of a research element into the community project. CN, WO, and CS received compensation from JW. “The funders had no role in study design, data collection and analysis, decision to publish, or preparation of the manuscript.”

**Response: This has been addressed.**


4. Your current Financial Disclosure must be the same as your funding information on the submission. Please indicate by return the full and correct funding information for your study and confirm the order in which they should appear. Please be sure to indicate whether the funders played any role in the study design, data collection and analysis, decision to publish, or preparation of the manuscript.

Financial Disclosure:
This study was funded by a grant from See Me Scotland (URL: [https://www.seemescotland.org/)](https://www.seemescotland.org/). There was no specific number associated with the grant. JW through ILFA Project Charity, was the specific recipient of the award that was designed as a community engagement project. See Me Scotland’s understanding of the community engagement process provided an enabling environment that facilitated the integration of a research element into the community project. CN, WO, and CS received compensation from JW. “The funders had no role in study design, data collection and analysis, decision to publish, or preparation of the manuscript.”

**Response: This has been addressed.**

5.  We note that your Data Availability Statement is currently as follows: “All necessary data have been included as part of the manuscript submission.”

Please confirm at this time whether or not your submission contains all raw data required to replicate the results of your study. Authors must share the “minimal data set” for their submission. PLOS defines the minimal data set to consist of the data required to replicate all study findings reported in the article, as well as related metadata and methods (<https://journals.plos.org/plosone/s/data-availability#loc-minimal-data-set-definition>).

For example, authors should submit the following data:

- The values behind the means, standard deviations and other measures reported;
- The values used to build graphs;
- The points extracted from images for analysis.

Authors do not need to submit their entire data set if only a portion of the data was used in the reported study.

If your submission does not contain these data, please either upload them as Supporting Information files or deposit them to a stable, public repository and provide us with the relevant URLs, DOIs, or accession numbers. For a list of recommended repositories, please see <https://journals.plos.org/plosone/s/recommended-repositories>.

If there are ethical or legal restrictions on sharing a de-identified data set, please explain them in detail (e.g., data contain potentially sensitive information, data are owned by a third-party organization, etc.) and who has imposed them (e.g., an ethics committee). Please also provide contact information for a data access committee, ethics committee, or other institutional body to which data requests may be sent. If data are owned by a third party, please indicate how others may request data access.

Response: This has been cross-checked and all necessary data to facilitate replication has been included.


6. As required by our policy on Data Availability, please ensure your manuscript or supplementary information includes the following:
1. A numbered table of all studies identified in the literature search, including those that were excluded from the analyses.
a. For every excluded study, the table should list the reason(s) for exclusion.
b. If any of the included studies are unpublished, include a link (URL) to the primary source or detailed information about how the content can be accessed.
2. A table of all data extracted from the primary research sources for the systematic review and/or meta-analysis. The table must include the following information for each study:
a. Name of data extractors and date of data extraction
b. Confirmation that the study was eligible to be included in the review.
c. All data extracted from each study for the reported systematic review and/or meta-analysis that would be needed to replicate your analyses.
If data or supporting information were obtained from another source (e.g. correspondence with the author of the original research article), please provide the source of data and dates on which the data/information were obtained by your research group.
3. A table showing the completed risk of bias and quality/certainty assessments for each study or outcome. Please ensure this is provided for each domain or parameter assessed. For example, if you used the Cochrane risk-of-bias tool for randomized trials, provide answers to each of the signalling questions for each study. If you used GRADE to assess certainty of evidence, provide judgements about each of the quality of evidence factor. This should be provided for each outcome.
4. An explanation of how missing data were handled.

This information can be included in the main text, supplementary information, or relevant data repository. Please note that providing these underlying data is a requirement for publication in this journal, and if these data are not provided your manuscript might be rejected.

**Response: This has been addressed. The numbered table of all identified studies can be found in S4_Appendix. This table also includes confirmation that all included studies were eligible to be included in the review. The data extracted for the meta-analysis can be found in S3 and S4_Tables. The quality assessment table based on EPHPP can be found in the body of the manuscript. We did not use GRADE to assess the certainty of the evidence. We did not encounter any missing data in our process.**


7. Please upload your main article file as a .doc, .docx or .rtf file.

**Response: This has been addressed.**


8. Please ensure that all author names are spelled correctly in the manuscript file, including the use of a middle name/initials, and any special characters (accents, umlauts, etc.). Please place first/given names before last/family names. All authors must have a first and last name. We noticed that Ugochukwu Aghalu is spelled differently between the manuscript file and submission form. Please be aware that correct spelling and formatting of author names in the article file is vital for accurate indexing in PubMed. Please let us know if you would like us to amend the spelling in Editorial Manager on your behalf.

**Response: This has been addressed.**

9. Please ensure that your manuscript contains an Abstract at the start of the text, under the heading 'Abstract'. Abstracts should not exceed 300 words, and they should not contain headings or citations.

**Response: This has been addressed.**


10. We note that you have included a declarative (Funding, Data,etc.) statement within the body of your manuscript. Please note that declarative statement should not be included within the manuscript, and we ask that you remove them.

**Response: This has been addressed, and the unnecessary information removed.**

11. We note that your in-text citations are not in accordance with our formatting guidelines. In the text, cite the reference number in square brackets.

**Response: This has been addressed.**


12. We note that you have included the phrase “data not shown” or "unpublished data" on page 1. Unfortunately, this does not meet our data sharing requirements. PLOS does not permit references to inaccessible data. We require that authors provide all relevant data within the paper, Supporting Information files, or in an acceptable, public repository. Please add a citation to support this phrase or upload the data that corresponds with these findings to a stable repository (such as Figshare or Dryad) and provide and URLs, DOIs, or accession numbers that may be used to access these data. Or, if the data are not a core part of the research being presented in your study, we ask that you remove the phrase that refers to these data.

**Response: This has been addressed. There is no unpublished data that has been included in the manuscript.**

13. Please remove the funding information from the Acknowledgements section of your manuscript file. The published version is pulled automatically from the Financial Disclosure section of the submission form.

Please send us an updated Financial Disclosure statement, we will update the submission system on your behalf.

**Response: This has been addressed.**


14. Your references need to be formatted according to the ICMJE standard reference style. PLOS uses the numbered citation [citation-sequence] method and first six authors, et al. Authors should be listed as "Last name, First Initial". Please note that references should be cited in square [ ] brackets and not round ( ) brackets to ensure that they are correctly typeset. Additionally, the reference list should be formatted as “1.” and not “[1]”. Please adhere to the PLOS style for all references and include any urls as appropriate. For a full outline see our Author Guidelines:

<https://journals.plos.org/globalpublichealth/s/submission-guidelines#loc-references>

**Response: This has been addressed.**

15. Please ensure that all main figure files in the inventory follow PLOS figure naming conventions: the file name should be in the format "Fig#.filetype": for example, Fig1.tif or Fig1.eps.

In the inventory's Item Description field and in all in-text references, figures should be referred to as "Fig 1," Fig 2," "Fig 1A," etc.

Please note that PLOS does NOT allow the following formatting:
- Figure 1 or figure 1
- Fig. 1
- fig 1

**Response: This has been addressed, and all figure descriptions formatted.**

16. Please ensure that all supporting information file names use the following format verbatim in the File Inventory including capitalization. The file names themselves must use an underscore rather than a space, i.e.
S1_Fig.tif
S1_Table.xlsx
S1_Text.doc
S1_Data.xlsx
S1_PRISMA_Checklist.docx etc

Each file type should be numbered starting at 1. Please also make sure that the file description matches the file name (e.g. S1_Fig.tif is listed as S1 Fig).

**Response: This has been addressed, and the supplementary information has been formatted accordingly.**

17. Please note that you've included reference citations in file S1 Table and S2 Appendix. Since each supporting information file is self-contained, we ask that you please list all cited references in full in that specific file under a 'Reference' heading and start numbering the citations at 1.

**Response: This has been addressed in the respective supplementary files.**
